# A new computational approach to analyze human protein complexes and predict novel protein interactions

**DOI:** 10.1186/gb-2007-8-12-r256

**Published:** 2007-12-04

**Authors:** Sara Zanivan, Ilaria Cascone, Chiara Peyron, Ivan Molineris, Serena Marchio, Michele Caselle, Federico Bussolino

**Affiliations:** 1 Department of Oncological Sciences and Division of Molecular Angiogenesis, Institute for Cancer Research and Treatment (IRCC), University of Torino Medical School, Strada Provinciale, I-10060 Candiolo (Turin), Italy; 2 Max-Planck Institute for Biochemistry, Department of Proteomics and Signal Transduction, Am. Klopferspitz, D-82152 Martinsried, Germany; 3 Inserm U528, Institut Curie, 75248 Paris, France; 4 Department of Theoretical Physics, University of Torino and INFN, Via P Giuria 1, I-10125 Turin, Italy

## Abstract

We propose a new approach to identify interacting proteins based on gene expression data. By using hypergeometric distribution and extensive Monte-Carlo simulations, we demonstrate that looking at synchronous expression peaks in a single time interval is a high sensitivity approach to detect co-regulation among interacting proteins. Combining gene expression and Gene Ontology similarity analyses enabled the extraction of novel interactions from microarray datasets. Applying this approach to p21-activated kinase 1, we validated α-tubulin and early endosome antigen 1 as its novel interactors.

## Abstract 2

A new approach to identifying interacting proteins based on gene-expression data uses hypergeometric distribution and Monte-Carlo simulations.

## Background

The cell is a complex system involving a heterogeneous and highly dynamic set of proteins whose ability to interact and form complexes is critical for cellular activity and regulation [1]. A major goal, therefore, is the complete identification of the interactome. Different high-throughput experimental approaches have been developed to characterize the interactomes of several organisms. Yeast two hybrid screens allow binary interactions to be defined while tandem affinity purification (TAP)-tag followed by mass spectrometry analysis is used to purify and identify components of multi-protein complexes [2-5]. Up to now, data have been mostly generated by studying simple organisms such as *Saccharomyces cerevisiae*, *Caenorhabditis elegans *and *Drosophila melanogaster *[6,7]. For human cells, published experimental results are collected in databases like MINT (Molecular Interactions database) and HPRD (Human Protein Reference Database) [8,9], but the amount of information is still largely limited. Moreover, data have been obtained from different cellular models and using different techniques, thus rendering it difficult to build a global network of interactions or to extrapolate information about the composition of multi-protein complexes.

Computational approaches may help to address these crucial issues [10-17]. The current idea is that proteins forming a supra-molecular complex are transcribed simultaneously and standard Pearson's analysis has been extensively applied to gene expression datasets to support this concept [12,14,15,17,18]. In general, good results are obtained with this method if protein interactions of stable protein complexes are studied, but it is less efficient in other cases [12,14]. A paradigmatic example is the application of Pearson's analysis to gene expression datasets of the yeast cell-cycle. A strong and significant correlation can be obtained for permanent protein complexes, but only weak correlations are seen for the transient ones [14]. A similar conclusion resulted from the analysis of some human gene profiles [12].

In this paper we present a new approach for the detection of putative protein interactions based on expression data. Besides the identification of permanent complexes, it is also capable (at least for well synchronized samples) of reliably identifying interactions among proteins belonging to transient complexes. This approach is based on two observations. Firstly, protein-protein interactions are more easily identified if the interacting protein pair belongs to a multi-protein complex. This is a direct consequence of the fact that the features used to identify the interactions (that is, correlations in expression data) display a much higher signal to noise ratio if multiple correlations are looked for simultaneously. Therefore, we focused on tracking interactions within protein complexes, even though our algorithm can, in principle, identify any type of protein-protein interaction. The second observation is that while Pearson's correlators are very effective at identifying permanent complexes, which remain assembled throughout most experimental time-points, they are less suitable for transient complexes, which are assembled for only one or a few time-points. To overcome this problem, we propose a new method to extract putative human interacting proteins from microarray gene expression data by looking at the presence of synchronous expression peaks in time course experiments of synchronized HeLa cells [19]. This is further supported by the recent observation in yeast that the timing of transcription during the cell-cycle is indicative of the timing of protein complex assembly [20].

This approach allowed us to address interactions characterized by low, but not negligible, statistical significance, which would instead be completely filtered out in the Pearson-based analysis. To further enhance the signal to noise ratio we combined this analytical procedure with a standard Gene Ontology (GO) [21] search. This filter turns out to be very effective, since it is based on input information completely independent from data exploited in the previous analysis step.

To test the performance of our approach and compare it with the standard Pearson-based one, we established and tested a set of 32 permanent and transient complexes. The application of our method shows its effectiveness in detecting protein interactions in permanent and transient complexes. We also observed that, as expected, the proposed technique performs better as the synchronization of the dataset improves. To specifically test the applicability of our method in a precise biological context, we used it to explore novel putative interacting partners for serine/threonine p21-activated kinase (PAK)1. PAK1 is a kinase downstream of the Rho family of small GTPases, which participates in the formation of several dynamic and transient transductosomes [22]. We also provide experimental evidence confirming the interactions predicted by our algorithm between PAK1 and α-tubulin as well as PAK1 and early endosome antigen (EEA)1, a coiled coil dimer that is crucial for endosome fusion *in vitro *[23].

## Results

### Starting data: known protein complexes and microarray datasets

Up to now there are no databases for genome-wide multi-protein interactions in mammals. Thus, we focused our study on 11 permanent and 21 transient human complexes of different sizes that are well characterized in the literature (Table 1, and see Materials and methods). Since transient complexes display dynamic properties, we analyzed microarray data from several temporal series describing a dynamic cellular condition. For this, we selected from the Stanford Microarray Database [24] three independent datasets analyzing the cell-cycle of HeLa cells synchronized either with double thymidine (Thy-Thy) or thymidine-nocodazole (Thy-Noc). In particular, only data from the first full cell-cycle (14 hours long) after synchronization were considered.

**Table 1:** Set of known human multi-protein complexes analyzed

		Number of genes		
		
Protein complex	Complex type	Thy-Thy2	Thy-Thy3	Thy-Noc
ATP_F0	Permanent	7	10	10
ATP_F1	Permanent	4	3	3
COX	Permanent	7	6	8
SRS	Permanent	16	20	20
LRS	Permanent	15	18	18
MLRS	Permanent	22	36	37
MSRS	Permanent	20	30	30
Proteasome	Permanent	21	21	23
PD	Permanent	6	7	7
RNA Pol II	Permanent	10	10	10
RNA Pol III	Permanent	4	6	5
AP2	Transient	2	4	4
APC	Transient	5	8	8
Arp2-3	Transient	6	3	5
ARC	Transient	4	5	5
Centrosome	Transient	42	50	51
Dynactin	Transient	7	9	7
Exocyst	Transient	7	7	7
Exosome	Transient	3	5	5
FA	Transient	37	46	47
GTC	Transient	5	6	6
Nucleopore	Transient	27	29	30
Nucleosome	Transient	17	24	24
ORC	Transient	4	5	6
RFC	Transient	3	4	4
SRP	Transient	3	5	4
SCF	Transient	3	3	3
SNARE complex	Transient	7	7	7
SWI-SNF	Transient	12	10	12
TAFIID	Transient	8	13	13
TRAPP	Transient	2	5	6
VHL	Transient	4	4	4

The number of genes representing each protein complex is reported for the three analyzed HeLa cell-cycle datasets. AP2, adaptor-related protein complex 2; APC, anaphase promoting complex; ARC, axin related complex; ATP_F0, ATP synthase, H+ transporting, mitochondrial F0 complex; ATP_F1, ATP synthase, H+ transporting, mitochondrial F1 complex; COX, cytochrome c oxidase; FA, focal adhesion; GTC, golgi transport complex; MSRS, mitochondrial small ribosomal subunit; ORC, origin recognition complex; PD, pyruvate dehydrogenase; RNA Pol II, RNA polymerase II; SRP, signal recognition particle; TRAPP, trafficking protein particle complex; VHL, von Hippel-Lindau complex.

### Gene expression analysis of human protein complexes by Pearson correlation coefficient

To extract putative protein-protein interactions from gene expression data, we first evaluated the Pearson's correlation for each pair of genes in the above described HeLa datasets. To assess if the number of highly correlated components had been obtained by chance, results were compared with the global behavior of the dataset by a standard hypergeometric test (Materials and methods).

Among the 32 analyzed protein complexes, 23 showed a *p *value lower than 0.05, including 5 in Thy-Thy dataset 2 (Thy-Thy2; Additional data file 1a), 10 in Thy-Thy dataset 3 (Thy-Thy3; Additional data file 1b) and 8 in the Thy-Noc dataset (Table 2). Among them (in particular in the very low *p *value range), a dominance of permanent with respect to transient protein complexes was observed. As an example, proteasome and small ribosomal subunit (SRS), which are well known stable complexes, were both characterized by very low *p *values in at least two datasets. However, we also found several complexes in which the number of highly correlated genes was clearly not statistically significant (that is, with a *p *value ≥ 0.7). In particular, this occurred in 15, 11 and 12 complexes in the Thy-Thy2, Thy-Thy3, and Thy-Noc datasets, respectively, including both permanent and transient complexes. RNA polymerase III is an example of a permanent complex without a significant *p *value in all three datasets.

**Table 2:** P values for Thy-Noc dataset

	Peaks of expression (*p *value)							Pearson (*p *value)
		
Protein complex	2 h-0 h	4 h-2 h	6 h-4 h	8 h-6 h	10 h-8 h	12 h-10 h	14 h-12 h	Cell-cycle
AP2	3.67E-01	7.47E-01	7.05E-01	1.00E+00	4.78E-01	6.26E-01	3.73E-01	1.00E+00
ARC	3.49E-02	1.00E+00	1.00E+00	6.95E-03	1.00E+00	7.28E-02	1.00E+00	1.00E+00
Arp2-3	1.00E+00	1.51E-01	3.95E-01	1.00E+00	5.56E-01	1.00E+00	5.00E-01	1.00E+00
ATP_F0	1.00E+00	9.68E-01	9.53E-01	6.30E-01	8.03E-01	1.00E+00	2.21E-03	3.29E-03
ATP_F1	1.00E+00	6.43E-01	1.00E+00	4.92E-01	1.00E+00	1.00E+00	6.77E-01	1.00E+00
APC	2.14E-01	7.26E-01	6.65E-01	2.07E-01	7.28E-01	8.60E-01	6.95E-02	7.16E-01
COX	1.00E+00	7.26E-01	3.54E-01	1.00E+00	3.43E-01	1.00E+00	2.20E-01	1.96E-06
Centrosome	7.58E-01	9.13E-01	9.90E-01	5.96E-01	7.11E-02	7.27E-02	1.46E-01	1.79E-03
Dynactin	9.26E-01	1.00E+00	8.82E-01	7.94E-01	6.80E-01	8.21E-01	3.50E-02	2.35E-01
Exocyst	6.93E-01	3.32E-01	5.87E-01	3.44E-02	6.80E-01	1.81E-01	3.85E-01	2.35E-01
Exosome	8.44E-01	8.20E-01	3.95E-01	1.00E+00	1.00E+00	1.00E+00	1.82E-01	3.62E-01
FA	3.81E-01	6.38E-01	1.49E-01	7.64E-02	9.37E-01	1.27E-01	1.21E-01	1.53E-01
GTC	6.02E-01	8.73E-01	4.97E-01	7.41E-01	2.24E-01	3.89E-01	6.10E-01	4.90E-01
LRS	9.99E-01	8.14E-01	9.96E-01	9.83E-01	9.46E-01	1.00E+00	4.42E-04	2.79E-07
MLRS	9.26E-01	6.68E-01	6.69E-01	4.78E-01	4.17E-02	9.74E-01	1.11E-04	9.41E-01
MSRS	9.40E-01	2.33E-01	2.47E-01	5.82E-01	9.52E-01	9.73E-01	3.11E-04	6.32E-03
Nucleopore	3.14E-01	3.68E-01	1.40E-01	8.82E-01	9.92E-01	3.24E-01	1.13E-03	3.60E-01
Nucleosome	9.91E-01	8.62E-03	9.71E-01	3.53E-01	8.94E-01	9.79E-01	3.28E-01	4.46E-33
ORC	2.75E-01	8.73E-01	8.40E-01	1.01E-01	6.23E-01	3.89E-01	1.00E+00	1.00E+00
PD	6.93E-01	9.10E-01	8.33E-02	4.28E-01	6.80E-01	4.73E-01	7.00E-01	6.11E-01
Proteasome	6.03E-01	9.96E-01	4.02E-01	9.62E-01	6.92E-01	1.00E+00	5.18E-08	1.99E-03
RFC	3.67E-01	1.00E+00	2.84E-01	1.84E-01	1.10E-01	6.26E-01	1.00E+00	1.00E+00
RNA Pol II	6.45E-01	5.92E-01	4.79E-03	1.00E+00	1.00E+00	1.00E+00	3.88E-01	8.68E-01
RNA Pol III	8.44E-01	1.00E+00	3.95E-01	2.66E-01	1.00E+00	7.28E-02	8.48E-01	1.00E+00
SNARE	9.26E-01	1.14E-01	5.87E-01	7.94E-01	2.83E-01	4.73E-01	9.29E-01	6.11E-01
SWI-SNF	5.40E-01	9.04E-01	3.92E-01	9.33E-01	1.00E+00	7.73E-01	3.14E-01	7.92E-01
SRP	9.19E-02	7.47E-01	7.05E-01	1.00E+00	1.00E+00	6.26E-01	9.47E-02	1.00E+00
SCF	6.72E-01	6.43E-01	6.00E-01	1.00E+00	3.86E-01	1.00E+00	3.10E-02	1.00E+00
SRS	9.94E-01	2.00E-01	2.57E-01	9.33E-01	5.95E-01	9.93E-01	7.69E-03	1.67E-11
TAFIID	8.20E-01	3.18E-01	9.81E-01	5.05E-01	3.08E-01	1.00E+00	1.96E-01	4.49E-01
TRAPP	6.02E-01	8.73E-01	1.00E+00	7.41E-01	6.23E-01	1.00E+00	2.82E-01	1.00E+00
VHL	1.00E+00	1.00E+00	2.84E-01	1.00E+00	1.00E+00	1.00E+00	3.73E-01	1.00E+00

*P *values obtained with the expression peaks method in each time interval of the cell-cycle or with Pearson correlation coefficient throughout the cell-cycle. AP2, adaptor-related protein complex 2; APC, anaphase promoting complex; ARC, axin related complex; ATP_F0, ATP synthase, H+ transporting, mitochondrial F0 complex; ATP_F1, ATP synthase, H+ transporting, mitochondrial F1 complex; COX, cytochrome c oxidase; FA, focal adhesion; GTC, golgi transport complex; MSRS, mitochondrial small ribosomal subunit; ORC, origin recognition complex; PD, pyruvate dehydrogenase; RNA Pol II, RNA polymerase II; SRP, signal recognition particle; TRAPP, trafficking protein particle complex; VHL, von Hippel-Lindau complex.

### Gene expression analysis of human protein complexes by expression peaks method

As previously observed, the Pearson-based method was unable to detect significant correlations (that is, with a *p *value not ≥ 0.7) for almost half of the tested complexes. To improve the level of detection, we set up an alternative approach, which we call the 'expression peaks method'. Gene expression was analyzed every one (for Thy-Thy datasets) or two (for the Thy-Noc dataset) hours by computing the variation of mRNA levels between consecutive time points. A threshold was then defined on computed differences, which represents the value above which we considered the increase of expression between two consecutive time points a peak of expression. Next, we placed all computed expression values in a binary 1-0 system where 1 represents an expression peak. By calculating the expression peaks for each gene along the cell-cycle in each dataset, we found that a high percentage of genes participating in the same complex peaked synchronously at least in one temporal interval (Table 3 for the Thy-Noc dataset, and Additional data file 3 for the Thy-Thy datasets). Since there was more than one peak of expression per gene, we established the peak of expression of each complex as the time interval in which the genes of the complex peaked synchronously with the best *p *value (see below). To exclude that the number of synchronously peaking genes had been obtained by chance, we performed the same analysis on the Pearson's case described above by using a hypergeometric test. Among the 32 protein complexes analyzed, 14 in Thy-Thy2, 13 in Thy-Thy3 and 13 in Thy-Noc showed a *p *value lower than 0.05 in at least one time interval along the cell-cycle. As stable complexes we detected the mitochondrial large ribosomal subunit (MLRS), SRS, the proteasome and RNA polymerase II. Interestingly, low *p *values appeared for a large number of transient protein complexes in all three datasets; dynactin, exocyst, the nucleosome, the replication complex (RFC) and the skp1-cull-F-box complex (SCF) are transient complexes with a significant *p *value in two out of three datasets (Table 2 for the Thy-Noc dataset, and Additional data file 1 for the Thy-Thy datasets). Another remarkable difference with respect to the Pearson-based method is that we never found complexes with a *p *value ≥ 0.7.

**Table 3:** Percentage of synchronously peaking genes in the Thy-Noc dataset

	Peaks of expression (% of peaking genes per complex)						
	
Protein complex	2 h-0 h	4 h-2 h	6 h-4 h	8 h-6 h	10 h-8 h	12 h-10 h	14 h-12 h
AP2	50	25	25	0	25	25	50
ARC	80	0	0	80	0	60	0
Arp2-3	0	60	40	0	20	0	40
ATP_F0	0	10	10	20	10	0	80
ATP_F1	0	33	0	33	0	0	33
APC	50	25	25	38	13	13	63
COX	0	25	38	0	25	0	50
Centrosome	27	22	14	20	24	31	39
Dynactin	14	0	14	14	14	14	71
Exocyst	29	43	29	57	14	43	43
Exosome	20	20	40	0	0	0	60
FA	34	28	34	30	9	30	40
GTC	33	17	33	17	33	33	33
LRS	6	22	6	6	6	0	72
MLRS	22	27	24	22	27	11	62
MSRS	20	37	33	20	7	10	63
Nucleopore	37	33	37	13	3	27	60
Nucleosome	13	54	13	25	8	8	38
ORC	50	17	17	50	17	33	0
PD	29	14	57	29	14	29	29
Proteasome	30	9	30	9	13	0	87
RFC	50	0	50	50	50	25	0
RNA Pol II	30	30	70	0	0	0	40
RNA Pol III	20	0	40	40	0	60	20
SNARE	14	57	29	14	29	29	14
SWI-SNF	33	17	33	8	0	17	42
SRP	75	25	25	0	0	25	75
SCF	33	33	33	0	33	0	100
SRS	10	40	35	10	15	5	60
TAFIID	23	38	8	23	23	0	46
TRAPP	33	17	0	17	17	0	50
VHL	0	0	50	0	0	0	50

For each protein complex the percentage of its synchronously peaking genes in each time interval is reported. AP2, adaptor-related protein complex 2; APC, anaphase promoting complex; ARC, axin related complex; ATP_F0, ATP synthase, H+ transporting, mitochondrial F0 complex; ATP_F1, ATP synthase, H+ transporting, mitochondrial F1 complex; COX, cytochrome c oxidase; FA, focal adhesion; GTC, golgi transport complex; MSRS, mitochondrial small ribosomal subunit; ORC, origin recognition complex; PD, pyruvate dehydrogenase; RNA Pol II, RNA polymerase II; SRP, signal recognition particle; TRAPP, trafficking protein particle complex; VHL, von Hippel-Lindau complex.

### The expression peaks method displays a higher sensitivity compared to Pearson correlation coefficient

**Figure 1:**
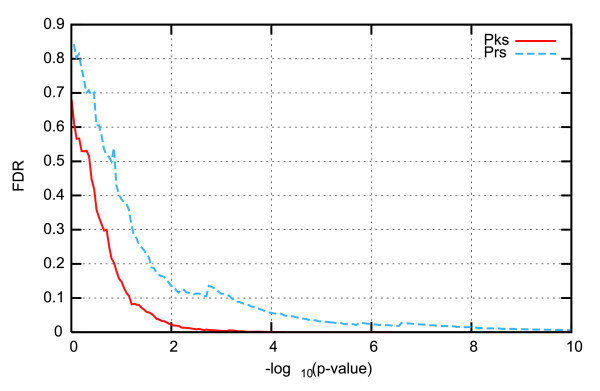
Comparison of the FDRs for the Pearson correlation coefficient (Prs) and expression peaks (Pks) methods as a function of *p *value. For the Thy-Noc HeLa cell-cycle dataset, estimated FDRs (y-axis) are reported as a function of the Bonferroni corrected *p *value (x-axis).

To assess the quality of the expression peaks method in finding co-regulated genes that encode interacting proteins, we estimated false discovery rates (FDRs; see Materials and methods, and Additional data file 2). We plotted the FDRs for the Pearson correlation coefficient and the expression peaks methods as a function of the Bonferroni corrected *p *value (Figure 1). The results from the Thy-Noc dataset (Figure 1) indicate an additional benefit of the expression peaks method. Clearly, for each *p *value, the expression peaks method displayed a smaller FDR than the Pearson method. In particular, the *p *value that corresponds to a 10% FDR for the expression peaks method (*p *= 0.1, that is, -log_10_(*p *value) = 1) corresponds to a 30% FDR for the Pearson's method.

**Figure 2:**
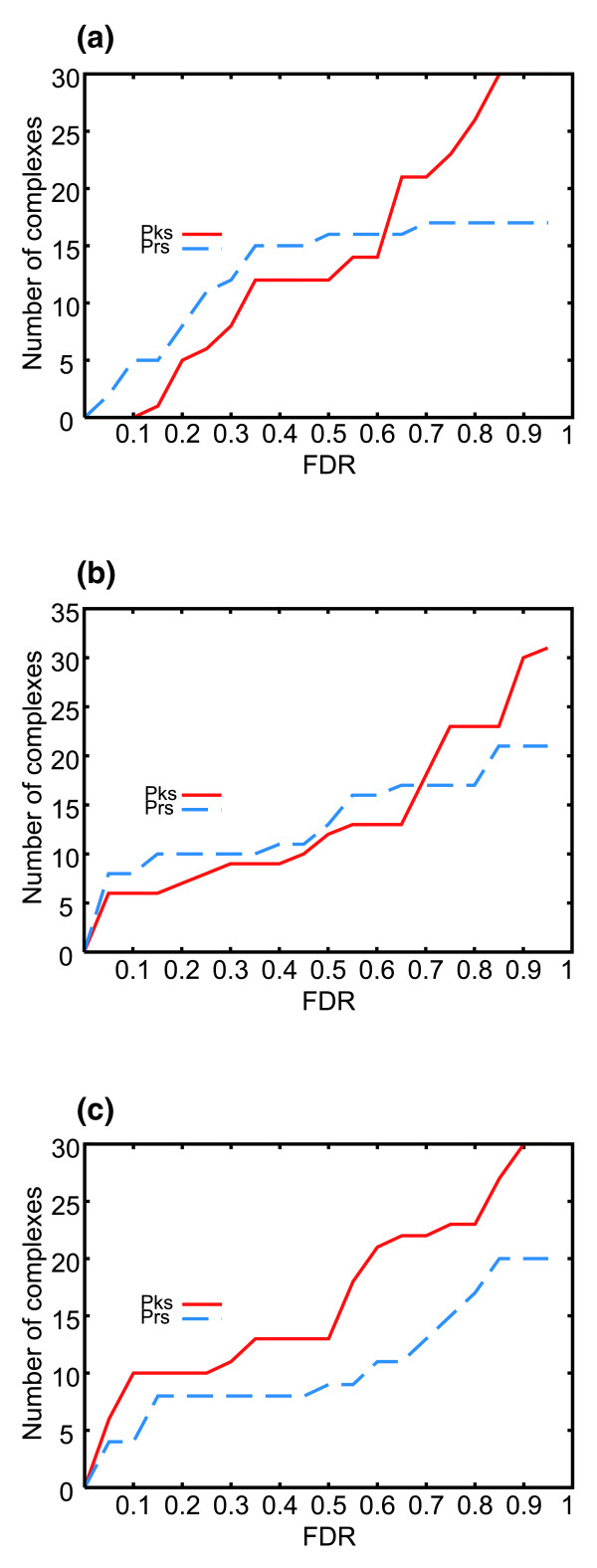
Comparison of sensitivity for the Pearson correlation coefficient (Prs) and expression peaks (Pks) methods. The number of complexes with best *p *value equal to or lower than the corresponding one on the x-axis is plotted for each HeLa cell-cycle dataset at a fixed FDR: **(a) **Thy-Thy2; **(b) **Thy-Thy3; **(c) **Thy-Noc.

Furthermore, we also compared the sensitivity of the Pearson and expression peaks methods (Figure 2). At a fixed FDR, the number of identified real complexes using either of the two methods was assessed. For the Thy-Thy datasets, with a low FDR, the Pearson's coefficient had a higher sensitivity in detecting high co-regulation among components of the same complex, while the expression peaks method clearly performed better across the different FDR ranges for the Thy-Noc dataset and at high FDRs for the Thy-Thy datasets.

**Figure 3:**
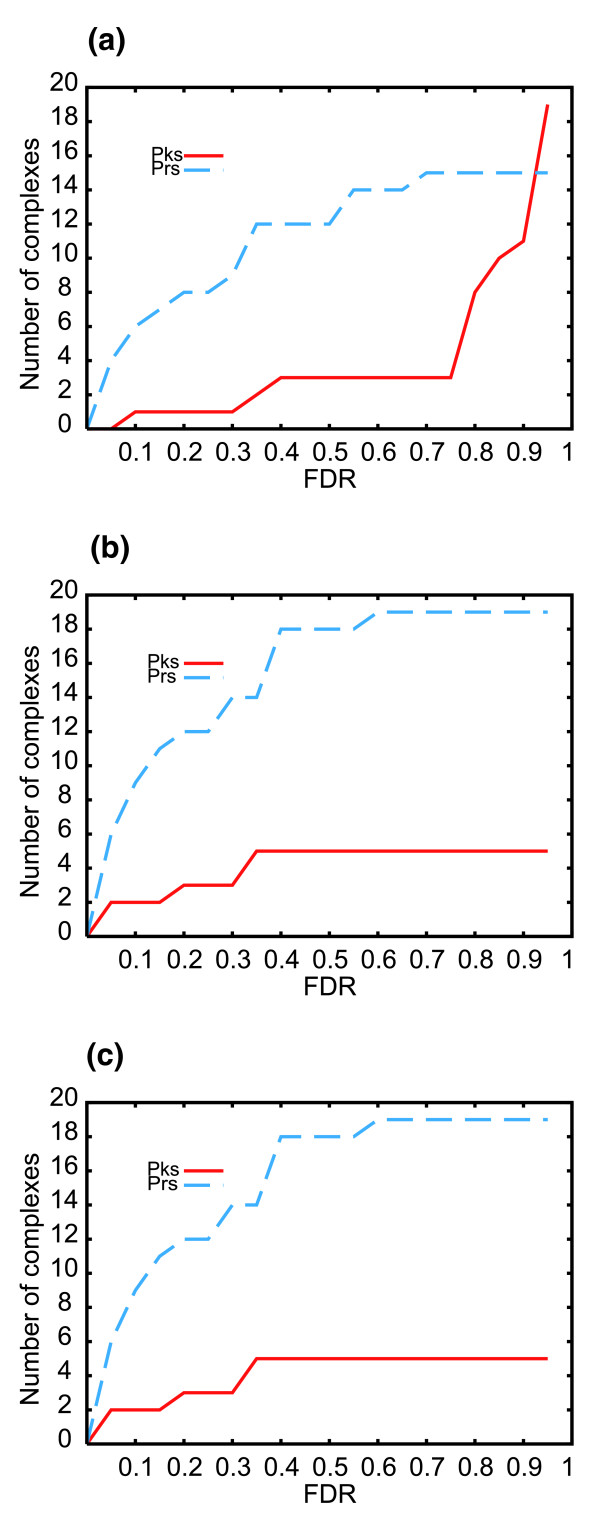
Non-synchronized HeLa cells. The number of complexes with a best *p *value equal to or lower than the corresponding one on the x-axis is plotted for three non-synchronized and stressed HeLa datasets at a fixed FDR: **(a) **dithiothreitol (DTT); **(b) **heat shock; **(c) **tunicamycin.

The Pearson's coefficient analysis and the expression peaks method were also used to study protein complexes in additional time series datasets analyzing non-synchronized HeLa cells subjected to several stresses [25]. Similar sensitivity for both synchronized and non-synchronized cells were obtained with the former method, while, as expected, the latter was more powerful in analyzing synchronized cells. Figure 3 and Additional data file 4 show the sensitivity of both methods for non-synchronized cells.

### PEGO: a web based computational tool that combines the expression peaks method with Gene Ontology annotations

To improve the ability of the expression peaks method to identify new putative interactors of given genes, our approach was combined with an extensive GO annotation analysis. We developed a web based tool named PEGO (Peaks Expression and Gene Ontology) [26] to provide public access to such an analysis. PEGO selects two groups of genes; the first contains all the genes that have the same expression peak pattern as the input genes while the second includes all the genes with the same GO categories as the input. The user can then intersect the two sets of genes to identify the putative interacting proteins in their input dataset. Moreover, the tool allows the output data to be restricted, such as selecting preferred GO annotation terms or isolating a given time point in the array experiment [19,25]. In Additional data files 5-7 results are shown that were obtained by querying PEGO with a list of genes from a subset of the analyzed human complexes.

### Generation of novel interaction candidates for PAK1

To test the predictive capability of our approach in detecting novel protein interactions, PAK1 was selected as candidate for study from the Thy-Noc dataset. PAK1 is a serine/threonine kinase implicated in the control of a number of cellular activities, including regulation of adhesive and trafficking processes, apoptosis, cell-cycle, and cytoskeletal dynamics [27,28]. We queried PEGO for PAK1 by using its ID [Entrez-Gene:5058], Organelle organization and biogenesis [GO:0006996] as the Biological process term and Cytoskeleton [GO:0005856] or Cytoplasm [GO:0005737] as the Cellular component term. According to this analysis, PAK1 was associated with three peaks. The highest percentage of genes with the same PAK1 GO annotation (Organelle organization and biogenesis) peaked in the time interval 14 h-12 h. Among them, 106 genes also displayed Cytoskeleton or Cytoplasm GO annotation (Additional data file 8); 5 of these genes are known interactors of PAK1 [29-33], 8 are similar to actin or actin-binding proteins, 4 are tubulins or tubulin-related proteins, 28 are proteins that localize also to the nucleus and 2 are involved in endocytosis. All these data largely match the known roles of PAK1, including the F-actin binding activity [28], the regulation of microtubule dynamics [34] and the involvement in cellular trafficking [28,35,36].

### Experimental validation

**Figure 4:**
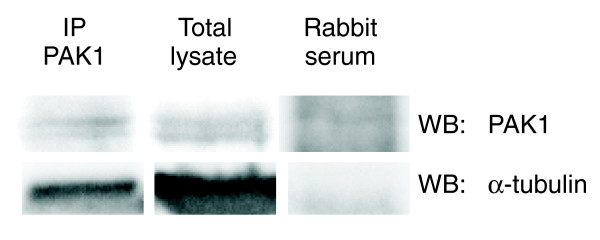
PAK1 physically interacts with α-tubulin. HeLa cell lysate was immunoprecipitated with anti-PAK1 antibody and blotted with anti-α-tubulin antibody. The figure is representative of three experiments obtained with similar results.

Using the described approach, α-tubulin and EEA1 were selected as new interacting partners of PAK1 to be experimentally validated in living mammalian cells. Using immunoprecipitation assays, we detected the physical interaction between endogenous PAK1 and α-tubulin in HeLa cells (Figure 4, and Additional data file 9).

**Figure 5:**
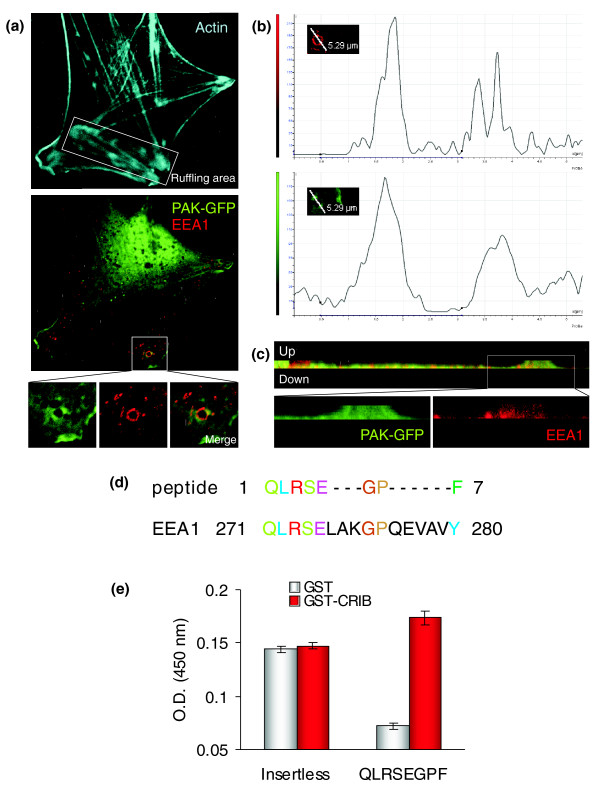
Experimental evidence for the interaction of PAK1 with EEA1. Confocal analysis of the cross section **(a) **and the vertical section **(c) **of PDGF-induced MEF cell reveals that endogenous EEA1 colocalized (yellow) with PAK1-GFP. **(b) **Quantification of the colocalization where the x-axis represents the white line in the inset (rotated -90° compared to (a)) and the y-axis represents the fluorescence intensity. The first peak of intensity in both channels indicates that PAK1 (green) and EEA1 (red) were enriched at the same site. **(d) **Sequence matching (computed with the multiple sequence alignment program ClustalW) obtained for the phage-display selected peptide QLRSEGPF and the aminoacidic sequence of EEA1. **(e) **Binding of the selected peptide (QLRSEGPF) to GST-CRIB and the negative control performed with GST alone. Binding of the insertless phage was tested with either GST or GST-CRIB, which showed no differences in affinity. The y-axis represents the absorbance (OD 450 nm). Results are the mean of triplicate experiments.

It is known that both PAK1 and EEA1 are involved in growth factor stimulated [36,37] macropinocytosis [38] and that PAK1 localizes to ruffling F-actin areas where macropinosomes form [28,39,40]. Therefore, to investigate the interaction between PAK1 and EEA1, murine embryo fibroblasts (MEFs) were stimulated with platelet-derived growth factor (PDGF) to produce F-actin ruffles [41,42]. Because there are no suitable antibodies for PAK-1 immunofluorescence, the MEFs were transfected with PAK-green fluorescent protein (GFP). Figure 5a-c shows the colocalization of PAK-GFP with endogenous EEA1 in vesicle-like structures located in ruffling areas. A similar pattern was observed also in MEFs transfected with PAK1-mRFP (data not shown) to exclude any non-specific effect the fluorescent tag may have on the colocalization.

To further demonstrate the direct interaction between PAK1 and EEA1, we screened a phage displayed peptide library with the Cdc42/Rac interactive binding (CRIB) domain of PAK1 fused to the glutathione S-transferase (GST), in the presence of glutathione-derivatized sepharose beads. An increase in phage binding over the negative control (GST/glutathione beads) was observed after three rounds of selection. DNA sequencing revealed the presence of a peptide insert corresponding to amino acids 271-280 of EEA1 (Figure 5d). The specificity of this peptide was confirmed by ELISA, where its binding affinity was tested on GST-CRIB purified protein compared to GST protein alone. Figure 5e shows that the selected peptide had a specific affinity for GST-CRIB, supporting the physical association between PAK1 and EEA1.

## Discussion

### Identification of protein complexes by *in silico *analysis of the expression profiles of human genes

In this work, we propose a new method to identify protein-protein interactions using gene expression data. The rationale behind our approach is the idea that a common transcriptional program drives the formation of both transient and permanent protein complexes in mammalian cells. It suggests that a selected gene expression dataset may contain useful information for *de novo *identification of protein interactions.

Because the decay rates of individual mRNAs range from 15 minutes to 24 hours [43], we focused our analysis on gene co-regulation in a single time interval to reduce noise. To asses the performance of our method with respect to the standard Pearson-based one we tested both of them with a set of 32 known complexes. To avoid problems due to multiple testing, we evaluated FDRs by comparing our results with those of thousands of randomly chosen sets of genes.

The main result of our analysis is that the study of synchronous peaks of expression can successfully complement the standard Pearson-based analysis of expression data. While Pearson-based methods are more effective in the identification of permanent interactions, our method is particularly suited for transient interactions. This observation suggests that it is best to use a combination of Pearson's and expression peak analyses for computational evaluation of protein complexes.

The higher sensitivity of the expression peaks method for transient complexes seems to be connected with its ability to detect quantitatively modest but functionally important changes in gene expression, which would otherwise be missed, especially in non-synchronized cell populations. With Pearson's analysis, a high statistical significance is obtained only for a small subset of complexes. In contrast, the expression peaks method gives statistically significant results for a greater number of complexes, although with higher *p *values than the Pearson's method.

Another important observation is that the expression peaks method performs better for well synchronized datasets (that is, the Thy-Noc treatment). On the basis of our methodological assumptions (that is, the half-life of mRNA [43]), this is not surprising as it restricts the application of this method to highly selected datasets. However, the current technical efforts to improve cell synchronization will extend the reliability of the expression peaks method to a larger number of gene expression datasets.

### Improvement of the expression peaks method by Gene Ontology analysis

The analysis of co-regulation during only a single time point increases the sensitivity of the expression peaks method, but also increases the noise. We therefore combined this method with GO analysis and found that this association reduced the number of false positives generated by the exclusive use of the expression peaks method. Combining both analyses reduced the number of potential candidate interactors to a few dozen while the output lists obtained by using either one of these approaches alone contained up to a thousand genes (Additional data file 6). A similar improvement was also recently observed by Corà *et al*. [44], who successfully combined GO and gene expression analyses in HeLa cell-cycle datasets to extract putative co-regulated genes for the identification of candidate transcription factor binding sites.

It is worthwhile to note that in several cases not all genes of the same complex were strictly co-regulated (Table 3, and Additional data file 3). This represents an intrinsic limitation of any approach based on gene expression data to identify protein interaction. Of course, subcellular localization, and post-transcriptional and post-translational modifications also play a key role in the assembly of both permanent and transient complexes [45-48]. Thus, the addition of further information, such as post-translational modifications, could greatly improve the quality of the results, an approach we plan to use in the future.

### PEGO public software

To enable researchers to test our computational approach, we implemented our pipeline as a web-based, publicly available tool named PEGO [26], which we have queried to identify new protein interactions that we validated experimentally. It is interesting to observe that the PEGO outputs contained additional interacting partners whose genes were not included in our query list. For instance, in the case of the dynactin complex (Additional data file 6c) five new candidates emerged, and two of these, that is, non-erythrocytic spectrins, turned out to be previously characterized interactors of the dynactin complex [49,50] (data not shown). While this result confirms the capability of our method to detect functional units, PEGO may actually be applied to a broader class of data types, in particular, to groups of genes without any known and obvious relationship. For example, one could analyze a list of genes that, if silenced, produce the same phenotype and use PEGO to detect any interactions among those candidates. Thus, unlike starting with a list of genes that have similar GO annotation, this approach excludes any prior bias for detection of protein-protein interactions. However, after a list of potential interactors has been generated, further GO analysis will increase the likelihood of detecting new complexes.

### Discovery of new interactors of PAK1 by combining PEGO with 'wet' biological experiments

The potential of PEGO has been confirmed by 'wet' biological experiments testing the *in silico *results obtained by submitting PAK1, as a single gene. We selected PAK1 due to our interest in cytoskeleton dynamics in vascular cells. PAK1 relates best to the GO biological process 'Organelle organization and biogenesis', because this category includes both cytoskeleton- and vesicular-related functions that fit well with the subcellular localization of PAK1 in living cells (data not shown). Among the three expression peaks of PAK1 in the Thy-Noc dataset, we selected the 14 h-12 h one because a high percentage of 'Organelle organization and biogenesis' annotated genes peaked there, thus suggesting a novel intriguing role of PAK1 in this process.

PEGO indicated a list of genes to be considered as potential new interactors of PAK1. In light of the PAK1-related literature, we evaluated α-tubulin and EEA1 as potential interactors to be experimentally confirmed. Previous work showed a co-localization of microtubules and PAK1 [51] and identified tubulin cofactor B (a cofactor associating with α- and β-tubulin) as an interacting substrate of PAK1 [34]. These data hint at an interaction between PAK1 and α-tubulin, although no experimental evidence has been obtained for this. We therefore used immunoprecipitation experiments in HeLa cells to demonstrate the physical interaction between PAK1 and α-tubulin supporting the immunofluorescence co-localization data previously reported [34].

The PAK1- EEA1 interaction, however, has not been reported before, and it represents a novel finding highlighting the potential of PEGO to predict unknown protein-protein interactions. Interestingly, we observed that PAK1 and EEA1 co-localize at sites resembling large vesicular structures. This hypothesis is supported by Dharmawardane *et al*. [36], who described the formation of large macropinocytic vesicles lined by PAK1 in PDGF-stimulated cells. Although the co-localization at these sites suggested a functional relationship between PAK1 and EEA1, the small amounts of overlayed proteins were not sufficient to test their physical interaction by immunoprecipitation. To overcome this technical problem, we screened a phage library to find specific peptides able to bind the CRIB domain of PAK1. Besides binding the small GTPases Cdc42 and Rac1, which trigger the catalytic activity of PAK1, the CRIB domain is also known to bind other transducers [22]. The selection of a peptide encompassing an amino acid region of EEA1 (Figure 5d) clearly showed that the observed co-localization in immunofluorescence studies between EEA1 and PAK1 indeed reflects a true interaction.

### Future perspectives

Our statistical approach to identify protein complexes could be improved by taking into account a greater number of microarray gene expression data obtained by *in vitro *experiments performed on specific models of cell activation. The same approach applied to *in vivo *animal models should also allow the discrimination of changes in a putative complex caused by the tissue microenvironment or during development. More interestingly, the comparison of microarray data obtained before and after silencing a specific gene by small RNA interference could allow the identification of new protein complexes and not just simply the identification of new interacting partners.

Finally, a further and relevant progression of our expression peaks method would be the inclusion of other information besides GO to reduce the number of false positives. This could include sequence analysis, evolutionary data or the use of the same experimental design to generate expression data from different animal species.

## Conclusion

We have presented a computational methodology to statistically analyze gene expression of several known human multi-protein complexes in a single time interval. With the obtained results we developed an approach to explore novel protein interactions by studying synchronously peaking genes with similar GO annotations from microarray datasets. By applying our method to PAK1, we found five previously known interactors, confirming the validity of our approach. Next, we validated the predicted interactions with two other proteins, α-tubulin and EEA1.

On the basis of these results, we would like to encourage researchers to use PEGO for their proteins of interest as an additional selection screen for the identification of potential interacting candidates to be experimentally validated.

## Materials and methods

### Microarray data

We studied HeLa cell time series [19,25] from the Stanford Microarray Database [24]. The analysis was performed for every dataset separately to evaluate gene expression strictly related to specific cellular conditions. Datasets were normalized such that the Euclidean norm of each expression profile was 1 and the average was 0. To evaluate changes in expression level at each step during the cell cycle, for each clone, we built a new expression vector containing differences of expression values computed between two consecutive time points. Since the HeLa cell-cycle datasets were published in 2002, we associated with all IMAGE clones new Entrez Gene IDs according to UniGene database version 183 [52], and we excluded from our analysis: clones with an ID different from the IMAGE ID or all numerical IDs; clones with more than one associated Entrez Gene ID; clones whose expression, measured as the log ratio between Cy5 (synchronous cells) and Cy3 (reference sample) channels, varied between -0.2 and +0.2.

### Human multi-protein complexes data set

The analyzed data set was composed of 32 permanent and transient protein complexes, in which interactions among different components are or are not maintained, respectively [14]. Complexes were selected from the following sources: the KEGG database [53] for SRS, large ribosomal subunit (LRS), proteasome, anaphase promoting complex, von hippel-lindau complex, SCF, signal recognition particle, RNA polymerase II, RNA polymerase III, and TAFIID; NCBI [54] for trafficking protein particle complex, nucleopore, mitochondrial small ribosomal subunit, MLRS, adaptor-related protein complex 2, origin recognition complex, pyruvate dehydrogenase, ATP synthase, H+ transporting, mitochondrial F0 complex, ATP synthase, H+ transporting, mitochondrial F1 complex, and SNARE complex; literature for nucleosome [19], focal adhesion [55], centrosome [56], dynactin [57], Arp2/3 [58], exosome [59], exocyst [60], axin-related complex (ARC) [61], SWI/SNF [62], cytochrome c oxidase [63], RFC [64], and golgi transport complex [65]. To each protein the corresponding Entrez Gene ID was assigned according to UniGene database, version 183 [52] (Additional data files 10 and 11).

### Analysis of multi-protein complexes by Pearson coefficient

We evaluated the gene expression correlation between clone ID pairs of genes using the Pearson Coefficient, *r*:

$$r=\frac{\sum_{i=1}^{n}\left(x_{i}-\bar{x}\right)\left(y_{i}-\bar{y}\right)}{\sqrt{\sum_{i=1}^{n}{\left(x_{i}-\bar{x}\right)}^{2}\sum_{i=1}^{n}{\left(y_{i}-\bar{y}\right)}^{2}}}$$

where (*x*_1_...*x*_*n*_) is the expression profile vector of clone *x*, (*y*_1_...y_*n*_) is the expression profile vector of clone *y*, *n *is the number of time points in the analyzed dataset:

$$\bar{x}=\frac{1}{n}\sum_{i=1}^{n}x_{i},\bar{y}=\frac{1}{n}\sum_{i=1}^{n}y_{i}.$$

and *r *is the normalized scalar product of two vectors with its value being in the range [-1, +1].

From the cumulative distribution of all the Pearson coefficients, we extracted the pairs of 'highly correlated' clones setting a threshold, *P*_*cutoff*_, having at its right 1% of the distribution. To define the number of highly correlated genes in each protein complex, we counted pairs containing both clone IDs corresponding to genes (according to their Entrez Gene ID) of the same complex. Pairs that appeared more than one time were counted only once and pairs containing single gene information (both clone IDs with the same Entrez Gene ID) were excluded. The probability that the number of gene pairs for each complex was recovered by chance was evaluated using the hypergeometric distribution:

$$P_{c}=\sum_{x=f_{c}}^{M}\frac{\left(\begin{array}{l} M\\ x \end{array}\right)\left(\begin{array}{l} N-M\\ n_{c}-x \end{array}\right)}{\left(\begin{array}{l} N\\ n_{c} \end{array}\right)}$$

where the index *c *runs over all the complexes we studied, *N *is the number of all gene pairs in the dataset, *M *is the number of genes pairs with *r *above *P*_*cutoff*_, *n*_*c *_is the number of all gene pairs in the analyzed complex, *c*, *f*_*c *_is the number of gene pairs in complex *c*, with *r *above 1% *P*_*cutoff*_.

To evaluate if a different threshold of the Pearson coefficient could be useful to extract a larger and statistically significant number of highly correlated gene pairs for protein complexes, we performed Pearson analysis as described above with different *P*_*cutoff*_. We found essentially no dependence of results on *P*_*cutoff *_and, therefore, used the standard value of 1% of the right tail of the Pearson coefficient distribution as threshold (data not shown).

### False discovery rate for Pearson coefficient analysis

We estimated a FDR for each *p *value, *P*_*c*_, by estimating the probability that the result obtained is a false positive. We performed 3,000 randomization cycles. For each cycle we generated a set composed of the same number of genes per complex as those analyzed, but randomly selected from the dataset. For each random set we calculated its *p *values, *p*, as described above. Then, we counted the number of real complexes, *n*(*p"*), or random complexes, *r*(*p"*) with a *p *value better than *p"*. In the random case we computed the average number over all the randomizations. For each *p" *we defined the FDR as:

$$FDR(p'')=\frac{r\left(p''\right)}{n\left(p''\right)}$$

Complexes composed of less than three components (that is, with a number of genes lower than three in the analyzed dataset) were not considered.

### Definition of expression peak

We defined a threshold on computed differences of gene expression between consecutive time points, indicating the value over which the increase of gene expression has to be considered an expression peak. Since we worked on datasets with time intervals of different lengths (one hour for the Thy-Thy datasets or two hours for the Thy-Noc dataset) and number, for each dataset separately, we analyzed the distribution of all computed differences and we selected that value that has at its right 20% of the distribution.

According to different stoichiometric features of protein complexes, the transcriptional machinery may produce different amounts of specific transcripts. Actually, the stoichiometric ratios of most complexes are still unknown [66,67]. To tackle this problem by a computational method, we changed all expression values to a binary 1-0 system [68]. Therefore, a new dataset matrix was generated where we substituted differences with '1' if corresponding to a peak of expression, or with '0' otherwise.

In order to test the dependence of our results on the 20% threshold, we performed expression peaks analysis on protein complexes using different thresholds and then compared the corresponding distributions of best *p *values. The results turned out to be largely independent from the chosen threshold, thus showing that our results are not biased by our threshold choice (data not shown).

### Definition of expression peak at gene level

To apply our method of analysis to a single gene of interest using the PEGO tool, for each gene a single pattern of peaks along the cell cycle was assigned, grouping data for clones annotated to the same Entrez Gene ID. First, we defined the maximum number of peaks, *m*, that could be associated with each gene by assuming that each clone has a single significant peak of expression, and we then evaluated *m *as the mean number of clones per gene considering all genes in the dataset. For each gene, in every interval, we summed the differences in expression levels of all its clones to obtain a single value and we selected only the *m *higher sums. By computing the sum, the noise due to the discrepancy among values of clones of the same gene was eliminated. Finally, to rebuild the 1-0 matrix, we transformed each of the *m *sums into '1', peak of expression, if it was large enough compared to the distribution of all differences in expression level computed for clones in the dataset (that is, if the value falls in the 20% tail of the distribution). All other values were replaced with '0'.

### Analysis of multi-protein complexes by the expression peaks method

To define synchronous peaks of expression, for each complex, *c*, in each interval, *i*, we counted the number of peaking genes, *f*_*ic*_. Then we evaluated the probability, *P*_*ic*_, to obtain *f*_*ic *_by chance through the hypergeometric distribution:

$$P_{ic}=\sum_{x=f_{ic}}^{Mi}\frac{\left(\begin{array}{l} M_{i}\\ x \end{array}\right)\left(\begin{array}{l} N-M_{i}\\ n_{c}-x \end{array}\right)}{\left(\begin{array}{l} N\\ n_{c} \end{array}\right)}$$

where *N *is the number of genes in the analyzed dataset, *M*_*i *_is the number of genes in the analyzed dataset that peak in a selected interval *I*, *n*_*c *_is the number of genes of a selected protein complex *c *in the analyzed dataset, and *f*_*ic *_is the number of genes of the selected protein complex *c *that peak in *i*. *P*_*ic *_represents the probability to obtain by chance at least *f*_*ic *_peaking genes, selecting randomly in the dataset a number of genes equal to that of the analyzed protein complex.

In order to compare (Figure 1) this definition of *p *value with that introduced in the previous section for the Pearson's method, the present *p *value has to be corrected for multiple testing. The standard Bonferroni procedure was performed by multiplying for the number of tested intervals (seven in the Thy-Noc dataset).

### False discovery rate for the expression peaks method

In order to estimate the FDR, we calculated for each random complex (generated as described for the Pearson method) the *p *value as discussed in the previous section and we counted the number of complexes, both in the real, *n*(*p"*), and in the random, *r*(*p"*), cases, that had a time interval with the best *p *value better than *p"*. For random complexes, we considered the average number *r*(*p"*) computed over all randomizations. For each *p" *we defined the FDR according to equation 3.

### Immunoprecipitation assay

Quiescent HeLa cells were harvested from plates by addition of 500 μl ice-cold lysis buffer containing 1% Triton X-100/100 mm dish. The cell lysates were pre-cleared with pre-immune rabbit serum and 50% (v/v) protein G-Sepharose (Amersham Biosciences, Piscataway, NJ, USA) for 2 h at 4°C and were then incubated with 50% (v/v) protein G-Sepharose and anti-PAK1 (rabbit polyclonal; Cell Signaling Technology, Beverly, MA, USA) for 2 h at 4°C. The immunoprecipitates were recovered and washed three times with lysis buffer. The washed immunoprecipitates were resuspended in 25 μl 2× Laemli sample buffer and analyzed by SDS-PAGE and western blotting using anti-α-tubulin (mouse monoclonal, clone B-5-1-2, Sigma) and anti-PAK1.

### Confocal analysis

MEFs were transfected (Fugene, Roche, Basel, Switzerland) with either PAK1-GFP (a gift from Dr G Bokoch, The Scripps Research Institute, La Jolla, California) or PAK1 tagged with the monomeric red fluorescent protein, PAK1-mRFP, (Additional data file 12). Twenty-four hours after transfection, cells were incubated in DMEM 0.5 % fetal bovine serum for 7 hours and then stimulated with PDGF for 6 minutes. Subsequently, cells were fixed with 3.7% para-formaldheyde and permeabilized with 0.01% saponin (Sigma, St. Louis, MO, USA). Cells were incubated with goat anti-EEA1 antibody (Santa Cruz, Biotechnology, Santa Cruz, CA, USA) and then with rabbit anti-goat Alexa 555 (Invitrogen Molecular Probes, Carlsbad, CA, USA). F-actin was stained by phalloidin Alexa 633 (Invitrogen, Carlsbad, CA, USA). Images were acquired with a Leica DMIRE2 confocal microscope and the analysis was performed with Leica Confocal software.

### Phage-display analysis

We pre-adsorbed 10^10 ^transducing units (TU) of a CX_7_C (C, cysteine; X, any amino acid residue) phage display random library on GST in the presence of glutathione-sepahrose beads in Iscove's Modified Dulbecco's Medium, IMDM, 2% fetal calf serum (binding medium), for 1 hour at room temperature. The pre-cleared phage library was then incubated with GST-fused PAK1 CRIB domain [69] in the presence of glutathione-sepharose beads in binding medium for 1 hour at room temperature. After five washes, bound phage were recovered and amplified by infection of exponentially growing K91Kan *Escherichia coli*. Serial dilutions were plated on LB agar plates with tetracycline and kanamycin. The numbers of TU were determined by bacterial colony counting [70,71].

### Peptide analysis and validation

After three rounds of selection, 20 phage clones were selected from each experiment, and the displayed peptides were deduced by sequencing the exogenous oligonucleotide inserts. For sequencing, we used the following primer: 5'-CCCTCATAGTTAGCGTAACG-3'. Sequence homologies were evaluated by searching non-redundant human protein databases (Additional data file 12).

The binding specificity of the selected peptides was evaluated by ELISA. Wells of a 96-well plate were coated with 1 μg of either GST or CRIB-GST in phosphate-buffered saline. We incubated 10^8 ^TU of each clone per well in binding medium for 1 hour at room temperature. After ten washes in the same medium, bound phage was stained with an anti-M13 antibody (anti-M13 bacteriophage; Sigma), detected by a secondary anti-mouse horseradish peroxidase-conjugated monoclonal antibody and quantified using the 1-Step Turbo TMB-ELISA kit (Pierce, Rockford, IL, USA).

## Abbreviations

CRIB, Cdc42/Rac interactive binding; GO, gene ontology; EEA, early endosome antigen; FDR, false discovery rate; GFP, green fluorescent protein; GST, glutathione S-transferase; LRS, large ribosomal subunit; MEF, murine embryo fibroblast; MLRS, mitochondrial large ribosomal subunit; PAK, p21-activated kinase; PDGF, platelet-derived growth factor; PEGO, Peaks Expression and Gene Ontology; RFC, replication complex; SCF, skp1-cull-F-box complex; SRS, small ribosomal subunit; Thy-Noc, thymidine-nocodazole synchronized cell dataset; Thy-Thy2, thymidine-thymidine synchronized cell dataset 2; Thy-Thy3, thymidine-thymidine synchronized cell dataset 3; TU, transducing units.

## Authors' contributions

SZ conceived the study, designed and coordinated it, performed PAK1-related analysis and experiments and drafted the manuscript. IC conceived the study, was involved in its initial coordination and design, performed phage-display peptide analysis and helped to draft the manuscript. CP performed the statistical analysis. IM performed the statistical analysis and developed the web based PEGO. SM performed phage-display experiments and helped to draft the manuscript. MC participated in the design of the project and coordination of the statistical analysis and helped to draft the manuscript. FB participated in the design of the project and coordination of the biological part and helped to draft the manuscript. All authors read and approved the final manuscript.

## Additional data files

The following additional data are available with the online version of this paper. Additional data file 1 is a table listing *p *values obtained from analyzing the Thy-Thy datasets with the expression peaks method or with Pearson correlation coefficient throughout the cell-cycle. Additional data file 2 is a table listing the FDR for each protein complex. Additional data file 3 is a table listing the percentages of synchronously peaking genes in the Thy-Thy2 and Thy-Thy3 datasets. Additional data file 4 is a plot representing the number of complexes with a best *p *value equal to or lower than the corresponding one on the x-axis for three non-synchronized and stressed HeLa datasets at a fixed FDR. Additional data file 5 is a table listing GO term and time interval with best *p *value for a subset of the human protein complexes analyzed with PEGO. Additional data file 6 is a table listing the number of recovered components for a subset of the human protein complexes analyzed with PEGO. Additional data file 7 is a table listing the GO analysis results for a subset of the human protein complexes analyzed with PEGO. Additional data file 8 is a table listing the selected interaction candidates for PAK1. Additional data file 9 is an image showing the specificity of anti-PAK1 and anti α-tubulin antibodies. Additional data file 10 is a table listing Entrez Gene IDs of all components of each protein complex analyzed. Additional data file 11 is a table listing the IMAGE IDs for each component of the analyzed protein complexes. Additional data file 12 contains supplemental materials and methods.

## Supplementary Material

Additional data file 1 *P *values obtained with the expression peaks method in each time interval of the cell-cycle or with Pearson correlation coefficient throughout the cell-cycle (Cell-cycle column). **(a) **Thy-Thy2; **(b) **Thy-Thy3. Click here for file

Additional data file 2 **(a, d) **Thy-Thy2 dataset; **(b, e) **Thy-Thy3 dataset; **(c, f) **Thy-Noc dataset. ND, not defined. Click here for file

Additional data file 3 For each protein complex, the percentage of its synchronously peaking genes in each time interval is reported. **(a) **Thy-Thy2; **(b) **Thy-Thy3. Click here for file

Additional data file 4 The number of complexes with a best *p *value equal to or lower than the corresponding one on the x-axis for three non-synchronized and stressed HeLa datasets at a fixed FDR. **(a) **Crowding; **(b) **H_2_O_2_; **(c) **menadione. Click here for file

Additional data file 5 A subset of the human protein complexes was analyzed with PEGO. For each protein complex, the GO Biological process term(s), G, and the time interval, T, if present, are indicated, displaying best *p *value lower than 0.05 after statistical analysis by means of hypergeometric distribution (Table 2, and Additional data files 1 and 7). These are the input parameters used to obtain Additional data file 6. **(a) **Thy-Thy2; **(b) **Thy-Thy3; **(c) **Thy-Noc. An asterisk indicates that the *p *value for the expression peak was between 0.1 and 0.05. Click here for file

Additional data file 6 Time interval (T) with best *p *value and GO Biological process term (G) with best *p *value were used to query PEGO (Additional data file 5). T genes, number of genes in the dataset with expression peak in T; G genes, number of genes in the dataset annotated at G; I genes, number of genes in the dataset with expression peak in T and GO G; T complex genes, number of genes of the protein complex with expression peak in T; G complex genes, number of genes of the protein complex with GO G; I complex genes, number of genes of the protein complex with expression peak in T and GO G; T % complex genes, (T complex genes/T genes) × 100; G % complex genes, (G complex genes/G genes) × 100; I % complex genes, I complex genes/I genes) × 100; % complex genes, percentage of genes of the protein complex with expression peak in T and GO G. An asterisk indicates that the *p *value for the expression peak is between 0.1 and 0.05. **(a) **Thy-Thy2; **(b) **Thy-Thy3; **(c) **Thy-Noc. Click here for file

Additional data file 7 The first column lists the complexes (the same as analyzed in Additional data file 5), and the second, third and fourth columns provide information on the ontology and GO category. In the following four columns we report the input information for *p *value evaluation using the hypergeometric distribution (Additional data file 12) and in the last column the corresponding *p *values. Only GO terms with a *p *value with exponent lower or equal to E-05 are reported. **(a) **Thy-Thy2; **(b) **Thy-Thy3; **(c) **Thy-Noc. Click here for file

Additional data file 8 Text: List of genes with an expression peak at the 14 h-12 h interval in the Thy-Noc dataset and annotated as Organelle organization and biogenesis [GO:0006996] for the Biological process term and as Cytoskeleton [GO:0005856] or Cytoplasm [GO:0005737] for the Cellular component term. Click here for file

Additional data file 9 Images show a broader molecular weight range of the blots in Figure 4. **(a) **HeLa cell lysate was immunoprecipitated with anti-PAK1 antibody or rabbit serum and blotted with anti α-tubulin antibody. **(b) **HeLa cell lysate blotted with anti-PAK1 antibody. The time exposure of (b) is higher than in Figure 4 to better evaluate the specificity of the antibody. Click here for file

Additional data file 10 Entrez Gene IDs of all components of each protein complex analyzed. Click here for file

Additional data file 11 For each gene, indicated by its Entrez Gene ID, the corresponding IMAGE IDs present in each dataset is reported. **(a) **Thy-Thy2; **(b) **Thy-Thy3; **(c) **Thy-Noc. Click here for file

Additional data file 12 Supplemental Materials and methods. Click here for file
