# Supplementary figures and images for: A new computational approach to analyze human protein complexes and predict novel protein interactions
Source: Genome Biol. 2007 Dec 4;8(12):R256. doi: 10.1186/gb-2007-8-12-r256 (PMC2246258; doi:10.1186/gb-2007-8-12-r256)

**A**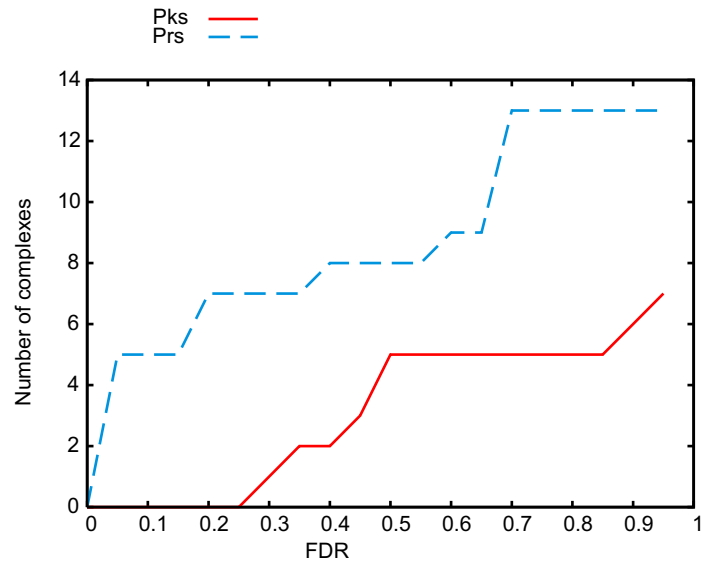**B**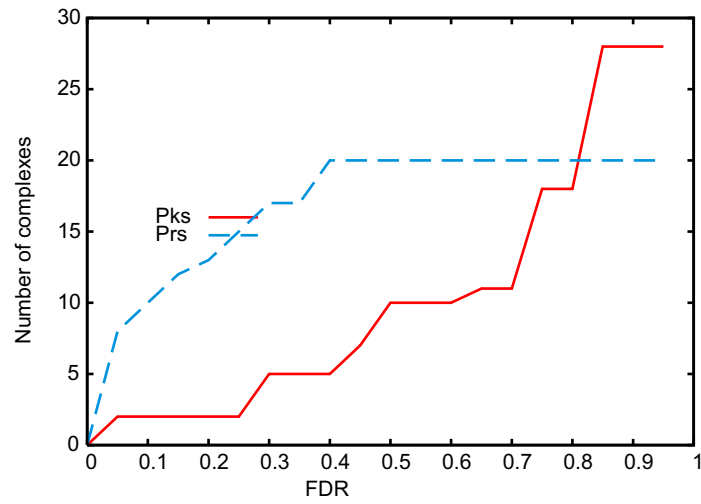**C**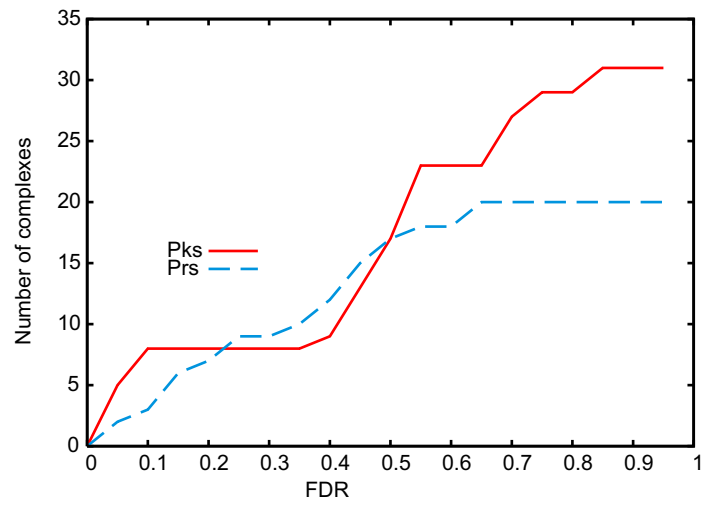

Supplement: Additional data file 4 — The number of complexes with a best p value equal to or lower than the corresponding one on the x-axis for three non-synchronized and stressed HeLa datasets at a fixed FDR. (a) Crowding; (b) H2O2; (c) menadione. [file gb-2007-8-12-r256-S4.pdf]

A

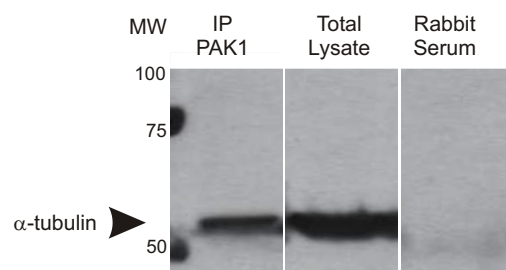

B

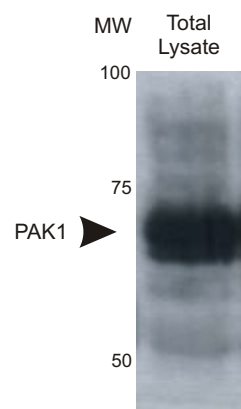

Supplement: Additional data file 9 — Images show a broader molecular weight range of the blots in Figure 4. (a) HeLa cell lysate was immunoprecipitated with anti-PAK1 antibody or rabbit serum and blotted with anti α-tubulin antibody. (b) HeLa cell lysate blotted with anti-PAK1 antibody. The time exposure of (b) is higher than in Figure 4 to better evaluate the specificity of the antibody. [file gb-2007-8-12-r256-S9.pdf]
