# Supplementary material for: A new computational approach to analyze human protein complexes and predict novel protein interactions
Source: Genome Biol. 2007 Dec 4;8(12):R256. doi: 10.1186/gb-2007-8-12-r256 (PMC2246258; doi:10.1186/gb-2007-8-12-r256)
Supplement: Additional data file 6 — Time interval (T) with best p value and GO Biological process term (G) with best p value were used to query PEGO (Additional data file 5). T genes, number of genes in the dataset with expression peak in T; G genes, number of genes in the dataset annotated at G; I genes, number of genes in the dataset with expression peak in T and GO G; T complex genes, number of genes of the protein complex with expression peak in T; G complex genes, number of genes of the protein complex with GO G; I complex genes, number of genes of the protein complex with expression peak in T and GO G; T % complex genes, (T complex genes/T genes) × 100; G % complex genes, (G complex genes/G genes) × 100; I % complex genes, I complex genes/I genes) × 100; % complex genes, percentage of genes of the protein complex with expression peak in T and GO G. An asterisk indicates that the p value for the expression peak is between 0.1 and 0.05. (a) Thy-Thy2; (b) Thy-Thy3; (c) Thy-Noc. [file gb-2007-8-12-r256-S6.pdf]

A

Protein complex	T genes	G genes	I genes	T complex genes	G complex genes	I complex genes	T % complex genes	G % complex genes	I % complex genes	% complex genes
Arp2-3	1818	16	7	5	4	4	0.28	25.00	57.14	66.67
APC	703	136	10	2	5	2	0.28	3.68	20.00	25.00
Dynactin	1493	22	4	3	2	2	0.20	9.09	50.00	22.22
Exocyst	1100	60	4	3	7	3	0.27	11.67	75.00	42.86
Exosome	703	41	3	2	3	2	0.28	7.32	66.67	40.00
FA	1658	795	81	12	14	3	0.72	1.76	3.70	6.12
LRS	1818	666	68	6	15	6	0.33	2.25	8.82	33.33
Nucleosome	599	123	3	3	17	3	0.50	13.82	100.00	11.11
Proteasome	1818	645	77	8	18	5	0.44	2.79	6.49	20.83
RNA Pol II	1100	2035	150	4	10	4	0.36	0.49	2.67	36.36
SCF	1139	565	50	2	2	2	0.18	0.35	4.00	66.67
SRS	1818	666	68	7	14	5	0.39	2.10	7.35	22.73
TAFIID	1139	57	6	3	6	1	0.26	10.53	16.67	7.69

B

Protein complex	T genes	G genes	I genes	T complex genes	G complex genes	I complex genes	T % complex genes	G % complex genes	I % complex genes	% complex genes
APC	2302	136	25	5	8	5	0.22	5.88	20.00	62.50
Dynactin	2300	22	4	4	4	0	0.17	18.18	0.00	0.00
Exocyst	2448	60	14	4	7	4	0.16	11.67	28.57	57.14
FA*	2302	3586	391	12	26	8	0.52	0.73	2.05	16.33
LRS	2003	666	58	7	18	7	0.35	2.70	12.07	38.89
Nucleosome	3270	166	33	13	24	13	0.40	14.46	39.39	48.15
Proteasome	3270	134	35	13	13	9	0.40	9.70	25.71	37.50
RNA Pol II	3270	2035	322	6	10	6	0.18	0.49	1.86	54.55
RNA Pol III	2003	25	2	3	3	1	0.15	12.00	50.00	14.29
SRP*	3270	11	3	3	3	2	0.09	27.27	66.67	40.00
SRS	2930	666	99	14	18	12	0.48	2.70	12.12	54.55
TAFIID	2302	57	12	5	8	2	0.22	14.04	16.67	15.38

C

Protein complex	T genes	G genes	I genes	T complex genes	G complex genes	I complex genes	T % complex genes	G % complex genes	I % complex genes	% complex genes
APC	3819	136	56	5	8	5	0.13	5.88	8.93	62.5
Centrosome	1570	152	9	18	12	1	1.15	7.89	11.11	1.85
Dynactin	3819	22	7	5	3	2	0.13	13.64	28.57	22.22
Exocyst	2203	60	11	4	7	4	0.18	11.67	36.36	57.14
FA*	3819	3586	584	18	27	6	0.47	0.75	1.03	12.24
LRS	3819	666	153	11	18	11	0.29	2.70	7.19	61.11
Nucleosome	3437	166	19	10	24	10	0.29	14.46	52.63	37.04
Proteasome	3819	645	129	18	19	15	0.47	2.95	11.63	62.5
RNA Pol II	3058	2035	327	5	10	5	0.16	0.49	1.53	45.45
RNA Pol III	2520	25	3	3	3	1	0.12	12.00	33.33	14.29
SRP*	3819	11	3	3	2	1	0.08	18.18	33.33	20
SCF	3819	565	143	3	3	3	0.08	0.53	2.10	100
SRS	3819	666	153	11	18	10	0.29	2.70	6.54	45.45
