# Supplementary material for: A new computational approach to analyze human protein complexes and predict novel protein interactions
Source: Genome Biol. 2007 Dec 4;8(12):R256. doi: 10.1186/gb-2007-8-12-r256 (PMC2246258; doi:10.1186/gb-2007-8-12-r256)
Supplement: Additional data file 7 — The first column lists the complexes (the same as analyzed in Additional data file 5), and the second, third and fourth columns provide information on the ontology and GO category. In the following four columns we report the input information for p value evaluation using the hypergeometric distribution (Additional data file 12) and in the last column the corresponding p values. Only GO terms with a p value with exponent lower or equal to E-05 are reported. (a) Thy-Thy2; (b) Thy-Thy3; (c) Thy-Noc. [file gb-2007-8-12-r256-S7.pdf]

A

Protein complex	Ontology type	GO Term	GO ID	N	M	n	m	P-value
Arp2-3	molecular function	structural molecule activity	GO:0005198	6268	326	6	5	2.12E-06
Arp2-3	molecular function	structural constituent of cytoskeleton	GO:0005200	6268	56	6	3	1.33E-05
Arp2-3	cellular component	Arp2/3 protein complex	GO:0005885	5334	4	6	4	4.45E-13
Arp2-3	cellular component	cytoskeleton	GO:0005856	5334	395	6	6	1.59E-07
Arp2-3	cellular component	non-membrane-bound organelle	GO:0043228	5334	647	6	6	3.12E-06
Arp2-3	cellular component	intracellular non-membrane-bound organelle	GO:0043232	5334	647	6	6	3.12E-06
Arp2-3	cellular component	actin cytoskeleton	GO:0015629	5334	121	6	4	3.65E-06
Arp2-3	biological process	actin filament polymerization	GO:0030041	5847	13	5	4	7.34E-11
Arp2-3	biological process	protein polymerization	GO:0051258	5847	26	5	4	1.53E-09
Arp2-3	biological process	actin polymerization and/or depolymerization	GO:0008154	5847	31	5	4	3.22E-09
Arp2-3	biological process	regulation of actin filament polymerization	GO:0030833	5847	6	5	3	6.00E-09
Arp2-3	biological process	actin cytoskeleton organization and biogenesis	GO:0030036	5847	76	5	4	1.31E-07
Arp2-3	biological process	actin filament-based process	GO:0030029	5847	84	5	4	1.96E-07
Arp2-3	biological process	regulation of actin polymerization and/or depolymerization	GO:0008064	5847	22	5	3	4.60E-07
Arp2-3	biological process	regulation of actin filament length	GO:0030832	5847	22	5	3	4.60E-07
Arp2-3	biological process	cell motility	GO:0006928	5847	117	5	4	7.50E-07
Arp2-3	biological process	regulation of cell organization and biogenesis	GO:0051128	5847	30	5	3	1.21E-06
Arp2-3	biological process	regulation of protein metabolism	GO:0051246	5847	53	5	3	6.95E-06
Arp2-3	biological process	cytoskeleton organization and biogenesis	GO:0007010	5847	208	5	4	7.57E-06
APC	cellular component	nuclear ubiquitin ligase complex	GO:0000152	5334	6	4	3	3.16E-09
APC	cellular component	anaphase-promoting complex	GO:0005680	5334	6	4	3	3.16E-09
APC	biological process	mitosis	GO:0007067	5847	87	5	5	6.50E-10
APC	biological process	M phase of mitotic cell cycle	GO:0000087	5847	89	5	5	7.30E-10
APC	biological process	M phase	GO:0000279	5847	108	5	5	1.96E-09
APC	biological process	mitotic cell cycle	GO:0000278	5847	135	5	5	6.10E-09
APC	biological process	cell division	GO:0051301	5847	95	5	4	3.23E-07
APC	biological process	cell cycle	GO:0007049	5847	405	5	5	1.56E-06
APC	biological process	mitotic metaphase/anaphase transition	GO:0007091	5847	3	5	2	1.75E-06
APC	biological process	regulation of cell cycle	GO:0000074	5847	265	5	4	1.99E-05
APC	biological process	ubiquitin cycle	GO:0006512	5847	324	5	4	4.43E-05
Centrosome	molecular function	motor activity	GO:0003774	6268	76	32	6	1.84E-06
Centrosome	cellular component	microtubule cytoskeleton	GO:0015630	5334	130	29	20	1.02E-26
Centrosome	cellular component	cytoskeleton	GO:0005856	5334	395	29	22	7.45E-20
Centrosome	cellular component	microtubule organizing center	GO:0005815	5334	34	29	11	3.74E-18
Centrosome	cellular component	spindle	GO:0005819	5334	44	29	11	9.71E-17
Centrosome	cellular component	microtubule	GO:0005874	5334	91	29	13	2.33E-16
Centrosome	cellular component	centrosome	GO:0005813	5334	32	29	10	2.36E-16
Centrosome	cellular component	spindle pole	GO:0000922	5334	36	29	10	9.18E-16
Centrosome	cellular component	non-membrane-bound organelle	GO:0043228	5334	647	29	22	3.45E-15

Protein complex	Ontology type	GO Term	GO ID	N	M	n	m	P-value
Centrosome	cellular component	intracellular non-membrane-bound organelle	GO:0043232	5334	647	29	22	3.45E-15
Centrosome	cellular component	dynein complex	GO:0030286	5334	12	29	5	2.55E-09
Centrosome	cellular component	microtubule associated complex	GO:0005875	5334	52	29	7	7.30E-09
Centrosome	cellular component	cAMP-dependent protein kinase complex	GO:0005952	5334	5	29	3	1.43E-06
Centrosome	cellular component	spindle microtubule	GO:0005876	5334	7	29	3	4.99E-06
Centrosome	cellular component	centriole	GO:0005814	5334	3	29	2	8.53E-05
Centrosome	cellular component	polar microtubule	GO:0005827	5334	3	29	2	8.53E-05
Centrosome	biological process	microtubule-based process	GO:0007017	5847	85	26	11	2.04E-14
Centrosome	biological process	mitosis	GO:0007067	5847	87	26	10	1.37E-12
Centrosome	biological process	M phase of mitotic cell cycle	GO:0000087	5847	89	26	10	1.73E-12
Centrosome	biological process	mitotic cell cycle	GO:0000278	5847	135	26	11	3.80E-12
Centrosome	biological process	M phase	GO:0000279	5847	108	26	10	1.26E-11
Centrosome	biological process	cytoskeleton organization and biogenesis	GO:0007010	5847	208	26	12	1.87E-11
Centrosome	biological process	organelle organization and biogenesis	GO:0006996	5847	446	26	14	7.64E-10
Centrosome	biological process	microtubule cytoskeleton organization and biogenesis	GO:0000226	5847	35	26	6	6.20E-09
Centrosome	biological process	cell organization and biogenesis	GO:0016043	5847	547	26	14	1.12E-08
Centrosome	biological process	cell cycle	GO:0007049	5847	405	26	12	4.12E-08
Centrosome	biological process	microtubule-based movement	GO:0007018	5847	47	26	5	1.56E-06
Centrosome	biological process	cytoskeleton-dependent intracellular transport	GO:0030705	5847	47	26	5	1.56E-06
Centrosome	biological process	microtubule nucleation	GO:0007020	5847	7	26	3	2.70E-06
Centrosome	biological process	protein polymerization	GO:0051258	5847	26	26	4	4.30E-06
Centrosome	biological process	cell division	GO:0051301	5847	95	26	5	5.11E-05
Dynactin	molecular function	motor activity	GO:0003774	6268	76	6	3	3.34E-05
Dynactin	cellular component	cytoskeleton	GO:0005856	5334	395	7	7	1.16E-08
Dynactin	cellular component	actin cytoskeleton	GO:0015629	5334	121	7	5	1.12E-07
Dynactin	cellular component	non-membrane-bound organelle	GO:0043228	5334	647	7	7	3.75E-07
Dynactin	cellular component	intracellular non-membrane-bound organelle	GO:0043232	5334	647	7	7	3.75E-07
Dynactin	cellular component	dynactin complex	GO:0005869	5334	2	7	2	1.48E-06
Dynactin	cellular component	F-actin capping protein complex	GO:0008290	5334	5	7	2	1.47E-05
Dynactin	cellular component	microtubule associated complex	GO:0005875	5334	52	7	3	2.98E-05
Dynactin	cellular component	dynein complex	GO:0030286	5334	12	7	2	9.68E-05
Dynactin	biological process	actin filament depolymerization	GO:0030042	5847	14	5	2	5.30E-05
Dynactin	biological process	regulation of actin filament depolymerization	GO:0030834	5847	14	5	2	5.30E-05
Dynactin	biological process	negative regulation of actin filament depolymerization	GO:0030835	5847	14	5	2	5.30E-05
Dynactin	biological process	barbed-end actin filament capping	GO:0051016	5847	14	5	2	5.30E-05
Dynactin	biological process	negative regulation of cell organization and biogenesis	GO:0051129	5847	19	5	2	9.95E-05
Dynactin	biological process	protein depolymerization	GO:0051261	5847	19	5	2	9.95E-05
Exocyst	cellular component	exocyst	GO:0000145	5334	5	6	5	1.67E-16
Exocyst	cellular component	cell cortex	GO:0005938	5334	16	6	5	7.28E-13
Exocyst	biological process	exocytosis	GO:0006887	5847	40	7	7	4.04E-16

Protein complex	Ontology type	GO Term	GO ID	N	M	n	m	P-value
Exocyst	biological process	secretory pathway	GO:0045045	5847	96	7	7	2.58E-13
Exocyst	biological process	secretion	GO:0046903	5847	121	7	7	1.37E-12
Exocyst	biological process	vesicle-mediated transport	GO:0016192	5847	197	7	7	4.44E-11
Exocyst	biological process	vesicle docking	GO:0048278	5847	16	7	4	1.30E-09
Exocyst	biological process	protein transport	GO:0015031	5847	331	7	7	1.75E-09
Exocyst	biological process	establishment of protein localization	GO:0045184	5847	332	7	7	1.79E-09
Exocyst	biological process	protein localization	GO:0008104	5847	343	7	7	2.26E-09
Exocyst	biological process	vesicle docking during exocytosis	GO:0006904	5847	15	7	3	4.75E-07
Exocyst	biological process	transport	GO:0006810	5847	1086	7	7	7.51E-06
Exocyst	biological process	establishment of localization	GO:0051234	5847	1087	7	7	7.55E-06
Exocyst	biological process	localization	GO:0051179	5847	1096	7	7	8.01E-06
Exosome	molecular function	3'-5' exonuclease activity	GO:0008408	6268	14	3	3	8.87E-09
Exosome	molecular function	exonuclease activity	GO:0004527	6268	21	3	3	3.24E-08
Exosome	molecular function	nuclease activity	GO:0004518	6268	58	3	3	7.52E-07
Exosome	molecular function	3'-5'-exoribonuclease activity	GO:0000175	6268	4	3	2	9.16E-07
Exosome	molecular function	exoribonuclease activity	GO:0004532	6268	5	3	2	1.53E-06
Exosome	molecular function	exoribonuclease activity. producing 5'-phosphomonoesters	GO:0016896	6268	5	3	2	1.53E-06
Exosome	molecular function	exonuclease activity. active with either ribo- or deoxyribonucleic acids and producing 5'-phosphomonoesters	GO:0016796	6268	6	3	2	2.29E-06
Exosome	molecular function	ribonuclease activity	GO:0004540	6268	23	3	2	3.86E-05
Exosome	cellular component	exosome (RNase complex)	GO:0000178	5334	3	3	2	6.33E-07
Exosome	biological process	rRNA processing	GO:0006364	5847	23	3	3	5.32E-08
Exosome	biological process	rRNA metabolism	GO:0016072	5847	27	3	3	8.78E-08
Exosome	biological process	ribosome biogenesis	GO:0007046	5847	37	3	3	2.33E-07
Exosome	biological process	ribosome biogenesis and assembly	GO:0042254	5847	41	3	3	3.20E-07
Exosome	biological process	cytoplasm organization and biogenesis	GO:0007028	5847	50	3	3	5.89E-07
Exosome	biological process	RNA processing	GO:0006396	5847	208	3	3	4.44E-05
Exosome	biological process	RNA catabolism	GO:0006401	5847	24	3	2	4.83E-05
Exosome	biological process	RNA metabolism	GO:0016070	5847	251	3	3	7.82E-05
FA	molecular function	phorbol ester receptor activity	GO:0001565	6268	10	32	5	6.19E-10
FA	molecular function	protein kinase C activity	GO:0004697	6268	10	32	5	6.19E-10
FA	molecular function	protein serine/threonine kinase activity	GO:0004674	6268	269	32	11	4.29E-08
FA	molecular function	cytoskeletal protein binding	GO:0008092	6268	172	32	9	1.17E-07
FA	molecular function	protein kinase activity	GO:0004672	6268	336	32	11	4.17E-07
FA	molecular function	diacylglycerol binding	GO:0019992	6268	35	32	5	7.29E-07
FA	molecular function	phosphotransferase activity. alcohol group as acceptor	GO:0016773	6268	394	32	11	2.04E-06
FA	molecular function	kinase activity	GO:0016301	6268	459	32	11	9.05E-06
FA	molecular function	actin binding	GO:0003779	6268	115	32	6	2.05E-05
FA	molecular function	protein-tyrosine kinase activity	GO:0004713	6268	176	32	7	2.27E-05
FA	molecular function	transferase activity. transferring phosphorus-containing groups	GO:0016772	6268	524	32	11	3.19E-05
FA	cellular component	actin cytoskeleton	GO:0015629	5334	121	24	7	6.53E-07
FA	cellular component	caveolar membrane	GO:0016599	5334	5	24	3	7.96E-07

Protein complex	Ontology type	GO Term	GO ID	N	M	n	m	P-value
FA	cellular component	caveola	GO:0005901	5334	6	24	3	1.59E-06
FA	cellular component	cytoskeleton	GO:0005856	5334	395	24	10	3.38E-06
FA	cellular component	plasma membrane	GO:0005886	5334	793	24	12	5.16E-05
FA	biological process	phosphorus metabolism	GO:0006793	5847	479	28	14	7.00E-09
FA	biological process	phosphate metabolism	GO:0006796	5847	479	28	14	7.00E-09
FA	biological process	protein amino acid phosphorylation	GO:0006468	5847	325	28	11	1.21E-07
FA	biological process	intracellular signaling cascade	GO:0007242	5847	574	28	13	6.42E-07
FA	biological process	phosphorylation	GO:0016310	5847	384	28	11	6.61E-07
FA	biological process	cell communication	GO:0007154	5847	1597	28	20	1.41E-06
FA	biological process	signal transduction	GO:0007165	5847	1282	28	18	1.66E-06
LRS	molecular function	structural constituent of ribosome	GO:0003735	6268	93	15	15	1.15E-28
LRS	molecular function	structural molecule activity	GO:0005198	6268	326	15	15	4.04E-20
LRS	molecular function	RNA binding	GO:0003723	6268	338	15	8	3.04E-07
LRS	molecular function	rRNA binding	GO:0019843	6268	4	15	2	3.20E-05
LRS	cellular component	ribosome	GO:0005840	5334	83	15	15	2.02E-28
LRS	cellular component	ribonucleoprotein complex	GO:0030529	5334	186	15	15	7.83E-23
LRS	cellular component	large ribosomal subunit	GO:0015934	5334	15	15	8	2.54E-18
LRS	cellular component	cytosolic large ribosomal subunit (sensu Eukaryota)	GO:0005842	5334	9	15	7	9.52E-18
LRS	cellular component	cytosolic ribosome (sensu Eukaryota)	GO:0005830	5334	18	15	7	8.31E-15
LRS	cellular component	non-membrane-bound organelle	GO:0043228	5334	647	15	15	1.57E-14
LRS	cellular component	intracellular non-membrane-bound organelle	GO:0043232	5334	647	15	15	1.57E-14
LRS	cellular component	protein complex	GO:0043234	5334	916	15	15	3.03E-12
LRS	cellular component	cytoplasm	GO:0005737	5334	1723	15	15	4.17E-08
LRS	cellular component	cytosol	GO:0005829	5334	175	15	7	1.87E-07
LRS	biological process	protein biosynthesis	GO:0006412	5847	280	15	15	1.11E-20
LRS	biological process	macromolecule biosynthesis	GO:0009059	5847	316	15	15	7.12E-20
LRS	biological process	cellular biosynthesis	GO:0044249	5847	501	15	15	8.12E-17
LRS	biological process	biosynthesis	GO:0009058	5847	527	15	15	1.75E-16
LRS	biological process	cellular protein metabolism	GO:0044267	5847	1624	15	15	4.31E-09
LRS	biological process	protein metabolism	GO:0019538	5847	1634	15	15	4.73E-09
LRS	biological process	cellular macromolecule metabolism	GO:0044260	5847	1745	15	15	1.27E-08
LRS	biological process	macromolecule metabolism	GO:0043170	5847	1821	15	15	2.42E-08
Nucleosome	molecular function	DNA binding	GO:0003677	6268	949	17	17	1.02E-14
Nucleosome	molecular function	nucleic acid binding	GO:0003676	6268	1503	17	17	2.67E-11
Nucleosome	cellular component	nucleosome	GO:0000786	5334	27	17	17	1.34E-42
Nucleosome	cellular component	chromatin	GO:0000785	5334	68	17	17	7.16E-34
Nucleosome	cellular component	chromosome	GO:0005694	5334	132	17	17	1.71E-28
Nucleosome	cellular component	non-membrane-bound organelle	GO:0043228	5334	647	17	17	2.21E-16
Nucleosome	cellular component	intracellular non-membrane-bound organelle	GO:0043232	5334	647	17	17	2.21E-16
Nucleosome	cellular component	protein complex	GO:0043234	5334	916	17	17	8.68E-14
Nucleosome	cellular component	nucleus	GO:0005634	5334	1878	17	17	1.87E-08
Nucleosome	cellular component	membrane-bound organelle	GO:0043227	5334	2752	17	17	1.27E-05
Nucleosome	cellular component	intracellular membrane-bound organelle	GO:0043231	5334	2752	17	17	1.27E-05
Nucleosome	biological process	nucleosome assembly	GO:0006334	5847	38	17	17	9.60E-40

Protein complex	Ontology type	GO Term	GO ID	N	M	n	m	P-value
Nucleosome	biological process	chromatin assembly or disassembly	GO:0006333	5847	65	17	17	6.23E-35
Nucleosome	biological process	establishment and/or maintenance of chromatin architecture	GO:0006325	5847	124	17	17	1.15E-29
Nucleosome	biological process	DNA packaging	GO:0006323	5847	127	17	17	1.78E-29
Nucleosome	biological process	chromosome organization and biogenesis (sensu Eukaryota)	GO:0007001	5847	148	17	17	2.83E-28
Nucleosome	biological process	protein complex assembly	GO:0006461	5847	149	17	17	3.20E-28
Nucleosome	biological process	chromosome organization and biogenesis	GO:0051276	5847	153	17	17	5.14E-28
Nucleosome	biological process	DNA metabolism	GO:0006259	5847	314	17	17	1.69E-22
Nucleosome	biological process	organelle organization and biogenesis	GO:0006996	5847	446	17	17	7.53E-20
Nucleosome	biological process	cell organization and biogenesis	GO:0016043	5847	547	17	17	2.56E-18
Nucleosome	biological process	biopolymer metabolism	GO:0043283	5847	908	17	17	1.56E-14
Nucleosome	biological process	nucleobase. nucleoside. nucleotide and nucleic acid metabolism	GO:0006139	5847	1588	17	17	2.24E-10
Nucleosome	biological process	cellular protein metabolism	GO:0044267	5847	1624	17	17	3.28E-10
Nucleosome	biological process	protein metabolism	GO:0019538	5847	1634	17	17	3.64E-10
Nucleosome	biological process	cellular macromolecule metabolism	GO:0044260	5847	1745	17	17	1.12E-09
Nucleosome	biological process	macromolecule metabolism	GO:0043170	5847	1821	17	17	2.32E-09
Proteasome	molecular function	threonine endopeptidase activity	GO:0004298	6268	18	19	13	6.33E-32
Proteasome	molecular function	endopeptidase activity	GO:0004175	6268	193	19	13	3.45E-16
Proteasome	molecular function	peptidase activity	GO:0008233	6268	282	19	13	5.03E-14
Proteasome	molecular function	hydrolase activity	GO:0016787	6268	1010	19	17	3.67E-12
Proteasome	molecular function	catalytic activity	GO:0003824	6268	2601	19	17	1.98E-05
Proteasome	cellular component	proteasome complex (sensu Eukaryota)	GO:0000502	5334	33	21	20	0.00E+00
Proteasome	cellular component	cytosol	GO:0005829	5334	175	21	21	2.04E-32
Proteasome	cellular component	proteasome core complex (sensu Eukaryota)	GO:0005839	5334	18	21	13	3.87E-30
Proteasome	cellular component	endoplasmic reticulum	GO:0005783	5334	288	21	20	4.68E-25
Proteasome	cellular component	protein complex	GO:0043234	5334	916	21	21	7.06E-17
Proteasome	cellular component	cytoplasm	GO:0005737	5334	1723	21	21	4.55E-11
Proteasome	cellular component	nucleus	GO:0005634	5334	1878	21	21	2.80E-10
Proteasome	cellular component	membrane-bound organelle	GO:0043227	5334	2752	21	21	8.88E-07
Proteasome	cellular component	intracellular membrane-bound organelle	GO:0043231	5334	2752	21	21	8.88E-07
Proteasome	cellular component	organelle	GO:0043226	5334	3110	21	21	1.17E-05
Proteasome	cellular component	intracellular organelle	GO:0043229	5334	3110	21	21	1.17E-05
Proteasome	cellular component	proteasome regulatory particle (sensu Eukaryota)	GO:0005838	5334	4	21	2	8.82E-05
Proteasome	biological process	protein catabolism	GO:0030163	5847	324	18	18	1.54E-23
Proteasome	biological process	biopolymer catabolism	GO:0043285	5847	330	18	18	2.16E-23
Proteasome	biological process	macromolecule catabolism	GO:0009057	5847	396	18	18	6.23E-22
Proteasome	biological process	proteolysis and peptidolysis	GO:0006508	5847	318	18	17	3.61E-21
Proteasome	biological process	cellular protein catabolism	GO:0044257	5847	318	18	17	3.61E-21
Proteasome	biological process	ubiquitin-dependent protein catabolism	GO:0006511	5847	86	18	13	4.76E-21
Proteasome	biological process	modification-dependent protein catabolism	GO:0019941	5847	86	18	13	4.76E-21

Protein complex	Ontology type	GO Term	GO ID	N	M	n	m	P-value
Proteasome	biological process	catabolism	GO:0009056	5847	502	18	18	4.85E-20
Proteasome	biological process	cellular macromolecule catabolism	GO:0044265	5847	390	18	17	1.24E-19
Proteasome	biological process	cellular catabolism	GO:0044248	5847	472	18	17	3.35E-18
Proteasome	biological process	biopolymer metabolism	GO:0043283	5847	908	18	18	2.39E-15
Proteasome	biological process	protein metabolism	GO:0019538	5847	1634	18	18	1.01E-10
Proteasome	biological process	macromolecule metabolism	GO:0043170	5847	1821	18	18	7.17E-10
Proteasome	biological process	cellular protein metabolism	GO:0044267	5847	1624	18	17	4.37E-09
Proteasome	biological process	cellular macromolecule metabolism	GO:0044260	5847	1745	18	17	1.45E-08
Proteasome	biological process	primary metabolism	GO:0044238	5847	3420	18	18	6.30E-05
RNA Pol II	molecular function	DNA-directed RNA polymerase activity	GO:0003899	6268	24	10	10	7.66E-26
RNA Pol II	molecular function	nucleotidyltransferase activity	GO:0016779	6268	61	10	10	3.52E-21
RNA Pol II	molecular function	transferase activity. transferring phosphorus-containing groups	GO:0016772	6268	524	10	10	1.54E-11
RNA Pol II	molecular function	transferase activity	GO:0016740	6268	830	10	10	1.58E-09
RNA Pol II	molecular function	DNA binding	GO:0003677	6268	949	10	8	9.10E-06
RNA Pol II	molecular function	nucleic acid binding	GO:0003676	6268	1503	10	9	2.02E-05
RNA Pol II	cellular component	RNA polymerase complex	GO:0030880	5334	19	10	8	2.10E-19
RNA Pol II	cellular component	DNA-directed RNA polymerase II. core complex	GO:0005665	5334	14	10	7	1.69E-17
RNA Pol II	cellular component	DNA-directed RNA polymerase II. holoenzyme	GO:0016591	5334	48	10	7	3.57E-13
RNA Pol II	cellular component	nucleoplasm	GO:0005654	5334	154	10	7	1.63E-09
RNA Pol II	cellular component	protein complex	GO:0043234	5334	916	10	8	2.39E-05
RNA Pol II	cellular component	nucleus	GO:0005634	5334	1878	10	10	2.88E-05
RNA Pol II	biological process	transcription	GO:0006350	5847	1021	10	10	2.54E-08
RNA Pol II	biological process	transcription from Pol II promoter	GO:0006366	5847	304	10	7	1.01E-07
RNA Pol II	biological process	nucleobase. nucleoside. nucleotide and nucleic acid metabolism	GO:0006139	5847	1588	10	10	2.14E-06
RNA Pol II	biological process	transcription. DNA-dependent	GO:0006351	5847	950	10	8	1.56E-05
RNA Pol III	molecular function	DNA-directed RNA polymerase activity	GO:0003899	6268	24	4	4	1.65E-10
RNA Pol III	molecular function	nucleotidyltransferase activity	GO:0016779	6268	61	4	4	8.12E-09
RNA Pol III	molecular function	transferase activity. transferring phosphorus-containing groups	GO:0016772	6268	524	4	4	4.83E-05
RNA Pol III	cellular component	RNA polymerase complex	GO:0030880	5334	19	4	4	1.15E-10
RNA Pol III	cellular component	DNA-directed RNA polymerase III complex	GO:0005666	5334	4	4	3	6.33E-10
RNA Pol III	cellular component	nucleoplasm	GO:0005654	5334	154	4	3	9.24E-05
RNA Pol III	biological process	transcription from Pol III promoter	GO:0006383	5847	15	4	2	3.68E-05
SRP	cellular component	signal recognition particle (sensu Eukaryota)	GO:0005786	5334	7	3	3	1.38E-09
SRP	cellular component	signal recognition particle	GO:0048500	5334	7	3	3	1.38E-09
SRP	cellular component	ribonucleoprotein complex	GO:0030529	5334	186	3	3	4.17E-05
SRS	molecular function	structural constituent of ribosome	GO:0003735	6268	93	16	16	1.43E-30
SRS	molecular function	structural molecule activity	GO:0005198	6268	326	16	16	2.01E-21
SRS	cellular component	ribosome	GO:0005840	5334	83	16	14	1.82E-24

Protein complex	Ontology type	GO Term	GO ID	N	M	n	m	P-value
SRS	cellular component	ribonucleoprotein complex	GO:0030529	5334	186	16	16	2.52E-24
SRS	cellular component	small ribosomal subunit	GO:0015935	5334	23	16	7	1.13E-13
SRS	cellular component	protein complex	GO:0043234	5334	916	16	16	5.13E-13
SRS	cellular component	non-membrane-bound organelle	GO:0043228	5334	647	16	14	1.25E-11
SRS	cellular component	intracellular non-membrane-bound organelle	GO:0043232	5334	647	16	14	1.25E-11
SRS	cellular component	cytosolic small ribosomal subunit (sensu Eukaryota)	GO:0005843	5334	9	16	5	1.52E-11
SRS	cellular component	eukaryotic 48S initiation complex	GO:0016283	5334	9	16	5	1.52E-11
SRS	cellular component	cytosolic ribosome (sensu Eukaryota)	GO:0005830	5334	18	16	5	1.02E-09
SRS	cellular component	eukaryotic 43S preinitiation complex	GO:0016282	5334	19	16	5	1.38E-09
SRS	cellular component	cytoplasm	GO:0005737	5334	1723	16	14	7.65E-06
SRS	biological process	protein biosynthesis	GO:0006412	5847	280	14	14	2.43E-19
SRS	biological process	macromolecule biosynthesis	GO:0009059	5847	316	14	14	1.38E-18
SRS	biological process	cellular biosynthesis	GO:0044249	5847	501	14	14	9.72E-16
SRS	biological process	biosynthesis	GO:0009058	5847	527	14	14	1.99E-15
SRS	biological process	cellular protein metabolism	GO:0044267	5847	1624	14	14	1.56E-08
SRS	biological process	protein metabolism	GO:0019538	5847	1634	14	14	1.70E-08
SRS	biological process	cellular macromolecule metabolism	GO:0044260	5847	1745	14	14	4.29E-08
SRS	biological process	macromolecule metabolism	GO:0043170	5847	1821	14	14	7.80E-08
TAFIID	molecular function	RNA polymerase II transcription factor activity	GO:0003702	6268	130	8	7	1.11E-11
TAFIID	molecular function	general RNA polymerase II transcription factor activity	GO:0016251	6268	26	8	5	4.54E-11
TAFIID	molecular function	transcription regulator activity	GO:0030528	6268	650	8	7	9.12E-07
TAFIID	molecular function	DNA binding	GO:0003677	6268	949	8	7	1.24E-05
TAFIID	cellular component	transcription factor TFIID complex	GO:0005669	5334	11	8	8	1.02E-23
TAFIID	cellular component	DNA-directed RNA polymerase II. holoenzyme	GO:0016591	5334	48	8	8	2.33E-17
TAFIID	cellular component	transcription factor complex	GO:0005667	5334	58	8	8	1.19E-16
TAFIID	cellular component	nucleoplasm	GO:0005654	5334	154	8	8	4.03E-13
TAFIID	cellular component	protein complex	GO:0043234	5334	916	8	8	7.37E-07
TAFIID	biological process	transcription initiation	GO:0006352	5847	36	8	6	9.77E-13
TAFIID	biological process	transcription. DNA-dependent	GO:0006351	5847	950	8	8	4.74E-07
TAFIID	biological process	transcription	GO:0006350	5847	1021	8	8	8.45E-07
TAFIID	biological process	regulation of transcription. DNA-dependent	GO:0006355	5847	912	8	7	1.52E-05
TAFIID	biological process	regulation of transcription	GO:0045449	5847	970	8	7	2.33E-05
TAFIID	biological process	regulation of nucleobase. nucleoside. nucleotide and nucleic acid metabolism	GO:0019219	5847	989	8	7	2.66E-05
TAFIID	biological process	nucleobase. nucleoside. nucleotide and nucleic acid metabolism	GO:0006139	5847	1588	8	8	2.92E-05
TAFIID	biological process	regulation of metabolism	GO:0019222	5847	1081	8	7	4.88E-05
VHL	biological process	ubiquitin cycle	GO:0006512	5847	324	4	4	9.26E-06

B

Protein complex	Ontology type	GO Term	GO ID	N	M	n	m	P-value
Arp2-3	cellular component	Arp2/3 protein complex	GO:0005885	7542	3	3	3	1.40E-11
Arp2-3	cellular component	actin cytoskeleton	GO:0015629	7542	157	3	3	8.85E-06
Arp2-3	biological process	regulation of actin filament polymerization	GO:0030833	8259	6	3	2	1.32E-06
Arp2-3	biological process	actin filament polymerization	GO:0030041	8259	11	3	2	4.83E-06
Arp2-3	biological process	cell motility	GO:0006928	8259	150	3	3	5.87E-06
Arp2-3	biological process	protein polymerization	GO:0051258	8259	22	3	2	2.03E-05
Arp2-3	biological process	regulation of actin polymerization and/or depolymerization	GO:0008064	8259	27	3	2	3.08E-05
Arp2-3	biological process	regulation of actin filament length	GO:0030832	8259	27	3	2	3.08E-05
Arp2-3	biological process	actin polymerization and/or depolymerization	GO:0008154	8259	35	3	2	5.22E-05
Arp2-3	biological process	regulation of cell organization and biogenesis	GO:0051128	8259	39	3	2	6.50E-05
APC	molecular function	ubiquitin-protein ligase activity	GO:0004842	8974	296	7	4	3.75E-05
APC	molecular function	acid-amino acid ligase activity	GO:0016881	8974	312	7	4	4.62E-05
APC	molecular function	ligase activity. forming carbon-nitrogen bonds	GO:0016879	8974	337	7	4	6.25E-05
APC	cellular component	nuclear ubiquitin ligase complex	GO:0000152	7542	9	7	6	2.30E-18
APC	cellular component	anaphase-promoting complex	GO:0005680	7542	9	7	6	2.30E-18
APC	cellular component	ubiquitin ligase complex	GO:0000151	7542	254	7	6	9.37E-09
APC	biological process	mitosis	GO:0007067	8259	115	8	8	1.11E-15
APC	biological process	M phase of mitotic cell cycle	GO:0000087	8259	117	8	8	1.27E-15
APC	biological process	M phase	GO:0000279	8259	143	8	8	6.64E-15
APC	biological process	mitotic cell cycle	GO:0000278	8259	173	8	8	3.16E-14
APC	biological process	cell division	GO:0051301	8259	122	8	7	1.02E-12
APC	biological process	cell cycle	GO:0007049	8259	513	8	8	2.10E-10
APC	biological process	mitotic metaphase/anaphase transition	GO:0007091	8259	3	8	3	5.97E-10
APC	biological process	regulation of cell cycle	GO:0000074	8259	338	8	7	1.40E-09
APC	biological process	mitotic anaphase	GO:0000090	8259	5	8	3	5.96E-09
APC	biological process	anaphase	GO:0051322	8259	5	8	3	5.96E-09
APC	biological process	ubiquitin cycle	GO:0006512	8259	435	8	7	8.20E-09
APC	biological process	interphase	GO:0051325	8259	69	8	4	3.05E-07
APC	biological process	interphase of mitotic cell cycle	GO:0051329	8259	69	8	4	3.05E-07
APC	biological process	regulation of mitotic metaphase/anaphase transition	GO:0030071	8259	2	8	2	8.21E-07
APC	biological process	ubiquitin-dependent protein catabolism	GO:0006511	8259	99	8	4	1.31E-06
APC	biological process	modification-dependent protein catabolism	GO:0019941	8259	99	8	4	1.31E-06
APC	biological process	regulation of mitosis	GO:0007088	8259	31	8	3	2.65E-06
APC	biological process	G2/M transition of mitotic cell cycle	GO:0000086	8259	40	8	3	5.80E-06
APC	biological process	protein modification	GO:0006464	8259	1231	8	7	1.12E-05
Centrosome	molecular function	tubulin binding	GO:0015631	8974	30	32	5	5.56E-08
Centrosome	molecular function	motor activity	GO:0003774	8974	104	32	6	1.49E-06
Centrosome	molecular function	microtubule binding	GO:0008017	8974	21	32	3	5.24E-05
Centrosome	cellular component	microtubule cytoskeleton	GO:0015630	7542	177	31	23	5.19E-32
Centrosome	cellular component	microtubule organizing center	GO:0005815	7542	39	31	14	1.74E-25
Centrosome	cellular component	cytoskeleton	GO:0005856	7542	537	31	25	5.94E-24
Centrosome	cellular component	spindle	GO:0005819	7542	50	31	14	1.06E-23

Protein complex	Ontology type	GO Term	GO ID	N	M	n	m	P-value
Centrosome	cellular component	spindle pole	GO:0000922	7542	40	31	13	5.76E-23
Centrosome	cellular component	centrosome	GO:0005813	7542	35	31	12	1.59E-21
Centrosome	cellular component	microtubule	GO:0005874	7542	126	31	15	2.26E-19
Centrosome	cellular component	non-membrane-bound organelle	GO:0043228	7542	881	31	25	1.32E-18
Centrosome	cellular component	intracellular non-membrane-bound organelle	GO:0043232	7542	881	31	25	1.32E-18
Centrosome	cellular component	dynein complex	GO:0030286	7542	21	31	5	1.63E-08
Centrosome	cellular component	microtubule associated complex	GO:0005875	7542	76	31	7	1.73E-08
Centrosome	cellular component	gamma-tubulin complex	GO:0000930	7542	5	31	3	6.25E-07
Centrosome	cellular component	spindle microtubule	GO:0005876	7542	7	31	3	2.18E-06
Centrosome	cellular component	cAMP-dependent protein kinase complex	GO:0005952	7542	8	31	3	3.47E-06
Centrosome	cellular component	organelle	GO:0043226	7542	4197	31	28	3.24E-05
Centrosome	cellular component	intracellular organelle	GO:0043229	7542	4197	31	28	3.24E-05
Centrosome	cellular component	centriole	GO:0005814	7542	3	31	2	4.89E-05
Centrosome	cellular component	polar microtubule	GO:0005827	7542	3	31	2	4.89E-05
Centrosome	cellular component	intracellular	GO:0005622	7542	5078	31	30	7.40E-05
Centrosome	biological process	microtubule-based process	GO:0007017	8259	107	27	12	1.76E-16
Centrosome	biological process	cytoskeleton organization and biogenesis	GO:0007010	8259	256	27	13	2.46E-13
Centrosome	biological process	microtubule cytoskeleton organization and biogenesis	GO:0000226	8259	47	27	8	1.20E-12
Centrosome	biological process	mitosis	GO:0007067	8259	115	27	10	1.27E-12
Centrosome	biological process	M phase of mitotic cell cycle	GO:0000087	8259	117	27	10	1.52E-12
Centrosome	biological process	mitotic cell cycle	GO:0000278	8259	173	27	11	2.42E-12
Centrosome	biological process	M phase	GO:0000279	8259	143	27	10	1.16E-11
Centrosome	biological process	organelle organization and biogenesis	GO:0006996	8259	568	27	15	2.44E-11
Centrosome	biological process	microtubule nucleation	GO:0007020	8259	12	27	5	1.97E-10
Centrosome	biological process	cell organization and biogenesis	GO:0016043	8259	697	27	15	4.51E-10
Centrosome	biological process	cell cycle	GO:0007049	8259	513	27	12	2.14E-08
Centrosome	biological process	microtubule-based movement	GO:0007018	8259	56	27	4	2.96E-05
Centrosome	biological process	cytoskeleton-dependent intracellular transport	GO:0030705	8259	56	27	4	2.96E-05
Centrosome	biological process	cell division	GO:0051301	8259	122	27	5	4.03E-05
Centrosome	biological process	protein polymerization	GO:0051258	8259	22	27	3	4.60E-05
Dynactin	molecular function	actin binding	GO:0003779	8974	164	8	4	7.11E-06
Dynactin	molecular function	cytoskeletal protein binding	GO:0008092	8974	239	8	4	3.16E-05
Dynactin	molecular function	motor activity	GO:0003774	8974	104	8	3	8.12E-05
Dynactin	cellular component	cytoskeleton	GO:0005856	7542	537	9	9	4.42E-11
Dynactin	cellular component	actin cytoskeleton	GO:0015629	7542	157	9	7	5.16E-11
Dynactin	cellular component	F-actin capping protein complex	GO:0008290	7542	8	9	4	6.53E-11
Dynactin	cellular component	non-membrane-bound organelle	GO:0043228	7542	881	9	9	3.91E-09
Dynactin	cellular component	intracellular non-membrane-bound organelle	GO:0043232	7542	881	9	9	3.91E-09
Dynactin	cellular component	dynactin complex	GO:0005869	7542	3	9	2	3.80E-06
Dynactin	cellular component	microtubule associated complex	GO:0005875	7542	76	9	3	7.91E-05
Dynactin	cellular component	protein complex	GO:0043234	7542	1247	9	7	8.79E-05
Dynactin	biological process	actin filament depolymerization	GO:0030042	8259	19	7	4	6.97E-10

Protein complex	Ontology type	GO Term	GO ID	N	M	n	m	P-value
Dynactin	biological process	regulation of actin filament depolymerization	GO:0030834	8259	19	7	4	6.97E-10
Dynactin	biological process	negative regulation of actin filament depolymerization	GO:0030835	8259	19	7	4	6.97E-10
Dynactin	biological process	barbed-end actin filament capping	GO:0051016	8259	19	7	4	6.97E-10
Dynactin	biological process	regulation of actin polymerization and/or depolymerization	GO:0008064	8259	27	7	4	3.15E-09
Dynactin	biological process	regulation of actin filament length	GO:0030832	8259	27	7	4	3.15E-09
Dynactin	biological process	negative regulation of cell organization and biogenesis	GO:0051129	8259	27	7	4	3.15E-09
Dynactin	biological process	protein depolymerization	GO:0051261	8259	27	7	4	3.15E-09
Dynactin	biological process	actin polymerization and/or depolymerization	GO:0008154	8259	35	7	4	9.37E-09
Dynactin	biological process	regulation of cell organization and biogenesis	GO:0051128	8259	39	7	4	1.47E-08
Dynactin	biological process	negative regulation of protein metabolism	GO:0051248	8259	39	7	4	1.47E-08
Dynactin	biological process	regulation of protein metabolism	GO:0051246	8259	69	7	4	1.53E-07
Dynactin	biological process	actin cytoskeleton organization and biogenesis	GO:0030036	8259	92	7	4	4.92E-07
Dynactin	biological process	cytoskeleton organization and biogenesis	GO:0007010	8259	256	7	5	5.49E-07
Dynactin	biological process	actin filament-based process	GO:0030029	8259	99	7	4	6.62E-07
Dynactin	biological process	organelle organization and biogenesis	GO:0006996	8259	568	7	6	6.80E-07
Dynactin	biological process	cell organization and biogenesis	GO:0016043	8259	697	7	6	2.30E-06
Dynactin	biological process	negative regulation of metabolism	GO:0009892	8259	164	7	4	5.01E-06
Dynactin	biological process	negative regulation of cellular physiological process	GO:0051243	8259	247	7	4	2.55E-05
Dynactin	biological process	negative regulation of cellular process	GO:0048523	8259	292	7	4	4.93E-05
Exocyst	cellular component	exocyst	GO:0000145	7542	5	6	5	2.95E-17
Exocyst	cellular component	cell cortex	GO:0005938	7542	20	6	5	4.57E-13
Exocyst	biological process	exocytosis	GO:0006887	8259	49	7	7	1.66E-16
Exocyst	biological process	secretory pathway	GO:0045045	8259	118	7	7	1.02E-13
Exocyst	biological process	secretion	GO:0046903	8259	152	7	7	6.23E-13
Exocyst	biological process	vesicle-mediated transport	GO:0016192	8259	251	7	7	2.21E-11
Exocyst	biological process	vesicle docking	GO:0048278	8259	18	7	4	5.51E-10
Exocyst	biological process	protein transport	GO:0015031	8259	434	7	7	1.06E-09
Exocyst	biological process	establishment of protein localization	GO:0045184	8259	436	7	7	1.09E-09
Exocyst	biological process	protein localization	GO:0008104	8259	449	7	7	1.34E-09
Exocyst	biological process	vesicle docking during exocytosis	GO:0006904	8259	17	7	3	2.52E-07
Exocyst	biological process	transport	GO:0006810	8259	1557	7	7	8.37E-06
Exocyst	biological process	establishment of localization	GO:0051234	8259	1559	7	7	8.45E-06
Exocyst	biological process	localization	GO:0051179	8259	1571	7	7	8.91E-06
Exosome	molecular function	exonuclease activity	GO:0004527	8974	42	5	5	1.76E-12
Exosome	molecular function	nuclease activity	GO:0004518	8974	94	5	5	1.13E-10
Exosome	molecular function	3'-5' exonuclease activity	GO:0008408	8974	23	5	4	1.64E-10
Exosome	molecular function	3'-5'-exoribonuclease activity	GO:0000175	8974	9	5	3	6.97E-09

Protein complex	Ontology type	GO Term	GO ID	N	M	n	m	P-value
Exosome	molecular function	exoribonuclease activity	GO:0004532	8974	11	5	3	1.37E-08
Exosome	molecular function	exoribonuclease activity. producing 5'-phosphomonoesters	GO:0016896	8974	11	5	3	1.37E-08
Exosome	molecular function	exonuclease activity. active with either ribo- or deoxyribonucleic acids and producing 5'-phosphomonoesters	GO:0016796	8974	13	5	3	2.37E-08
Exosome	molecular function	RNA binding	GO:0003723	8974	412	5	5	1.99E-07
Exosome	molecular function	hydrolase activity. acting on ester bonds	GO:0016788	8974	444	5	5	2.90E-07
Exosome	molecular function	ribonuclease activity	GO:0004540	8974	38	5	3	6.97E-07
Exosome	molecular function	hydrolase activity	GO:0016787	8974	1384	5	5	8.67E-05
Exosome	cellular component	exosome (RNase complex)	GO:0000178	7542	6	5	4	5.57E-13
Exosome	cellular component	nucleolus	GO:0005730	7542	69	5	4	3.19E-08
Exosome	biological process	rRNA processing	GO:0006364	8259	29	5	5	3.71E-13
Exosome	biological process	rRNA metabolism	GO:0016072	8259	33	5	5	7.42E-13
Exosome	biological process	ribosome biogenesis	GO:0007046	8259	44	5	5	3.40E-12
Exosome	biological process	ribosome biogenesis and assembly	GO:0042254	8259	49	5	5	5.96E-12
Exosome	biological process	cytoplasm organization and biogenesis	GO:0007028	8259	61	5	5	1.86E-11
Exosome	biological process	RNA processing	GO:0006396	8259	261	5	5	3.04E-08
Exosome	biological process	RNA metabolism	GO:0016070	8259	332	5	5	1.02E-07
Exosome	biological process	organelle organization and biogenesis	GO:0006996	8259	568	5	5	1.51E-06
Exosome	biological process	cell organization and biogenesis	GO:0016043	8259	697	5	5	4.22E-06
Exosome	biological process	biopolymer metabolism	GO:0043283	8259	1199	5	5	6.40E-05
FA	molecular function	cytoskeletal protein binding	GO:0008092	8974	239	38	13	7.35E-12
FA	molecular function	actin binding	GO:0003779	8974	164	38	11	4.26E-11
FA	molecular function	phorbol ester receptor activity	GO:0001565	8974	9	38	4	3.39E-08
FA	molecular function	protein kinase C activity	GO:0004697	8974	9	38	4	3.39E-08
FA	molecular function	protein kinase activity	GO:0004672	8974	451	38	12	1.84E-07
FA	molecular function	protein-tyrosine kinase activity	GO:0004713	8974	231	38	9	3.61E-07
FA	molecular function	protein binding	GO:0005515	8974	2073	38	23	8.00E-07
FA	molecular function	phosphotransferase activity. alcohol group as acceptor	GO:0016773	8974	528	38	12	1.01E-06
FA	molecular function	protein serine/threonine kinase activity	GO:0004674	8974	351	38	10	1.31E-06
FA	molecular function	kinase activity	GO:0016301	8974	627	38	12	6.10E-06
FA	molecular function	diacylglycerol binding	GO:0019992	8974	37	38	4	1.63E-05
FA	molecular function	transferase activity. transferring phosphorus-containing groups	GO:0016772	8974	714	38	12	2.30E-05
FA	cellular component	cytoskeleton	GO:0005856	7542	537	30	15	2.89E-10
FA	cellular component	actin cytoskeleton	GO:0015629	7542	157	30	8	1.17E-07
FA	cellular component	non-membrane-bound organelle	GO:0043228	7542	881	30	15	2.60E-07
FA	cellular component	intracellular non-membrane-bound organelle	GO:0043232	7542	881	30	15	2.60E-07
FA	cellular component	caveolar membrane	GO:0016599	7542	5	30	3	5.65E-07
FA	cellular component	caveola	GO:0005901	7542	6	30	3	1.13E-06
FA	biological process	cell communication	GO:0007154	8259	2280	34	26	4.16E-09

Protein complex	Ontology type	GO Term	GO ID	N	M	n	m	P-value
FA	biological process	phosphorus metabolism	GO:0006793	8259	622	34	15	5.81E-09
FA	biological process	phosphate metabolism	GO:0006796	8259	622	34	15	5.81E-09
FA	biological process	intracellular signaling cascade	GO:0007242	8259	797	34	16	2.03E-08
FA	biological process	protein amino acid phosphorylation	GO:0006468	8259	433	34	12	7.08E-08
FA	biological process	phosphorylation	GO:0016310	8259	501	34	12	3.50E-07
FA	biological process	signal transduction	GO:0007165	8259	1823	34	21	6.78E-07
LRS	molecular function	structural constituent of ribosome	GO:0003735	8974	138	18	18	7.38E-34
LRS	molecular function	structural molecule activity	GO:0005198	8974	463	18	18	4.88E-24
LRS	molecular function	RNA binding	GO:0003723	8974	412	18	11	3.99E-11
LRS	molecular function	rRNA binding	GO:0019843	8974	5	18	2	3.79E-05
LRS	cellular component	ribosome	GO:0005840	7542	118	18	18	8.22E-34
LRS	cellular component	ribonucleoprotein complex	GO:0030529	7542	247	18	18	1.02E-27
LRS	cellular component	large ribosomal subunit	GO:0015934	7542	24	18	11	7.03E-25
LRS	cellular component	cytosolic large ribosomal subunit (sensu Eukaryota)	GO:0005842	7542	15	18	10	8.02E-25
LRS	cellular component	cytosolic ribosome (sensu Eukaryota)	GO:0005830	7542	25	18	10	8.64E-22
LRS	cellular component	non-membrane-bound organelle	GO:0043228	7542	881	18	18	1.41E-17
LRS	cellular component	intracellular non-membrane-bound organelle	GO:0043232	7542	881	18	18	1.41E-17
LRS	cellular component	protein complex	GO:0043234	7542	1247	18	18	7.69E-15
LRS	cellular component	cytosol	GO:0005829	7542	213	18	10	9.40E-12
LRS	cellular component	cytoplasm	GO:0005737	7542	2255	18	18	3.48E-10
LRS	cellular component	organelle	GO:0043226	7542	4197	18	18	2.58E-05
LRS	cellular component	intracellular organelle	GO:0043229	7542	4197	18	18	2.58E-05
LRS	biological process	protein biosynthesis	GO:0006412	8259	397	18	18	1.29E-24
LRS	biological process	macromolecule biosynthesis	GO:0009059	8259	445	18	18	1.05E-23
LRS	biological process	cellular biosynthesis	GO:0044249	8259	697	18	18	3.85E-20
LRS	biological process	biosynthesis	GO:0009058	8259	728	18	18	8.51E-20
LRS	biological process	cellular protein metabolism	GO:0044267	8259	2195	18	18	4.16E-11
LRS	biological process	protein metabolism	GO:0019538	8259	2210	18	18	4.70E-11
LRS	biological process	cellular macromolecule metabolism	GO:0044260	8259	2356	18	18	1.49E-10
LRS	biological process	macromolecule metabolism	GO:0043170	8259	2471	18	18	3.53E-10
LRS	biological process	primary metabolism	GO:0044238	8259	4660	18	18	3.31E-05
LRS	biological process	cellular metabolism	GO:0044237	8259	4823	18	18	6.16E-05
Nucleosome	molecular function	DNA binding	GO:0003677	8974	1297	24	24	5.74E-21
Nucleosome	molecular function	nucleic acid binding	GO:0003676	8974	2085	24	24	5.53E-16
Nucleosome	cellular component	chromatin	GO:0000785	7542	100	24	24	0.00E+00
Nucleosome	cellular component	nucleosome	GO:0000786	7542	45	24	24	0.00E+00
Nucleosome	cellular component	chromosome	GO:0005694	7542	181	24	24	2.80E-40
Nucleosome	cellular component	non-membrane-bound organelle	GO:0043228	7542	881	24	24	3.15E-23
Nucleosome	cellular component	intracellular non-membrane-bound organelle	GO:0043232	7542	881	24	24	3.15E-23
Nucleosome	cellular component	protein complex	GO:0043234	7542	1247	24	24	1.45E-19
Nucleosome	cellular component	nucleus	GO:0005634	7542	2503	24	24	2.96E-12
Nucleosome	cellular component	membrane-bound organelle	GO:0043227	7542	3685	24	24	3.30E-08
Nucleosome	cellular component	intracellular membrane-bound organelle	GO:0043231	7542	3685	24	24	3.30E-08
Nucleosome	cellular component	organelle	GO:0043226	7542	4197	24	24	7.55E-07
Nucleosome	cellular component	intracellular organelle	GO:0043229	7542	4197	24	24	7.55E-07

Protein complex	Ontology type	GO Term	GO ID	N	M	n	m	P-value
Nucleosome	cellular component	intracellular	GO:0005622	7542	5078	24	24	7.40E-05
Nucleosome	biological process	chromatin assembly or disassembly	GO:0006333	8259	86	24	24	0.00E+00
Nucleosome	biological process	nucleosome assembly	GO:0006334	8259	54	24	24	0.00E+00
Nucleosome	biological process	establishment and/or maintenance of chromatin architecture	GO:0006325	8259	160	24	24	1.31E-42
Nucleosome	biological process	DNA packaging	GO:0006323	8259	166	24	24	3.41E-42
Nucleosome	biological process	protein complex assembly	GO:0006461	8259	188	24	24	8.36E-41
Nucleosome	biological process	chromosome organization and biogenesis (sensu Eukaryota)	GO:0007001	8259	196	24	24	2.43E-40
Nucleosome	biological process	chromosome organization and biogenesis	GO:0051276	8259	203	24	24	5.94E-40
Nucleosome	biological process	DNA metabolism	GO:0006259	8259	405	24	24	1.93E-32
Nucleosome	biological process	organelle organization and biogenesis	GO:0006996	8259	568	24	24	7.92E-29
Nucleosome	biological process	cell organization and biogenesis	GO:0016043	8259	697	24	24	1.18E-26
Nucleosome	biological process	biopolymer metabolism	GO:0043283	8259	1199	24	24	6.30E-21
Nucleosome	biological process	nucleobase. nucleoside. nucleotide and nucleic acid metabolism	GO:0006139	8259	2155	24	24	9.02E-15
Nucleosome	biological process	cellular protein metabolism	GO:0044267	8259	2195	24	24	1.41E-14
Nucleosome	biological process	protein metabolism	GO:0019538	8259	2210	24	24	1.66E-14
Nucleosome	biological process	cellular macromolecule metabolism	GO:0044260	8259	2356	24	24	7.75E-14
Nucleosome	biological process	macromolecule metabolism	GO:0043170	8259	2471	24	24	2.45E-13
Nucleosome	biological process	primary metabolism	GO:0044238	8259	4660	24	24	1.06E-06
Nucleosome	biological process	cellular metabolism	GO:0044237	8259	4823	24	24	2.42E-06
Nucleosome	biological process	metabolism	GO:0008152	8259	5119	24	24	1.01E-05
Proteasome	molecular function	threonine endopeptidase activity	GO:0004298	8974	18	19	13	5.95E-34
Proteasome	molecular function	endopeptidase activity	GO:0004175	8974	267	19	13	2.48E-16
Proteasome	molecular function	peptidase activity	GO:0008233	8974	389	19	13	3.36E-14
Proteasome	molecular function	hydrolase activity	GO:0016787	8974	1384	19	17	1.82E-12
Proteasome	molecular function	catalytic activity	GO:0003824	8974	3594	19	17	1.14E-05
Proteasome	cellular component	proteasome complex (sensu Eukaryota)	GO:0000502	7542	31	21	20	0.00E+00
Proteasome	cellular component	cytosol	GO:0005829	7542	213	21	21	1.09E-33
Proteasome	cellular component	proteasome core complex (sensu Eukaryota)	GO:0005839	7542	18	21	13	4.27E-32
Proteasome	cellular component	endoplasmic reticulum	GO:0005783	7542	363	21	20	5.38E-26
Proteasome	cellular component	protein complex	GO:0043234	7542	1247	21	21	3.35E-17
Proteasome	cellular component	cytoplasm	GO:0005737	7542	2255	21	21	9.13E-12
Proteasome	cellular component	nucleus	GO:0005634	7542	2503	21	21	8.24E-11
Proteasome	cellular component	membrane-bound organelle	GO:0043227	7542	3685	21	21	2.85E-07
Proteasome	cellular component	intracellular membrane-bound organelle	GO:0043231	7542	3685	21	21	2.85E-07
Proteasome	cellular component	organelle	GO:0043226	7542	4197	21	21	4.41E-06
Proteasome	cellular component	intracellular organelle	GO:0043229	7542	4197	21	21	4.41E-06
Proteasome	cellular component	proteasome regulatory particle (sensu Eukaryota)	GO:0005838	7542	3	21	2	2.21E-05
Proteasome	biological process	ubiquitin-dependent protein catabolism	GO:0006511	8259	99	17	13	1.07E-22
Proteasome	biological process	modification-dependent protein catabolism	GO:0019941	8259	99	17	13	1.07E-22

Protein complex	Ontology type	GO Term	GO ID	N	M	n	m	P-value
Proteasome	biological process	protein catabolism	GO:0030163	8259	438	17	17	1.54E-22
Proteasome	biological process	biopolymer catabolism	GO:0043285	8259	446	17	17	2.11E-22
Proteasome	biological process	macromolecule catabolism	GO:0009057	8259	533	17	17	4.59E-21
Proteasome	biological process	catabolism	GO:0009056	8259	680	17	17	3.05E-19
Proteasome	biological process	proteolysis and peptidolysis	GO:0006508	8259	427	17	15	4.92E-18
Proteasome	biological process	cellular protein catabolism	GO:0044257	8259	427	17	15	4.92E-18
Proteasome	biological process	cellular macromolecule catabolism	GO:0044265	8259	522	17	15	1.02E-16
Proteasome	biological process	cellular catabolism	GO:0044248	8259	632	17	15	1.82E-15
Proteasome	biological process	biopolymer metabolism	GO:0043283	8259	1199	17	17	5.13E-15
Proteasome	biological process	protein metabolism	GO:0019538	8259	2210	17	17	1.77E-10
Proteasome	biological process	macromolecule metabolism	GO:0043170	8259	2471	17	17	1.19E-09
Proteasome	biological process	cellular protein metabolism	GO:0044267	8259	2195	17	15	1.73E-07
Proteasome	biological process	cellular macromolecule metabolism	GO:0044260	8259	2356	17	15	4.78E-07
Proteasome	biological process	primary metabolism	GO:0044238	8259	4660	17	17	5.88E-05
RNA Pol II	molecular function	DNA-directed RNA polymerase activity	GO:0003899	8974	30	10	10	3.23E-26
RNA Pol II	molecular function	nucleotidyltransferase activity	GO:0016779	8974	80	10	10	1.77E-21
RNA Pol II	molecular function	transferase activity. transferring phosphorus-containing groups	GO:0016772	8974	714	10	10	9.59E-12
RNA Pol II	molecular function	transferase activity	GO:0016740	8974	1173	10	10	1.41E-09
RNA Pol II	molecular function	nucleic acid binding	GO:0003676	8974	2085	10	9	1.54E-05
RNA Pol II	cellular component	RNA polymerase complex	GO:0030880	7542	20	10	8	2.19E-20
RNA Pol II	cellular component	DNA-directed RNA polymerase II. core complex	GO:0005665	7542	15	10	7	2.80E-18
RNA Pol II	cellular component	DNA-directed RNA polymerase II. holoenzyme	GO:0016591	7542	52	10	7	5.75E-14
RNA Pol II	cellular component	nucleoplasm	GO:0005654	7542	190	10	7	6.50E-10
RNA Pol II	cellular component	nucleus	GO:0005634	7542	2503	10	10	1.60E-05
RNA Pol II	cellular component	protein complex	GO:0043234	7542	1247	10	8	1.80E-05
RNA Pol II	biological process	transcription	GO:0006350	8259	1410	10	10	2.05E-08
RNA Pol II	biological process	transcription from Pol II promoter	GO:0006366	8259	358	10	7	2.91E-08
RNA Pol II	biological process	nucleobase. nucleoside. nucleotide and nucleic acid metabolism	GO:0006139	8259	2155	10	10	1.44E-06
RNA Pol II	biological process	transcription. DNA-dependent	GO:0006351	8259	1315	10	8	1.35E-05
RNA Pol III	molecular function	DNA-directed RNA polymerase activity	GO:0003899	8974	30	6	6	8.20E-16
RNA Pol III	molecular function	nucleotidyltransferase activity	GO:0016779	8974	80	6	6	4.15E-13
RNA Pol III	molecular function	transferase activity. transferring phosphorus-containing groups	GO:0016772	8974	714	6	6	2.49E-07
RNA Pol III	molecular function	transferase activity	GO:0016740	8974	1173	6	6	4.93E-06
RNA Pol III	molecular function	DNA binding	GO:0003677	8974	1297	6	6	9.02E-06
RNA Pol III	cellular component	DNA-directed RNA polymerase III complex	GO:0005666	7542	5	6	4	5.566E-13
RNA Pol III	cellular component	RNA polymerase complex	GO:0030880	7542	20	6	4	5.377E-10
RNA Pol III	cellular component	nucleoplasm	GO:0005654	7542	190	6	4	5.629E-06
RNA Pol III	biological process	transcription from Pol III promoter	GO:0006383	8259	18	6	3	1.732E-07
RNA Pol III	biological process	regulation of transcription from Pol III promoter	GO:0006359	8259	6	6	2	6.589E-06

Protein complex	Ontology type	GO Term	GO ID	N	M	n	m	P-value
RNA Pol III	biological process	transcription	GO:0006350	8259	1410	6	6	2.454E-05
SRP	molecular function	7S RNA binding	GO:0008312	8974	2	5	2	2.484E-07
SRP	molecular function	RNA binding	GO:0003723	8974	412	5	4	2.111E-05
SRP	cellular component	signal recognition particle (sensu Eukaryota)	GO:0005786	7542	9	5	5	6.204E-16
SRP	cellular component	signal recognition particle	GO:0048500	7542	9	5	5	6.204E-16
SRP	biological process	cotranslational protein-membrane targeting	GO:0006613	8259	11	4	3	7.027E-09
SRP	biological process	protein-ER targeting	GO:0045047	8259	11	4	3	7.027E-09
SRP	biological process	protein-membrane targeting	GO:0006612	8259	19	4	3	4.124E-08
SRP	biological process	secretory pathway	GO:0045045	8259	118	4	3	1.126E-05
SRP	biological process	protein targeting	GO:0006605	8259	122	4	3	1.245E-05
SRP	biological process	secretion	GO:0046903	8259	152	4	3	2.412E-05
SRS	molecular function	structural constituent of ribosome	GO:0003735	8974	138	20	20	1.315E-37
SRS	molecular function	structural molecule activity	GO:0005198	8974	463	20	20	1.203E-26
SRS	cellular component	ribosome	GO:0005840	7542	118	20	18	1.522E-31
SRS	cellular component	ribonucleoprotein complex	GO:0030529	7542	247	20	20	9.374E-31
SRS	cellular component	protein complex	GO:0043234	7542	1247	20	20	2.051E-16
SRS	cellular component	non-membrane-bound organelle	GO:0043228	7542	881	20	18	2.121E-15
SRS	cellular component	intracellular non-membrane-bound organelle	GO:0043232	7542	881	20	18	2.121E-15
SRS	cellular component	small ribosomal subunit	GO:0015935	7542	30	20	8	2.763E-15
SRS	cellular component	cytosolic small ribosomal subunit (sensu Eukaryota)	GO:0005843	7542	10	20	6	3.17E-14
SRS	cellular component	eukaryotic 48S initiation complex	GO:0016283	7542	10	20	6	3.17E-14
SRS	cellular component	eukaryotic 43S preinitiation complex	GO:0016282	7542	23	20	6	1.493E-11
SRS	cellular component	cytosolic ribosome (sensu Eukaryota)	GO:0005830	7542	25	20	6	2.61E-11
SRS	cellular component	cytoplasm	GO:0005737	7542	2255	20	18	3.41E-08
SRS	cellular component	cytosol	GO:0005829	7542	213	20	6	1.316E-05
SRS	biological process	protein biosynthesis	GO:0006412	8259	397	18	18	1.293E-24
SRS	biological process	macromolecule biosynthesis	GO:0009059	8259	445	18	18	1.053E-23
SRS	biological process	cellular biosynthesis	GO:0044249	8259	697	18	18	3.85E-20
SRS	biological process	biosynthesis	GO:0009058	8259	728	18	18	8.506E-20
SRS	biological process	cellular protein metabolism	GO:0044267	8259	2195	18	18	4.157E-11
SRS	biological process	protein metabolism	GO:0019538	8259	2210	18	18	4.702E-11
SRS	biological process	cellular macromolecule metabolism	GO:0044260	8259	2356	18	18	1.494E-10
SRS	biological process	macromolecule metabolism	GO:0043170	8259	2471	18	18	3.533E-10
SRS	biological process	primary metabolism	GO:0044238	8259	4660	18	18	3.312E-05
SRS	biological process	cellular metabolism	GO:0044237	8259	4823	18	18	6.156E-05
TAFIID	molecular function	general RNA polymerase II transcription factor activity	GO:0016251	8974	28	13	6	8.796E-13
TAFIID	molecular function	transcription regulator activity	GO:0030528	8974	865	13	12	7.117E-12
TAFIID	molecular function	RNA polymerase II transcription factor activity	GO:0003702	8974	164	13	8	1.249E-11
TAFIID	molecular function	DNA binding	GO:0003677	8974	1297	13	12	8.969E-10
TAFIID	molecular function	nucleic acid binding	GO:0003676	8974	2085	13	12	2.469E-07
TAFIID	molecular function	transcription factor activity	GO:0003700	8974	591	13	8	3.236E-07
TAFIID	molecular function	transcription initiation factor activity	GO:0016986	8974	5	13	2	1.933E-05
TAFIID	cellular component	transcription factor TFIID complex	GO:0005669	7542	14	13	10	1.753E-27

Protein complex	Ontology type	GO Term	GO ID	N	M	n	m	P-value
TAFIID	cellular component	transcription factor complex	GO:0005667	7542	77	13	11	4.591E-21
TAFIID	cellular component	DNA-directed RNA polymerase II. holoenzyme	GO:0016591	7542	52	13	10	2.732E-20
TAFIID	cellular component	nucleoplasm	GO:0005654	7542	190	13	11	1.452E-16
TAFIID	cellular component	protein complex	GO:0043234	7542	1247	13	11	1.37E-07
TAFIID	cellular component	nucleus	GO:0005634	7542	2503	13	13	5.802E-07
TAFIID	cellular component	membrane-bound organelle	GO:0043227	7542	3685	13	13	8.946E-05
TAFIID	cellular component	intracellular membrane-bound organelle	GO:0043231	7542	3685	13	13	8.946E-05
TAFIID	biological process	transcription initiation	GO:0006352	8259	41	13	8	2.258E-16
TAFIID	biological process	transcription	GO:0006350	8259	1410	13	13	9.995E-11
TAFIID	biological process	transcription. DNA-dependent	GO:0006351	8259	1315	13	12	2.825E-09
TAFIID	biological process	regulation of transcription	GO:0045449	8259	1346	13	12	3.726E-09
TAFIID	biological process	regulation of nucleobase. nucleoside. nucleotide and nucleic acid metabolism	GO:0019219	8259	1372	13	12	4.676E-09
TAFIID	biological process	regulation of metabolism	GO:0019222	8259	1494	13	12	1.284E-08
TAFIID	biological process	nucleobase. nucleoside. nucleotide and nucleic acid metabolism	GO:0006139	8259	2155	13	13	2.53E-08
TAFIID	biological process	regulation of transcription. DNA-dependent	GO:0006355	8259	1268	13	11	6.217E-08
TAFIID	biological process	regulation of physiological process	GO:0050791	8259	1892	13	12	2.087E-07
TAFIID	biological process	regulation of biological process	GO:0050789	8259	2157	13	12	9.726E-07
TAFIID	biological process	regulation of transcription factor activity	GO:0051090	8259	6	13	2	3.419E-05
VHL	biological process	ubiquitin cycle	GO:0006512	8259	435	4	4	7.596E-06

C

Protein complex	Ontology type	GO Term	GO ID	N	M	n	m	P-value
Arp2-3	molecular function	structural molecule activity	GO:0005198	9140	473	5	5	3.64E-07
Arp2-3	molecular function	structural constituent of cytoskeleton	GO:0005200	9140	67	5	3	3.73E-06
Arp2-3	cellular component	Arp2/3 protein complex	GO:0005885	7684	4	5	4	3.44E-14
Arp2-3	cellular component	actin cytoskeleton	GO:0015629	7684	159	5	4	8.69E-07
Arp2-3	cellular component	cytoskeleton	GO:0005856	7684	545	5	5	1.76E-06
Arp2-3	cellular component	non-membrane-bound organelle	GO:0043228	7684	893	5	5	2.10E-05
Arp2-3	cellular component	intracellular non-membrane-bound organelle	GO:0043232	7684	893	5	5	2.10E-05
Arp2-3	biological process	regulation of actin filament polymerization	GO:0030833	8419	7	4	3	1.41E-09
Arp2-3	biological process	actin filament polymerization	GO:0030041	8419	12	4	3	8.84E-09
Arp2-3	biological process	protein polymerization	GO:0051258	8419	24	4	3	8.13E-08
Arp2-3	biological process	cell motility	GO:0006928	8419	153	4	4	1.05E-07
Arp2-3	biological process	regulation of actin polymerization and/or depolymerization	GO:0008064	8419	27	4	3	1.17E-07
Arp2-3	biological process	regulation of actin filament length	GO:0030832	8419	27	4	3	1.17E-07

Protein complex	Ontology type	GO Term	GO ID	N	M	n	m	P-value
Arp2-3	biological process	actin polymerization and/or depolymerization	GO:0008154	8419	35	4	3	2.63E-07
Arp2-3	biological process	regulation of cell organization and biogenesis	GO:0051128	8419	39	4	3	3.67E-07
Arp2-3	biological process	regulation of protein metabolism	GO:0051246	8419	71	4	3	2.29E-06
Arp2-3	biological process	actin cytoskeleton organization and biogenesis	GO:0030036	8419	92	4	3	5.01E-06
Arp2-3	biological process	actin filament-based process	GO:0030029	8419	99	4	3	6.26E-06
APC	molecular function	ubiquitin-protein ligase activity	GO:0004842	9140	296	7	4	3.49E-05
APC	molecular function	acid-amino acid ligase activity	GO:0016881	9140	312	7	4	4.30E-05
APC	molecular function	ligase activity. forming carbon-nitrogen bonds	GO:0016879	9140	337	7	4	5.82E-05
APC	cellular component	nuclear ubiquitin ligase complex	GO:0000152	7684	9	7	6	2.06E-18
APC	cellular component	anaphase-promoting complex	GO:0005680	7684	9	7	6	2.06E-18
APC	cellular component	ubiquitin ligase complex	GO:0000151	7684	255	7	6	8.58E-09
APC	biological process	mitosis	GO:0007067	8419	115	8	8	9.48E-16
APC	biological process	M phase of mitotic cell cycle	GO:0000087	8419	117	8	8	1.09E-15
APC	biological process	M phase	GO:0000279	8419	142	8	8	5.38E-15
APC	biological process	mitotic cell cycle	GO:0000278	8419	173	8	8	2.71E-14
APC	biological process	cell division	GO:0051301	8419	122	8	7	8.92E-13
APC	biological process	cell cycle	GO:0007049	8419	514	8	8	1.83E-10
APC	biological process	mitotic metaphase/anaphase transition	GO:0007091	8419	3	8	3	5.63E-10
APC	biological process	regulation of cell cycle	GO:0000074	8419	338	8	7	1.22E-09
APC	biological process	mitotic anaphase	GO:0000090	8419	5	8	3	5.63E-09
APC	biological process	anaphase	GO:0051322	8419	5	8	3	5.63E-09
APC	biological process	ubiquitin cycle	GO:0006512	8419	438	8	7	7.53E-09
APC	biological process	interphase	GO:0051325	8419	69	8	4	2.82E-07
APC	biological process	interphase of mitotic cell cycle	GO:0051329	8419	69	8	4	2.82E-07
APC	biological process	regulation of mitotic metaphase/anaphase transition	GO:0030071	8419	2	8	2	7.90E-07
APC	biological process	ubiquitin-dependent protein catabolism	GO:0006511	8419	105	8	4	1.54E-06
APC	biological process	modification-dependent protein catabolism	GO:0019941	8419	105	8	4	1.54E-06
APC	biological process	regulation of mitosis	GO:0007088	8419	30	8	3	2.26E-06
APC	biological process	G2/M transition of mitotic cell cycle	GO:0000086	8419	40	8	3	5.47E-06
APC	biological process	protein modification	GO:0006464	8419	1253	8	7	1.11E-05
Centrosome	molecular function	tubulin binding	GO:0015631	9140	29	32	5	4.24E-08
Centrosome	molecular function	motor activity	GO:0003774	9140	108	32	6	1.67E-06
Centrosome	molecular function	microtubule binding	GO:0008017	9140	21	32	3	4.97E-05
Centrosome	cellular component	microtubule cytoskeleton	GO:0015630	7684	179	32	23	1.55E-31
Centrosome	cellular component	microtubule organizing center	GO:0005815	7684	39	32	14	2.37E-25
Centrosome	cellular component	spindle	GO:0005819	7684	50	32	14	1.44E-23
Centrosome	cellular component	cytoskeleton	GO:0005856	7684	545	32	25	2.32E-23
Centrosome	cellular component	spindle pole	GO:0000922	7684	40	32	13	7.59E-23
Centrosome	cellular component	centrosome	GO:0005813	7684	35	32	12	2.03E-21
Centrosome	cellular component	microtubule	GO:0005874	7684	127	32	15	3.60E-19
Centrosome	cellular component	non-membrane-bound organelle	GO:0043228	7684	893	32	25	4.76E-18
Centrosome	cellular component	intracellular non-membrane-bound organelle	GO:0043232	7684	893	32	25	4.76E-18

Protein complex	Ontology type	GO Term	GO ID	N	M	n	m	P-value
Centrosome	cellular component	microtubule associated complex	GO:0005875	7684	76	32	7	1.93E-08
Centrosome	cellular component	dynein complex	GO:0030286	7684	22	32	5	2.26E-08
Centrosome	cellular component	gamma-tubulin complex	GO:0000930	7684	5	32	3	6.52E-07
Centrosome	cellular component	spindle microtubule	GO:0005876	7684	7	32	3	2.27E-06
Centrosome	cellular component	cAMP-dependent protein kinase complex	GO:0005952	7684	8	32	3	3.62E-06
Centrosome	cellular component	organelle	GO:0043226	7684	4267	32	29	1.87E-05
Centrosome	cellular component	intracellular organelle	GO:0043229	7684	4267	32	29	1.87E-05
Centrosome	cellular component	intracellular	GO:0005622	7684	5168	32	31	4.96E-05
Centrosome	cellular component	centriole	GO:0005814	7684	3	32	2	5.03E-05
Centrosome	cellular component	polar microtubule	GO:0005827	7684	3	32	2	5.03E-05
Centrosome	biological process	microtubule-based process	GO:0007017	8419	109	27	12	1.77E-16
Centrosome	biological process	cytoskeleton organization and biogenesis	GO:0007010	8419	258	27	13	2.13E-13
Centrosome	biological process	mitosis	GO:0007067	8419	115	27	10	1.06E-12
Centrosome	biological process	microtubule cytoskeleton organization and biogenesis	GO:0000226	8419	48	27	8	1.24E-12
Centrosome	biological process	M phase of mitotic cell cycle	GO:0000087	8419	117	27	10	1.26E-12
Centrosome	biological process	mitotic cell cycle	GO:0000278	8419	173	27	11	1.97E-12
Centrosome	biological process	M phase	GO:0000279	8419	142	27	10	8.95E-12
Centrosome	biological process	organelle organization and biogenesis	GO:0006996	8419	573	27	15	2.11E-11
Centrosome	biological process	microtubule nucleation	GO:0007020	8419	12	27	5	1.79E-10
Centrosome	biological process	cell organization and biogenesis	GO:0016043	8419	708	27	15	4.30E-10
Centrosome	biological process	cell cycle	GO:0007049	8419	514	27	12	1.77E-08
Centrosome	biological process	microtubule-based movement	GO:0007018	8419	57	27	4	2.95E-05
Centrosome	biological process	cytoskeleton-dependent intracellular transport	GO:0030705	8419	57	27	4	2.95E-05
Centrosome	biological process	cell division	GO:0051301	8419	122	27	5	3.68E-05
Centrosome	biological process	protein polymerization	GO:0051258	8419	24	27	3	5.69E-05
Dynactin	molecular function	motor activity	GO:0003774	9140	108	7	3	5.43E-05
Dynactin	cellular component	cytoskeleton	GO:0005856	7684	545	7	7	8.71E-09
Dynactin	cellular component	F-actin capping protein complex	GO:0008290	7684	7	7	3	1.62E-08
Dynactin	cellular component	actin cytoskeleton	GO:0015629	7684	159	7	5	7.24E-08
Dynactin	cellular component	non-membrane-bound organelle	GO:0043228	7684	893	7	7	2.80E-07
Dynactin	cellular component	intracellular non-membrane-bound organelle	GO:0043232	7684	893	7	7	2.80E-07
Dynactin	biological process	actin filament depolymerization	GO:0030042	8419	18	6	3	1.63E-07
Dynactin	biological process	regulation of actin filament depolymerization	GO:0030834	8419	18	6	3	1.63E-07
Dynactin	biological process	negative regulation of actin filament depolymerization	GO:0030835	8419	18	6	3	1.63E-07
Dynactin	biological process	barbed-end actin filament capping	GO:0051016	8419	18	6	3	1.63E-07
Dynactin	biological process	negative regulation of cell organization and biogenesis	GO:0051129	8419	26	6	3	5.20E-07
Dynactin	biological process	protein depolymerization	GO:0051261	8419	26	6	3	5.20E-07
Dynactin	biological process	regulation of actin polymerization and/or depolymerization	GO:0008064	8419	27	6	3	5.85E-07
Dynactin	biological process	regulation of actin filament length	GO:0030832	8419	27	6	3	5.85E-07

Protein complex	Ontology type	GO Term	GO ID	N	M	n	m	P-value
Dynactin	biological process	actin polymerization and/or depolymerization	GO:0008154	8419	35	6	3	1.31E-06
Dynactin	biological process	negative regulation of protein metabolism	GO:0051248	8419	38	6	3	1.68E-06
Dynactin	biological process	regulation of cell organization and biogenesis	GO:0051128	8419	39	6	3	1.82E-06
Dynactin	biological process	organelle organization and biogenesis	GO:0006996	8419	573	6	5	8.14E-06
Dynactin	biological process	regulation of protein metabolism	GO:0051246	8419	71	6	3	1.13E-05
Dynactin	biological process	cytoskeleton organization and biogenesis	GO:0007010	8419	258	6	4	1.23E-05
Dynactin	biological process	cell organization and biogenesis	GO:0016043	8419	708	6	5	2.32E-05
Dynactin	biological process	actin cytoskeleton organization and biogenesis	GO:0030036	8419	92	6	3	2.47E-05
Dynactin	biological process	actin filament-based process	GO:0030029	8419	99	6	3	3.08E-05
Exocyst	cellular component	exocyst	GO:0000145	7684	5	6	5	2.69E-17
Exocyst	cellular component	cell cortex	GO:0005938	7684	20	6	5	4.17E-13
Exocyst	biological process	exocytosis	GO:0006887	8419	50	7	7	1.68E-16
Exocyst	biological process	secretory pathway	GO:0045045	8419	118	7	7	8.89E-14
Exocyst	biological process	secretion	GO:0046903	8419	151	7	7	5.20E-13
Exocyst	biological process	vesicle-mediated transport	GO:0016192	8419	254	7	7	2.10E-11
Exocyst	biological process	vesicle docking	GO:0048278	8419	19	7	4	6.46E-10
Exocyst	biological process	protein transport	GO:0015031	8419	441	7	7	1.03E-09
Exocyst	biological process	establishment of protein localization	GO:0045184	8419	443	7	7	1.07E-09
Exocyst	biological process	protein localization	GO:0008104	8419	457	7	7	1.33E-09
Exocyst	biological process	vesicle docking during exocytosis	GO:0006904	8419	18	7	3	2.86E-07
Exocyst	biological process	transport	GO:0006810	8419	1591	7	7	8.52E-06
Exocyst	biological process	establishment of localization	GO:0051234	8419	1593	7	7	8.59E-06
Exocyst	biological process	localization	GO:0051179	8419	1606	7	7	9.09E-06
Exosome	molecular function	exonuclease activity	GO:0004527	9140	41	5	5	1.41E-12
Exosome	molecular function	nuclease activity	GO:0004518	9140	95	5	5	1.09E-10
Exosome	molecular function	3'-5' exonuclease activity	GO:0008408	9140	22	5	4	1.26E-10
Exosome	molecular function	3'-5'-exoribonuclease activity	GO:0000175	9140	8	5	3	4.40E-09
Exosome	molecular function	exoribonuclease activity	GO:0004532	9140	10	5	3	9.42E-09
Exosome	molecular function	exoribonuclease activity. producing 5'-phosphomonoesters	GO:0016896	9140	10	5	3	9.42E-09
Exosome	molecular function	exonuclease activity. active with either ribo- or deoxyribonucleic acids and producing 5'-phosphomonoesters	GO:0016796	9140	12	5	3	1.73E-08
Exosome	molecular function	RNA binding	GO:0003723	9140	415	5	5	1.89E-07
Exosome	molecular function	hydrolase activity. acting on ester bonds	GO:0016788	9140	451	5	5	2.86E-07
Exosome	molecular function	ribonuclease activity	GO:0004540	9140	39	5	3	7.14E-07
Exosome	molecular function	hydrolase activity	GO:0016787	9140	1414	5	5	8.81E-05
Exosome	cellular component	exosome (RNase complex)	GO:0000178	7684	6	5	4	5.17E-13
Exosome	cellular component	nucleolus	GO:0005730	7684	71	5	4	3.32E-08
Exosome	biological process	rRNA processing	GO:0006364	8419	29	5	5	3.37E-13
Exosome	biological process	rRNA metabolism	GO:0016072	8419	33	5	5	6.74E-13
Exosome	biological process	ribosome biogenesis	GO:0007046	8419	45	5	5	3.47E-12

Protein complex	Ontology type	GO Term	GO ID	N	M	n	m	P-value
Exosome	biological process	ribosome biogenesis and assembly	GO:0042254	8419	50	5	5	6.02E-12
Exosome	biological process	cytoplasm organization and biogenesis	GO:0007028	8419	62	5	5	1.84E-11
Exosome	biological process	RNA processing	GO:0006396	8419	263	5	5	2.87E-08
Exosome	biological process	RNA metabolism	GO:0016070	8419	334	5	5	9.55E-08
Exosome	biological process	organelle organization and biogenesis	GO:0006996	8419	573	5	5	1.44E-06
Exosome	biological process	cell organization and biogenesis	GO:0016043	8419	708	5	5	4.15E-06
Exosome	biological process	biopolymer metabolism	GO:0043283	8419	1223	5	5	6.42E-05
FA	molecular function	cytoskeletal protein binding	GO:0008092	9140	239	40	14	6.07E-13
FA	molecular function	actin binding	GO:0003779	9140	163	40	11	6.12E-11
FA	molecular function	phorbol ester receptor activity	GO:0001565	9140	10	40	5	3.07E-10
FA	molecular function	protein kinase C activity	GO:0004697	9140	10	40	5	3.07E-10
FA	molecular function	protein kinase activity	GO:0004672	9140	463	40	13	4.19E-08
FA	molecular function	protein serine/threonine kinase activity	GO:0004674	9140	357	40	11	2.31E-07
FA	molecular function	phosphotransferase activity. alcohol group as acceptor	GO:0016773	9140	540	40	13	2.54E-07
FA	molecular function	protein binding	GO:0005515	9140	2108	40	24	5.61E-07
FA	molecular function	protein-tyrosine kinase activity	GO:0004713	9140	235	40	9	5.75E-07
FA	molecular function	diacylglycerol binding	GO:0019992	9140	39	40	5	6.40E-07
FA	molecular function	kinase activity	GO:0016301	9140	642	40	13	1.84E-06
FA	molecular function	transferase activity. transferring phosphorus-containing groups	GO:0016772	9140	728	40	13	7.43E-06
FA	cellular component	cytoskeleton	GO:0005856	7684	545	30	15	2.74E-10
FA	cellular component	actin cytoskeleton	GO:0015629	7684	159	30	8	1.12E-07
FA	cellular component	non-membrane-bound organelle	GO:0043228	7684	893	30	15	2.44E-07
FA	cellular component	intracellular non-membrane-bound organelle	GO:0043232	7684	893	30	15	2.44E-07
FA	cellular component	caveolar membrane	GO:0016599	7684	4	30	2	8.80E-05
FA	biological process	cell communication	GO:0007154	8419	2315	34	27	4.14E-10
FA	biological process	phosphorus metabolism	GO:0006793	8419	634	34	16	5.44E-10
FA	biological process	phosphate metabolism	GO:0006796	8419	634	34	16	5.44E-10
FA	biological process	intracellular signaling cascade	GO:0007242	8419	809	34	17	2.09E-09
FA	biological process	protein amino acid phosphorylation	GO:0006468	8419	443	34	13	6.70E-09
FA	biological process	phosphorylation	GO:0016310	8419	513	34	13	3.90E-08
FA	biological process	signal transduction	GO:0007165	8419	1850	34	22	1.01E-07
FA	biological process	protein modification	GO:0006464	8419	1253	34	15	4.06E-05
LRS	molecular function	structural constituent of ribosome	GO:0003735	9140	140	18	18	7.00E-34
LRS	molecular function	structural molecule activity	GO:0005198	9140	473	18	18	5.19E-24
LRS	molecular function	RNA binding	GO:0003723	9140	415	18	11	3.55E-11
LRS	molecular function	rRNA binding	GO:0019843	9140	5	18	2	3.65E-05
LRS	cellular component	ribosome	GO:0005840	7684	119	18	18	6.92E-34
LRS	cellular component	ribonucleoprotein complex	GO:0030529	7684	251	18	18	9.80E-28
LRS	cellular component	large ribosomal subunit	GO:0015934	7684	24	18	11	5.73E-25
LRS	cellular component	cytosolic large ribosomal subunit (sensu Eukaryota)	GO:0005842	7684	15	18	10	6.65E-25

Protein complex	Ontology type	GO Term	GO ID	N	M	n	m	P-value
LRS	cellular component	cytosolic ribosome (sensu Eukaryota)	GO:0005830	7684	26	18	10	1.16E-21
LRS	cellular component	non-membrane-bound organelle	GO:0043228	7684	893	18	18	1.28E-17
LRS	cellular component	intracellular non-membrane-bound organelle	GO:0043232	7684	893	18	18	1.28E-17
LRS	cellular component	protein complex	GO:0043234	7684	1274	18	18	8.10E-15
LRS	cellular component	cytosol	GO:0005829	7684	226	18	10	1.42E-11
LRS	cellular component	cytoplasm	GO:0005737	7684	2299	18	18	3.52E-10
LRS	cellular component	organelle	GO:0043226	7684	4267	18	18	2.48E-05
LRS	cellular component	intracellular organelle	GO:0043229	7684	4267	18	18	2.48E-05
LRS	biological process	protein biosynthesis	GO:0006412	8419	400	18	18	1.05E-24
LRS	biological process	macromolecule biosynthesis	GO:0009059	8419	447	18	18	8.09E-24
LRS	biological process	cellular biosynthesis	GO:0044249	8419	706	18	18	3.44E-20
LRS	biological process	biosynthesis	GO:0009058	8419	737	18	18	7.53E-20
LRS	biological process	cellular protein metabolism	GO:0044267	8419	2230	18	18	3.92E-11
LRS	biological process	protein metabolism	GO:0019538	8419	2246	18	18	4.46E-11
LRS	biological process	cellular macromolecule metabolism	GO:0044260	8419	2394	18	18	1.41E-10
LRS	biological process	macromolecule metabolism	GO:0043170	8419	2512	18	18	3.37E-10
LRS	biological process	primary metabolism	GO:0044238	8419	4747	18	18	3.27E-05
LRS	biological process	cellular metabolism	GO:0044237	8419	4914	18	18	6.10E-05
Nucleosome	molecular function	DNA binding	GO:0003677	9140	1324	24	24	6.09E-21
Nucleosome	molecular function	nucleic acid binding	GO:0003676	9140	2115	24	24	5.02E-16
Nucleosome	cellular component	chromatin	GO:0000785	7684	100	24	24	0.00E+00
Nucleosome	cellular component	nucleosome	GO:0000786	7684	45	24	24	0.00E+00
Nucleosome	cellular component	chromosome	GO:0005694	7684	182	24	24	2.06E-40
Nucleosome	cellular component	non-membrane-bound organelle	GO:0043228	7684	893	24	24	2.80E-23
Nucleosome	cellular component	intracellular non-membrane-bound organelle	GO:0043232	7684	893	24	24	2.80E-23
Nucleosome	cellular component	protein complex	GO:0043234	7684	1274	24	24	1.55E-19
Nucleosome	cellular component	nucleus	GO:0005634	7684	2551	24	24	2.99E-12
Nucleosome	cellular component	membrane-bound organelle	GO:0043227	7684	3750	24	24	3.21E-08
Nucleosome	cellular component	intracellular membrane-bound organelle	GO:0043231	7684	3750	24	24	3.21E-08
Nucleosome	cellular component	organelle	GO:0043226	7684	4267	24	24	7.18E-07
Nucleosome	cellular component	intracellular organelle	GO:0043229	7684	4267	24	24	7.18E-07
Nucleosome	cellular component	intracellular	GO:0005622	7684	5168	24	24	7.21E-05
Nucleosome	biological process	chromatin assembly or disassembly	GO:0006333	8419	85	24	24	0.00E+00
Nucleosome	biological process	nucleosome assembly	GO:0006334	8419	54	24	24	0.00E+00
Nucleosome	biological process	establishment and/or maintenance of chromatin architecture	GO:0006325	8419	161	24	24	9.73E-43
Nucleosome	biological process	DNA packaging	GO:0006323	8419	167	24	24	2.51E-42
Nucleosome	biological process	protein complex assembly	GO:0006461	8419	190	24	24	6.91E-41
Nucleosome	biological process	chromosome organization and biogenesis (sensu Eukaryota)	GO:0007001	8419	197	24	24	1.74E-40
Nucleosome	biological process	chromosome organization and biogenesis	GO:0051276	8419	204	24	24	4.24E-40
Nucleosome	biological process	DNA metabolism	GO:0006259	8419	411	24	24	1.75E-32
Nucleosome	biological process	organelle organization and biogenesis	GO:0006996	8419	573	24	24	6.19E-29
Nucleosome	biological process	cell organization and biogenesis	GO:0016043	8419	708	24	24	1.09E-26

Protein complex	Ontology type	GO Term	GO ID	N	M	n	m	P-value
Nucleosome	biological process	biopolymer metabolism	GO:0043283	8419	1223	24	24	6.42E-21
Nucleosome	biological process	nucleobase. nucleoside. nucleotide and nucleic acid metabolism	GO:0006139	8419	2192	24	24	8.58E-15
Nucleosome	biological process	cellular protein metabolism	GO:0044267	8419	2230	24	24	1.30E-14
Nucleosome	biological process	protein metabolism	GO:0019538	8419	2246	24	24	1.54E-14
Nucleosome	biological process	cellular macromolecule metabolism	GO:0044260	8419	2394	24	24	7.19E-14
Nucleosome	biological process	macromolecule metabolism	GO:0043170	8419	2512	24	24	2.29E-13
Nucleosome	biological process	primary metabolism	GO:0044238	8419	4747	24	24	1.04E-06
Nucleosome	biological process	cellular metabolism	GO:0044237	8419	4914	24	24	2.39E-06
Nucleosome	biological process	metabolism	GO:0008152	8419	5217	24	24	1.01E-05
Proteasome	molecular function	threonine endopeptidase activity	GO:0004298	9140	19	20	13	4.23E-33
Proteasome	molecular function	endopeptidase activity	GO:0004175	9140	274	20	13	7.66E-16
Proteasome	molecular function	peptidase activity	GO:0008233	9140	398	20	13	9.81E-14
Proteasome	molecular function	hydrolase activity	GO:0016787	9140	1414	20	18	3.26E-13
Proteasome	molecular function	catalytic activity	GO:0003824	9140	3662	20	18	5.07E-06
Proteasome	cellular component	proteasome complex (sensu Eukaryota)	GO:0000502	7684	35	23	21	0.00E+00
Proteasome	cellular component	cytosol	GO:0005829	7684	226	23	23	1.94E-36
Proteasome	cellular component	proteasome core complex (sensu Eukaryota)	GO:0005839	7684	19	23	13	5.95E-31
Proteasome	cellular component	endoplasmic reticulum	GO:0005783	7684	377	23	21	4.32E-26
Proteasome	cellular component	protein complex	GO:0043234	7684	1274	23	23	9.50E-19
Proteasome	cellular component	cytoplasm	GO:0005737	7684	2299	23	23	8.19E-13
Proteasome	cellular component	nucleus	GO:0005634	7684	2551	23	23	9.06E-12
Proteasome	cellular component	membrane-bound organelle	GO:0043227	7684	3750	23	23	6.59E-08
Proteasome	cellular component	intracellular membrane-bound organelle	GO:0043231	7684	3750	23	23	6.59E-08
Proteasome	cellular component	proteasome regulatory particle (sensu Eukaryota)	GO:0005838	7684	5	23	3	2.33E-07
Proteasome	cellular component	organelle	GO:0043226	7684	4267	23	23	1.30E-06
Proteasome	cellular component	intracellular organelle	GO:0043229	7684	4267	23	23	1.30E-06
Proteasome	biological process	protein catabolism	GO:0030163	8419	452	19	19	5.13E-25
Proteasome	biological process	biopolymer catabolism	GO:0043285	8419	461	19	19	7.51E-25
Proteasome	biological process	macromolecule catabolism	GO:0009057	8419	549	19	19	2.21E-23
Proteasome	biological process	ubiquitin-dependent protein catabolism	GO:0006511	8419	105	19	13	2.10E-21
Proteasome	biological process	modification-dependent protein catabolism	GO:0019941	8419	105	19	13	2.10E-21
Proteasome	biological process	catabolism	GO:0009056	8419	699	19	19	2.33E-21
Proteasome	biological process	proteolysis and peptidolysis	GO:0006508	8419	440	19	16	2.00E-18
Proteasome	biological process	cellular protein catabolism	GO:0044257	8419	440	19	16	2.00E-18
Proteasome	biological process	cellular macromolecule catabolism	GO:0044265	8419	537	19	16	4.92E-17
Proteasome	biological process	biopolymer metabolism	GO:0043283	8419	1223	19	19	1.07E-16
Proteasome	biological process	cellular catabolism	GO:0044248	8419	650	19	16	1.04E-15
Proteasome	biological process	protein metabolism	GO:0019538	8419	2246	19	19	1.18E-11
Proteasome	biological process	macromolecule metabolism	GO:0043170	8419	2512	19	19	9.99E-11
Proteasome	biological process	cellular protein metabolism	GO:0044267	8419	2230	19	16	2.33E-07
Proteasome	biological process	cellular macromolecule metabolism	GO:0044260	8419	2394	19	16	6.75E-07
Proteasome	biological process	primary metabolism	GO:0044238	8419	4747	19	19	1.84E-05
RNA Pol II	molecular function	DNA-directed RNA polymerase activity	GO:0003899	9140	29	10	10	1.80E-26

Protein complex	Ontology type	GO Term	GO ID	N	M	n	m	P-value
RNA Pol II	molecular function	nucleotidyltransferase activity	GO:0016779	9140	79	10	10	1.29E-21
RNA Pol II	molecular function	transferase activity. transferring phosphorus-containing groups	GO:0016772	9140	728	10	10	9.71E-12
RNA Pol II	molecular function	transferase activity	GO:0016740	9140	1197	10	10	1.44E-09
RNA Pol II	molecular function	nucleic acid binding	GO:0003676	9140	2115	10	9	1.49E-05
RNA Pol II	cellular component	RNA polymerase complex	GO:0030880	7684	19	10	8	1.13E-20
RNA Pol II	cellular component	DNA-directed RNA polymerase II. core complex	GO:0005665	7684	15	10	7	2.46E-18
RNA Pol II	cellular component	DNA-directed RNA polymerase II. holoenzyme	GO:0016591	7684	54	10	7	6.68E-14
RNA Pol II	cellular component	nucleoplasm	GO:0005654	7684	193	10	7	6.37E-10
RNA Pol II	cellular component	nucleus	GO:0005634	7684	2551	10	10	1.61E-05
RNA Pol II	cellular component	protein complex	GO:0043234	7684	1274	10	8	1.84E-05
RNA Pol II	biological process	transcription	GO:0006350	8419	1436	10	10	2.03E-08
RNA Pol II	biological process	transcription from Pol II promoter	GO:0006366	8419	367	10	7	3.03E-08
RNA Pol II	biological process	nucleobase. nucleoside. nucleotide and nucleic acid metabolism	GO:0006139	8419	2192	10	10	1.41E-06
RNA Pol II	biological process	transcription. DNA-dependent	GO:0006351	8419	1340	10	8	1.34E-05
RNA Pol III	molecular function	DNA-directed RNA polymerase activity	GO:0003899	9140	29	5	5	2.24E-13
RNA Pol III	molecular function	nucleotidyltransferase activity	GO:0016779	9140	79	5	5	4.24E-11
RNA Pol III	molecular function	transferase activity. transferring phosphorus-containing groups	GO:0016772	9140	728	5	5	3.17E-06
RNA Pol III	molecular function	transferase activity	GO:0016740	9140	1197	5	5	3.82E-05
RNA Pol III	molecular function	DNA binding	GO:0003677	9140	1324	5	5	6.34E-05
RNA Pol III	cellular component	DNA-directed RNA polymerase III complex	GO:0005666	7684	4	5	3	5.291E-10
RNA Pol III	cellular component	RNA polymerase complex	GO:0030880	7684	19	5	3	1.278E-07
RNA Pol III	biological process	transcription from Pol III promoter	GO:0006383	8419	19	5	3	9.719E-08
RNA Pol III	biological process	regulation of transcription from Pol III promoter	GO:0006359	8419	6	5	2	4.229E-06
SRP	cellular component	signal recognition particle (sensu Eukaryota)	GO:0005786	7684	8	4	4	4.823E-13
SRP	cellular component	signal recognition particle	GO:0048500	7684	8	4	4	4.823E-13
SRP	biological process	cotranslational protein-membrane targeting	GO:0006613	8419	10	3	2	3.807E-06
SRP	biological process	protein-ER targeting	GO:0045047	8419	10	3	2	3.807E-06
SRP	biological process	protein-membrane targeting	GO:0006612	8419	18	3	2	1.294E-05
SRS	molecular function	structural constituent of ribosome	GO:0003735	9140	140	20	20	1.241E-37
SRS	molecular function	structural molecule activity	GO:0005198	9140	473	20	20	1.29E-26
SRS	molecular function	RNA binding	GO:0003723	9140	415	20	7	1.753E-05
SRS	cellular component	ribosome	GO:0005840	7684	119	20	18	1.282E-31
SRS	cellular component	ribonucleoprotein complex	GO:0030529	7684	251	20	20	9.013E-31
SRS	cellular component	small ribosomal subunit	GO:0015935	7684	31	20	9	1.285E-17
SRS	cellular component	cytosolic small ribosomal subunit (sensu Eukaryota)	GO:0005843	7684	11	20	7	8.126E-17
SRS	cellular component	eukaryotic 48S initiation complex	GO:0016283	7684	11	20	7	8.126E-17
SRS	cellular component	protein complex	GO:0043234	7684	1274	20	20	2.174E-16

Protein complex	Ontology type	GO Term	GO ID	N	M	n	m	P-value
SRS	cellular component	non-membrane-bound organelle	GO:0043228	7684	893	20	18	1.94E-15
SRS	cellular component	intracellular non-membrane-bound organelle	GO:0043232	7684	893	20	18	1.94E-15
SRS	cellular component	eukaryotic 43S preinitiation complex	GO:0016282	7684	24	20	7	8.359E-14
SRS	cellular component	cytosolic ribosome (sensu Eukaryota)	GO:0005830	7684	26	20	7	1.584E-13
SRS	cellular component	cytoplasm	GO:0005737	7684	2299	20	18	3.452E-08
SRS	cellular component	cytosol	GO:0005829	7684	226	20	7	9.706E-07
SRS	biological process	protein biosynthesis	GO:0006412	8419	400	18	18	1.051E-24
SRS	biological process	macromolecule biosynthesis	GO:0009059	8419	447	18	18	8.093E-24
SRS	biological process	cellular biosynthesis	GO:0044249	8419	706	18	18	3.442E-20
SRS	biological process	biosynthesis	GO:0009058	8419	737	18	18	7.529E-20
SRS	biological process	cellular protein metabolism	GO:0044267	8419	2230	18	18	3.916E-11
SRS	biological process	protein metabolism	GO:0019538	8419	2246	18	18	4.456E-11
SRS	biological process	cellular macromolecule metabolism	GO:0044260	8419	2394	18	18	1.411E-10
SRS	biological process	macromolecule metabolism	GO:0043170	8419	2512	18	18	3.365E-10
SRS	biological process	primary metabolism	GO:0044238	8419	4747	18	18	3.272E-05
SRS	biological process	cellular metabolism	GO:0044237	8419	4914	18	18	6.103E-05
TAFIID	molecular function	general RNA polymerase II transcription factor activity	GO:0016251	9140	29	13	6	9.932E-13
TAFIID	molecular function	transcription regulator activity	GO:0030528	9140	881	13	12	7.126E-12
TAFIID	molecular function	RNA polymerase II transcription factor activity	GO:0003702	9140	164	13	8	1.08E-11
TAFIID	molecular function	DNA binding	GO:0003677	9140	1324	13	12	9.223E-10
TAFIID	molecular function	nucleic acid binding	GO:0003676	9140	2115	13	12	2.355E-07
TAFIID	molecular function	transcription factor activity	GO:0003700	9140	606	13	8	3.411E-07
TAFIID	molecular function	transcription initiation factor activity	GO:0016986	9140	5	13	2	1.863E-05
TAFIID	cellular component	transcription factor TFIID complex	GO:0005669	7684	13	13	10	4.156E-28
TAFIID	cellular component	transcription factor complex	GO:0005667	7684	80	13	11	5.861E-21
TAFIID	cellular component	DNA-directed RNA polymerase II. holoenzyme	GO:0016591	7684	54	13	10	3.427E-20
TAFIID	cellular component	nucleoplasm	GO:0005654	7684	193	13	11	1.412E-16
TAFIID	cellular component	protein complex	GO:0043234	7684	1274	13	11	1.412E-07
TAFIID	cellular component	nucleus	GO:0005634	7684	2551	13	13	5.831E-07
TAFIID	cellular component	membrane-bound organelle	GO:0043227	7684	3750	13	13	8.813E-05
TAFIID	cellular component	intracellular membrane-bound organelle	GO:0043231	7684	3750	13	13	8.813E-05
TAFIID	biological process	transcription initiation	GO:0006352	8419	42	13	8	2.391E-16
TAFIID	biological process	transcription	GO:0006350	8419	1436	13	13	9.885E-11
TAFIID	biological process	transcription. DNA-dependent	GO:0006351	8419	1340	13	12	2.816E-09
TAFIID	biological process	regulation of transcription	GO:0045449	8419	1372	13	12	3.726E-09
TAFIID	biological process	regulation of nucleobase. nucleoside. nucleotide and nucleic acid metabolism	GO:0019219	8419	1398	13	12	4.656E-09
TAFIID	biological process	regulation of metabolism	GO:0019222	8419	1521	13	12	1.265E-08
TAFIID	biological process	nucleobase. nucleoside. nucleotide and nucleic acid metabolism	GO:0006139	8419	2192	13	13	2.461E-08
TAFIID	biological process	regulation of transcription. DNA-dependent	GO:0006355	8419	1292	13	11	6.192E-08
TAFIID	biological process	regulation of physiological process	GO:0050791	8419	1924	13	12	2.03E-07

Protein complex	Ontology type	GO Term	GO ID	N	M	n	m	P-value
TAFIID	biological process	regulation of biological process	GO:0050789	8419	2196	13	12	9.587E-07
TAFIID	biological process	regulation of transcription factor activity	GO:0051090	8419	6	13	2	3.29E-05
VHL	biological process	ubiquitin cycle	GO:0006512	8419	438	4	4	7.231E-06
