# Supplementary material for: A new computational approach to analyze human protein complexes and predict novel protein interactions
Source: Genome Biol. 2007 Dec 4;8(12):R256. doi: 10.1186/gb-2007-8-12-r256 (PMC2246258; doi:10.1186/gb-2007-8-12-r256)
Supplement: Additional data file 10 — Entrez Gene IDs of all components of each protein complex analyzed. [file gb-2007-8-12-r256-S10.pdf]

Protein complex	Entrez Gene ID
AP2	1173
AP2	1175
AP2	160
AP2	161
AP2	163
APC	10393
APC	29945
APC	51433
APC	51434
APC	51529
APC	64682
APC	8697
APC	8881
APC	996
ARC	1452
ARC	1499
ARC	2932
ARC	324
ARC	8312
Arp2-3	10092
Arp2-3	10093
Arp2-3	10094
Arp2-3	10095
Arp2-3	10096
Arp2-3	10097
Arp2-3	10109
ATP_F0	10476
ATP_F0	10632
ATP_F0	267020
ATP_F0	27109
ATP_F0	515
ATP_F0	516
ATP_F0	517
ATP_F0	518
ATP_F0	521
ATP_F0	522
ATP_F0	9551
ATP_F1	506
ATP_F1	509
ATP_F1	513
ATP_F1	514
ATP_F1	539
Centrosome	10121
Centrosome	10142
Centrosome	10426
Centrosome	10540
Centrosome	1069
Centrosome	1070
Centrosome	10733
Centrosome	10806

Protein complex	Entrez Gene ID
MSRS	10240
MSRS	10884
MSRS	23107
MSRS	28957
MSRS	28973
MSRS	51021
MSRS	51023
MSRS	51081
MSRS	51116
MSRS	51373
MSRS	51649
MSRS	51650
MSRS	54460
MSRS	55173
MSRS	56945
MSRS	60488
MSRS	6183
MSRS	63931
MSRS	64432
MSRS	64949
MSRS	64951
MSRS	64960
MSRS	64963
MSRS	64965
MSRS	64968
MSRS	64969
MSRS	65993
MSRS	7818
MSRS	78988
MSRS	92259
Nucleopore	10762
Nucleopore	1104
Nucleopore	22981
Nucleopore	23165
Nucleopore	23225
Nucleopore	23279
Nucleopore	23511
Nucleopore	23636
Nucleopore	3185
Nucleopore	3187
Nucleopore	3188
Nucleopore	3837
Nucleopore	3838
Nucleopore	4000
Nucleopore	4670
Nucleopore	4927
Nucleopore	4928
Nucleopore	53371
Nucleopore	55746
Nucleopore	57122

Protein complex	Entrez Gene ID
Centrosome	10844
Centrosome	11064
Centrosome	11190
Centrosome	11258
Centrosome	114791
Centrosome	1453
Centrosome	1454
Centrosome	1639
Centrosome	1778
Centrosome	1781
Centrosome	203068
Centrosome	22897
Centrosome	22919
Centrosome	22994
Centrosome	22995
Centrosome	23177
Centrosome	23332
Centrosome	4751
Centrosome	4957
Centrosome	5048
Centrosome	50946
Centrosome	51143
Centrosome	5116
Centrosome	51199
Centrosome	5347
Centrosome	54820
Centrosome	55125
Centrosome	55142
Centrosome	5566
Centrosome	55722
Centrosome	55755
Centrosome	5576
Centrosome	5577
Centrosome	55835
Centrosome	7277
Centrosome	7283
Centrosome	7531
Centrosome	7532
Centrosome	7840
Centrosome	7846
Centrosome	79959
Centrosome	80086
Centrosome	80184
Centrosome	80254
Centrosome	80321
Centrosome	84131
Centrosome	8481
Centrosome	85378
Centrosome	8636
Centrosome	8655

Protein complex	Entrez Gene ID
Nucleopore	5901
Nucleopore	5905
Nucleopore	5906
Nucleopore	59343
Nucleopore	6396
Nucleopore	7329
Nucleopore	7431
Nucleopore	8021
Nucleopore	8480
Nucleopore	84823
Nucleopore	9631
Nucleopore	9688
Nucleopore	9972
Nucleosome	3005
Nucleosome	3006
Nucleosome	3012
Nucleosome	3014
Nucleosome	3015
Nucleosome	3017
Nucleosome	3020
Nucleosome	3021
Nucleosome	3024
Nucleosome	55766
Nucleosome	8334
Nucleosome	8336
Nucleosome	8337
Nucleosome	8340
Nucleosome	8343
Nucleosome	8347
Nucleosome	8349
Nucleosome	8351
Nucleosome	8363
Nucleosome	8364
Nucleosome	8365
Nucleosome	8366
Nucleosome	8367
Nucleosome	8370
Nucleosome	83740
Nucleosome	85236
Nucleosome	8969
Nucleosome	8970
Nucleosome	8971
Nucleosome	92815
Nucleosome	94239
Nucleosome	9555
ORC	23594
ORC	23595
ORC	4998
ORC	4999
ORC	5000

Protein complex	Entrez Gene ID
Centrosome	95681
Centrosome	9662
Centrosome	9696
Centrosome	9702
Centrosome	9738
Centrosome	9793
COX	1327
COX	1329
COX	1337
COX	1339
COX	1340
COX	1349
COX	1350
COX	1351
COX	170712
COX	84701
COX	9377
Dynactin	10121
Dynactin	10540
Dynactin	10671
Dynactin	11258
Dynactin	1639
Dynactin	51164
Dynactin	55860
Dynactin	829
Dynactin	830
Dynactin	832
Dynactin	93661
Exocyst	10640
Exocyst	11336
Exocyst	149371
Exocyst	23233
Exocyst	23265
Exocyst	54536
Exocyst	55763
Exocyst	55770
Exocyst	60412
Exosome	23016
Exosome	23404
Exosome	51010
Exosome	51013
Exosome	5393
Exosome	5394
Exosome	54512
Exosome	56915
FA	10174
FA	10580
FA	1396
FA	1397
FA	140885

Protein complex	Entrez Gene ID
ORC	5001
PD	1737
PD	1738
PD	300502
PD	5162
PD	5163
PD	5164
PD	5165
PD	8050
Proteasome	10213
Proteasome	5682
Proteasome	5683
Proteasome	5684
Proteasome	5685
Proteasome	5686
Proteasome	5687
Proteasome	5688
Proteasome	5689
Proteasome	5690
Proteasome	5691
Proteasome	5692
Proteasome	5693
Proteasome	5694
Proteasome	5695
Proteasome	5700
Proteasome	5701
Proteasome	5702
Proteasome	5704
Proteasome	5705
Proteasome	5706
Proteasome	5707
Proteasome	5708
Proteasome	5709
Proteasome	5710
Proteasome	5713
Proteasome	5714
Proteasome	5717
Proteasome	5718
Proteasome	5719
Proteasome	9861
RFC	5981
RFC	5982
RFC	5983
RFC	5984
RFC	5985
RNA Pol II	5430
RNA Pol II	5431
RNA Pol II	5432
RNA Pol II	5433
RNA Pol II	5434

Protein complex	Entrez Gene ID
FA	143903
FA	1445
FA	2119
FA	2274
FA	23022
FA	2316
FA	2317
FA	2318
FA	23683
FA	25
FA	27111
FA	29780
FA	3611
FA	3636
FA	3987
FA	4478
FA	5058
FA	5062
FA	5063
FA	50807
FA	5329
FA	5358
FA	55742
FA	5578
FA	5582
FA	5583
FA	5590
FA	5747
FA	5781
FA	5792
FA	5829
FA	6385
FA	6386
FA	64098
FA	6714
FA	7094
FA	7145
FA	7408
FA	7414
FA	7791
FA	81
FA	824
FA	83660
FA	84309
FA	857
FA	858
FA	859
FA	87
FA	9459
GTC	10466

Protein complex	Entrez Gene ID
RNA Pol II	5435
RNA Pol II	5436
RNA Pol II	5437
RNA Pol II	5438
RNA Pol II	5439
RNA Pol II	5440
RNA Pol II	5441
RNA Pol III	10621
RNA Pol III	10622
RNA Pol III	10623
RNA Pol III	11128
RNA Pol III	51082
RNA Pol III	51728
RNA Pol III	55703
RNA Pol III	661
RNA Pol III	9533
SCF	6500
SCF	8454
SCF	9978
SNARE	10282
SNARE	10490
SNARE	10652
SNARE	26984
SNARE	6811
SNARE	9527
SNARE	9570
SRP	6726
SRP	6727
SRP	6728
SRP	6729
SRP	6730
SRP	6731
SRS	2197
SRS	3921
SRS	51065
SRS	6187
SRS	6188
SRS	6189
SRS	6191
SRS	6193
SRS	6194
SRS	6201
SRS	6202
SRS	6203
SRS	6204
SRS	6205
SRS	6206
SRS	6207
SRS	6208
SRS	6209

Protein complex	Entrez Gene ID
GTC	22796
GTC	25839
GTC	57511
GTC	83548
GTC	84342
GTC	91949
GTC	9382
LRS	11224
LRS	23521
LRS	25873
LRS	4736
LRS	6122
LRS	6124
LRS	6125
LRS	6128
LRS	6129
LRS	6130
LRS	6132
LRS	6133
LRS	6135
LRS	6136
LRS	6137
LRS	6138
LRS	6139
LRS	6141
LRS	6142
LRS	6143
LRS	6144
LRS	6146
LRS	6147
LRS	6155
LRS	6156
LRS	6157
LRS	6158
LRS	6159
LRS	6160
LRS	6161
LRS	6164
LRS	6165
LRS	6167
LRS	6168
LRS	6169
LRS	6170
LRS	6171
LRS	6173
LRS	6175
LRS	6176
LRS	7311
LRS	9045
LRS	9349

Protein complex	Entrez Gene ID
SRS	6210
SRS	6217
SRS	6218
SRS	6222
SRS	6223
SRS	6224
SRS	6227
SRS	6228
SRS	6229
SRS	6230
SRS	6231
SRS	6233
SRS	6234
SRS	6235
SWI/SNF	51412
SWI/SNF	55193
SWI/SNF	6595
SWI/SNF	6597
SWI/SNF	6598
SWI/SNF	6599
SWI/SNF	6601
SWI/SNF	6602
SWI/SNF	6603
SWI/SNF	6604
SWI/SNF	6605
SWI/SNF	8289
SWI/SNF	86
TAFIID	10629
TAFIID	138474
TAFIID	27097
TAFIID	51616
TAFIID	54457
TAFIID	6872
TAFIID	6873
TAFIID	6874
TAFIID	6877
TAFIID	6878
TAFIID	6879
TAFIID	6880
TAFIID	6881
TAFIID	6882
TAFIID	6883
TAFIID	6884
TAFIID	6908
TRAPP	122553
TRAPP	126003
TRAPP	27095
TRAPP	51399
TRAPP	58485
TRAPP	6399

Protein complex	Entrez Gene ID
MLRS	10573
MLRS	11222
MLRS	114294
MLRS	116540
MLRS	116541
MLRS	122704
MLRS	124995
MLRS	128308
MLRS	219927
MLRS	26589
MLRS	28977
MLRS	28998
MLRS	29074
MLRS	29088
MLRS	29093
MLRS	51069
MLRS	51073
MLRS	51253
MLRS	51258
MLRS	51263
MLRS	51264
MLRS	51318
MLRS	51642
MLRS	54148
MLRS	54534
MLRS	54948
MLRS	55052
MLRS	57129
MLRS	6150
MLRS	6182
MLRS	63875
MLRS	64928
MLRS	64975
MLRS	64976
MLRS	64978
MLRS	64979
MLRS	64981
MLRS	64983
MLRS	65003
MLRS	65005
MLRS	65008
MLRS	65080
MLRS	740
MLRS	79590
MLRS	84311
MLRS	84545
MLRS	9553
MLRS	9801

Protein complex	Entrez Gene ID
TRAPP	79090
VHL	6921
VHL	6923
VHL	7428
VHL	8453
VHL	9978
