# Supplementary material for: A new computational approach to analyze human protein complexes and predict novel protein interactions
Source: Genome Biol. 2007 Dec 4;8(12):R256. doi: 10.1186/gb-2007-8-12-r256 (PMC2246258; doi:10.1186/gb-2007-8-12-r256)
Supplement: Additional data file 11 — For each gene, indicated by its Entrez Gene ID, the corresponding IMAGE IDs present in each dataset is reported. (a) Thy-Thy2; (b) Thy-Thy3; (c) Thy-Noc. [file gb-2007-8-12-r256-S11.pdf]

A

Protein complex	Gene ID	Clone ID
AP2	1175	IMAGE:739109
AP2	163	IMAGE:245853
AP2	163	IMAGE:416340
APC	10393	IMAGE:966335
APC	51434	IMAGE:131110
APC	51434	IMAGE:66919
APC	64682	IMAGE:45771
APC	64682	IMAGE:511267
APC	64682	IMAGE:731433
APC	8881	IMAGE:197779
APC	8881	IMAGE:755385
APC	996	IMAGE:109708
APC	996	IMAGE:842946
ARC	1452	IMAGE:745402
ARC	1499	IMAGE:42816
ARC	1499	IMAGE:774754
ARC	2932	IMAGE:197128
ARC	2932	IMAGE:38883
ARC	2932	IMAGE:768520
ARC	324	IMAGE:38028
ARC	324	IMAGE:429926
ARC	324	IMAGE:812083
Arp2-3	10092	IMAGE:340558
Arp2-3	10093	IMAGE:1456962
Arp2-3	10094	IMAGE:1473922
Arp2-3	10094	IMAGE:232826
Arp2-3	10096	IMAGE:271568
Arp2-3	10096	IMAGE:593251
Arp2-3	10096	IMAGE:813543
Arp2-3	10097	IMAGE:1584391
Arp2-3	10109	IMAGE:162208
ATP_F0	27109	IMAGE:198190
ATP_F0	27109	IMAGE:254533
ATP_F0	27109	IMAGE:321163
ATP_F0	515	IMAGE:813712
ATP_F0	516	IMAGE:487373
ATP_F0	517	IMAGE:809876
ATP_F0	518	IMAGE:193106
ATP_F0	518	IMAGE:611150
ATP_F0	521	IMAGE:782439
ATP_F0	522	IMAGE:139199
ATP_F0	522	IMAGE:825312
ATP_F1	506	IMAGE:392622
ATP_F1	509	IMAGE:845519
ATP_F1	513	IMAGE:856650
ATP_F1	539	IMAGE:1472150
Centrosome	10121	IMAGE:342342
Centrosome	10121	IMAGE:815575

Protein complex	Gene ID	Clone ID
Centrosome	10426	IMAGE:1029186
Centrosome	10426	IMAGE:244955
Centrosome	10426	IMAGE:950746
Centrosome	10540	IMAGE:725335
Centrosome	1069	IMAGE:291216
Centrosome	1070	IMAGE:487425
Centrosome	10733	1343971
Centrosome	10806	IMAGE:417043
Centrosome	11064	IMAGE:210610
Centrosome	11064	IMAGE:430192
Centrosome	11190	IMAGE:165921
Centrosome	1454	IMAGE:854138
Centrosome	1639	IMAGE:877613
Centrosome	1778	IMAGE:415406
Centrosome	1781	IMAGE:206457
Centrosome	1781	IMAGE:243360
Centrosome	1781	IMAGE:294136
Centrosome	1781	IMAGE:809714
Centrosome	203068	IMAGE:191603
Centrosome	22897	IMAGE:122443
Centrosome	23177	IMAGE:796747
Centrosome	23332	IMAGE:41029
Centrosome	4751	IMAGE:462926
Centrosome	4957	IMAGE:504469
Centrosome	4957	IMAGE:743278
Centrosome	5048	IMAGE:245883
Centrosome	5048	IMAGE:878178
Centrosome	5116	IMAGE:283341
Centrosome	51199	IMAGE:129502
Centrosome	51199	IMAGE:271357
Centrosome	5347	IMAGE:744047
Centrosome	5566	IMAGE:246705
Centrosome	55722	IMAGE:503824
Centrosome	55755	IMAGE:110741
Centrosome	55755	IMAGE:241343
Centrosome	5576	IMAGE:743739
Centrosome	5577	IMAGE:609663
Centrosome	5577	IMAGE:753286
Centrosome	55835	IMAGE:786546
Centrosome	7283	IMAGE:108377
Centrosome	7532	IMAGE:127931
Centrosome	7532	IMAGE:784129
Centrosome	7840	IMAGE:198011
Centrosome	7846	IMAGE:1470060
Centrosome	79959	IMAGE:1031017
Centrosome	80254	IMAGE:273648
Centrosome	80254	IMAGE:427897
Centrosome	80321	IMAGE:256680
Centrosome	8481	IMAGE:246703

Protein complex	Gene ID	Clone ID
Centrosome	8655	IMAGE:853938
Centrosome	95681	IMAGE:1636205
Centrosome	95681	IMAGE:43009
Centrosome	95681	IMAGE:772408
Centrosome	9702	IMAGE:489662
Centrosome	9702	IMAGE:665082
Centrosome	9738	IMAGE:745339
Centrosome	9793	IMAGE:898032
COX	1329	IMAGE:143145
COX	1337	IMAGE:840894
COX	1340	IMAGE:298965
COX	1340	IMAGE:512003
COX	1349	IMAGE:884511
COX	1350	IMAGE:111924
COX	1350	IMAGE:884480
COX	1351	IMAGE:1469230
COX	9377	IMAGE:824068
Dynactin	10121	IMAGE:342342
Dynactin	10121	IMAGE:815575
Dynactin	10540	IMAGE:725335
Dynactin	10671	IMAGE:788745
Dynactin	1639	IMAGE:877613
Dynactin	51164	IMAGE:877636
Dynactin	829	IMAGE:1031582
Dynactin	829	IMAGE:785793
Dynactin	832	IMAGE:1861608
Dynactin	832	IMAGE:1862179
Dynactin	832	IMAGE:322961
Dynactin	832	IMAGE:769911
Exocyst	10640	IMAGE:144905
Exocyst	23233	IMAGE:258063
Exocyst	23265	IMAGE:159166
Exocyst	54536	IMAGE:625764
Exocyst	55763	IMAGE:594428
Exocyst	55770	IMAGE:230316
Exocyst	55770	IMAGE:813195
Exocyst	60412	IMAGE:199327
Exocyst	60412	IMAGE:251212
Exocyst	60412	IMAGE:916505
Exocyst	60412	IMAGE:997280
Exosome	23016	IMAGE:1475987
Exosome	5394	IMAGE:841179
Exosome	54512	IMAGE:1630936
FA	10174	IMAGE:453183
FA	1396	IMAGE:1323448
FA	1397	IMAGE:811046
FA	140885	IMAGE:731118
FA	1445	IMAGE:526282
FA	2119	IMAGE:796542

Protein complex	Gene ID	Clone ID
FA	2316	IMAGE:898281
FA	2317	IMAGE:243652
FA	2317	IMAGE:840818
FA	23683	IMAGE:752701
FA	23683	IMAGE:796932
FA	25	IMAGE:123858
FA	25	IMAGE:219976
FA	25	IMAGE:897642
FA	3636	IMAGE:703964
FA	3987	IMAGE:365004
FA	3987	IMAGE:825416
FA	4478	IMAGE:131362
FA	5058	IMAGE:1405689
FA	5058	IMAGE:53110
FA	5058	IMAGE:595200
FA	5062	IMAGE:134439
FA	5062	IMAGE:173561
FA	5063	IMAGE:132217
FA	5329	IMAGE:590154
FA	5329	IMAGE:810017
FA	5358	IMAGE:965085
FA	5578	IMAGE:297136
FA	5578	IMAGE:469954
FA	5578	IMAGE:768246
FA	5582	IMAGE:167032
FA	5583	IMAGE:380245
FA	5590	IMAGE:120863
FA	5590	IMAGE:131239
FA	5590	IMAGE:167280
FA	5590	IMAGE:814266
FA	5747	IMAGE:383706
FA	5747	IMAGE:724892
FA	5747	IMAGE:855864
FA	5781	IMAGE:34773
FA	5781	IMAGE:814776
FA	5792	IMAGE:897788
FA	5829	IMAGE:770080
FA	6385	IMAGE:504763
FA	6386	IMAGE:813533
FA	7408	IMAGE:753418
FA	7414	IMAGE:841203
FA	81	IMAGE:213535
FA	824	IMAGE:549728
FA	83660	IMAGE:416409
FA	83660	IMAGE:490970
FA	857	IMAGE:377461
FA	857	IMAGE:841664
FA	858	IMAGE:110467
FA	858	IMAGE:840984

Protein complex	Gene ID	Clone ID
FA	859	IMAGE:773276
FA	9459	IMAGE:687990
GTC	22796	IMAGE:825296
GTC	25839	IMAGE:1010089
GTC	25839	IMAGE:1010093
GTC	57511	IMAGE:268780
GTC	57511	IMAGE:343352
GTC	91949	IMAGE:204536
GTC	91949	IMAGE:40010
GTC	9382	IMAGE:125608
GTC	9382	IMAGE:126277
GTC	9382	IMAGE:194023
GTC	9382	IMAGE:289637
GTC	9382	IMAGE:356711
LRS	11224	IMAGE:877835
LRS	23521	IMAGE:1962510
LRS	4736	IMAGE:124824
LRS	6125	IMAGE:897596
LRS	6128	IMAGE:884546
LRS	6130	IMAGE:51981
LRS	6135	IMAGE:1220403
LRS	6135	IMAGE:869450
LRS	6138	IMAGE:837904
LRS	6141	IMAGE:878545
LRS	6158	IMAGE:841044
LRS	6165	IMAGE:1471829
LRS	6167	IMAGE:344975
LRS	7311	IMAGE:1492412
LRS	9045	IMAGE:1861366
LRS	9045	IMAGE:1862035
LRS	9349	IMAGE:796885
MLRS	11222	IMAGE:44255
MLRS	114294	IMAGE:665650
MLRS	128308	IMAGE:292232
MLRS	219927	IMAGE:809517
MLRS	26589	IMAGE:784214
MLRS	28977	IMAGE:203114
MLRS	28977	IMAGE:277163
MLRS	29074	IMAGE:796281
MLRS	51069	IMAGE:309494
MLRS	51073	IMAGE:824568
MLRS	51253	IMAGE:843263
MLRS	51263	IMAGE:1584573
MLRS	51263	IMAGE:85318
MLRS	51318	IMAGE:417208
MLRS	51318	IMAGE:490147
MLRS	54948	IMAGE:126239
MLRS	6150	IMAGE:788334
MLRS	64978	IMAGE:809738

Protein complex	Gene ID	Clone ID
MLRS	64979	IMAGE:811867
MLRS	64983	IMAGE:291633
MLRS	65008	IMAGE:321359
MLRS	740	IMAGE:324885
MLRS	84545	IMAGE:376214
MLRS	9553	IMAGE:325520
MLRS	9553	IMAGE:897448
MLRS	9801	IMAGE:563701
MLRS	9801	IMAGE:827144
MSRS	10884	IMAGE:292469
MSRS	23107	IMAGE:843133
MSRS	28957	IMAGE:768961
MSRS	28957	IMAGE:773483
MSRS	28973	IMAGE:592491
MSRS	28973	IMAGE:812988
MSRS	51023	IMAGE:294304
MSRS	51081	IMAGE:564625
MSRS	51116	IMAGE:810979
MSRS	51650	IMAGE:127396
MSRS	54460	IMAGE:771173
MSRS	55173	IMAGE:810753
MSRS	60488	IMAGE:246041
MSRS	6183	IMAGE:131653
MSRS	63931	IMAGE:796255
MSRS	64432	IMAGE:1007835
MSRS	64432	IMAGE:272632
MSRS	64432	IMAGE:299468
MSRS	64432	IMAGE:44409
MSRS	64949	IMAGE:810027
MSRS	64951	IMAGE:811770
MSRS	64963	IMAGE:271672
MSRS	64965	IMAGE:279691
MSRS	64968	IMAGE:345743
MSRS	64968	IMAGE:744360
MSRS	64969	IMAGE:812169
Nucleopore	10762	IMAGE:730361
Nucleopore	22981	IMAGE:647842
Nucleopore	23165	IMAGE:950369
Nucleopore	23225	IMAGE:122782
Nucleopore	23279	IMAGE:33299
Nucleopore	23279	IMAGE:565758
Nucleopore	3187	IMAGE:129185
Nucleopore	3187	IMAGE:195127
Nucleopore	3187	IMAGE:358457
Nucleopore	3188	IMAGE:866874
Nucleopore	3837	IMAGE:564537
Nucleopore	3837	IMAGE:684634
Nucleopore	3837	IMAGE:768347
Nucleopore	3838	IMAGE:882510

Protein complex	Gene ID	Clone ID
Nucleopore	4000	IMAGE:897544
Nucleopore	4670	IMAGE:123761
Nucleopore	4670	IMAGE:825411
Nucleopore	4927	IMAGE:843070
Nucleopore	53371	IMAGE:287122
Nucleopore	55746	IMAGE:34345
Nucleopore	55746	IMAGE:53265
Nucleopore	55746	IMAGE:627086
Nucleopore	5901	IMAGE:811956
Nucleopore	5905	IMAGE:122019
Nucleopore	5905	IMAGE:811150
Nucleopore	5906	IMAGE:704905
Nucleopore	59343	IMAGE:627112
Nucleopore	6396	IMAGE:897636
Nucleopore	7329	IMAGE:841292
Nucleopore	7431	IMAGE:590323
Nucleopore	7431	IMAGE:840511
Nucleopore	8021	IMAGE:194353
Nucleopore	8021	IMAGE:743188
Nucleopore	8480	IMAGE:825224
Nucleopore	84823	IMAGE:815501
Nucleopore	9631	IMAGE:130371
Nucleopore	9688	IMAGE:283086
Nucleopore	9688	IMAGE:51918
Nucleopore	9688	IMAGE:769751
Nucleopore	9972	IMAGE:27548
Nucleosome	3005	IMAGE:205445
Nucleosome	3005	IMAGE:343744
Nucleosome	3006	IMAGE:66317
Nucleosome	3014	IMAGE:256664
Nucleosome	3017	IMAGE:243784
Nucleosome	3020	IMAGE:884272
Nucleosome	3021	IMAGE:950574
Nucleosome	55766	IMAGE:289734
Nucleosome	8334	IMAGE:124128
Nucleosome	8334	IMAGE:241274
Nucleosome	8334	IMAGE:283919
Nucleosome	8334	IMAGE:789091
Nucleosome	8337	IMAGE:130004
Nucleosome	8337	IMAGE:754628
Nucleosome	8349	IMAGE:430235
Nucleosome	8349	IMAGE:813149
Nucleosome	8364	IMAGE:1461138
Nucleosome	83740	IMAGE:450375
Nucleosome	85236	IMAGE:290841
Nucleosome	8971	IMAGE:347560
Nucleosome	92815	IMAGE:838774
Nucleosome	94239	IMAGE:249949
Nucleosome	9555	IMAGE:843075

Protein complex	Gene ID	Clone ID
ORC	23594	IMAGE:199024
ORC	23595	IMAGE:260336
ORC	4998	IMAGE:121341
ORC	4998	IMAGE:194236
ORC	5000	IMAGE:1535410
PD	1737	IMAGE:124252
PD	1737	IMAGE:271006
PD	1738	IMAGE:813648
PD	5162	IMAGE:826077
PD	5164	IMAGE:49860
PD	5165	IMAGE:278242
PD	8050	IMAGE:279665
Proteasome	5682	IMAGE:134544
Proteasome	5682	IMAGE:298775
Proteasome	5683	IMAGE:155434
Proteasome	5684	IMAGE:814246
Proteasome	5685	IMAGE:399536
Proteasome	5685	IMAGE:531862
Proteasome	5686	IMAGE:897952
Proteasome	5687	IMAGE:509495
Proteasome	5688	IMAGE:1455641
Proteasome	5689	IMAGE:82131
Proteasome	5690	IMAGE:122241
Proteasome	5690	IMAGE:593838
Proteasome	5690	IMAGE:731223
Proteasome	5691	IMAGE:951233
Proteasome	5693	IMAGE:1460110
Proteasome	5694	IMAGE:529861
Proteasome	5695	IMAGE:843352
Proteasome	5701	IMAGE:684655
Proteasome	5704	IMAGE:756157
Proteasome	5704	IMAGE:810558
Proteasome	5706	IMAGE:198614
Proteasome	5706	IMAGE:767049
Proteasome	5706	IMAGE:771900
Proteasome	5709	IMAGE:66377
Proteasome	5709	IMAGE:815861
Proteasome	5714	IMAGE:810550
Proteasome	5717	IMAGE:179443
Proteasome	5717	IMAGE:360772
Proteasome	5717	IMAGE:383945
Proteasome	5717	IMAGE:566474
Proteasome	5718	IMAGE:823598
Proteasome	9861	IMAGE:122237
RFC	5981	IMAGE:214537
RFC	5981	IMAGE:289787
RFC	5983	IMAGE:256260
RFC	5984	IMAGE:203275
RFC	5984	IMAGE:309288

Protein complex	Gene ID	Clone ID
RNA Pol II	5430	IMAGE:430236
RNA Pol II	5430	IMAGE:740130
RNA Pol II	5431	IMAGE:121580
RNA Pol II	5431	IMAGE:295551
RNA Pol II	5432	IMAGE:770391
RNA Pol II	5433	IMAGE:49548
RNA Pol II	5434	IMAGE:469369
RNA Pol II	5435	IMAGE:767817
RNA Pol II	5436	IMAGE:109269
RNA Pol II	5436	IMAGE:740672
RNA Pol II	5438	IMAGE:283615
RNA Pol II	5438	IMAGE:378502
RNA Pol II	5440	IMAGE:813410
RNA Pol II	5441	IMAGE:1325816
RNA Pol III	10623	IMAGE:711959
RNA Pol III	10623	IMAGE:839092
RNA Pol III	11128	IMAGE:810943
RNA Pol III	51728	IMAGE:511632
RNA Pol III	9533	IMAGE:399532
SCF	6500	IMAGE:291464
SCF	8454	IMAGE:841093
SCF	9978	IMAGE:200144
SCF	9978	IMAGE:433666
SNARE	10282	IMAGE:203351
SNARE	10490	IMAGE:451098
SNARE	10652	IMAGE:810762
SNARE	26984	IMAGE:324745
SNARE	6811	IMAGE:787857
SNARE	9527	IMAGE:625863
SNARE	9570	IMAGE:130773
SNARE	9570	IMAGE:1943138
SNARE	9570	IMAGE:239708
SNARE	9570	IMAGE:66391
SNARE	9570	IMAGE:841697
SRP	6728	IMAGE:754998
SRP	6730	IMAGE:626358
SRP	6730	IMAGE:814791
SRP	6731	IMAGE:321510
SRP	6731	IMAGE:811842
SRS	2197	IMAGE:1985178
SRS	51065	IMAGE:590338
SRS	6187	IMAGE:429128
SRS	6191	IMAGE:1492147
SRS	6193	IMAGE:809578
SRS	6194	IMAGE:303048
SRS	6202	IMAGE:62257
SRS	6202	IMAGE:971367
SRS	6203	IMAGE:1861627
SRS	6204	IMAGE:111136

Protein complex	Gene ID	Clone ID
SRS	6204	IMAGE:139641
SRS	6204	IMAGE:1636549
SRS	6204	IMAGE:251195
SRS	6204	IMAGE:780997
SRS	6207	IMAGE:857243
SRS	6208	IMAGE:741522
SRS	6208	IMAGE:756820
SRS	6217	IMAGE:853151
SRS	6223	IMAGE:192242
SRS	6223	IMAGE:1983719
SRS	6224	IMAGE:1699381
SRS	6224	IMAGE:2007305
SRS	6224	IMAGE:80265
SRS	6228	IMAGE:291974
SRS	6228	IMAGE:511882
SRS	6228	IMAGE:868308
SRS	6230	IMAGE:1475738
SWI-SNF	51412	IMAGE:113394
SWI-SNF	55193	IMAGE:214744
SWI-SNF	55193	IMAGE:239615
SWI-SNF	55193	IMAGE:296883
SWI-SNF	55193	IMAGE:427811
SWI-SNF	55193	IMAGE:811981
SWI-SNF	55193	IMAGE:813644
SWI-SNF	6595	IMAGE:814636
SWI-SNF	6597	IMAGE:1011375
SWI-SNF	6598	IMAGE:781018
SWI-SNF	6598	IMAGE:80271
SWI-SNF	6599	IMAGE:1475797
SWI-SNF	6601	IMAGE:160838
SWI-SNF	6602	IMAGE:241736
SWI-SNF	6603	IMAGE:741067
SWI-SNF	6604	IMAGE:360047
SWI-SNF	8289	IMAGE:810843
SWI-SNF	86	IMAGE:753400
TAFIID	6872	IMAGE:454440
TAFIID	6873	IMAGE:136730
TAFIID	6878	IMAGE:33438
TAFIID	6879	IMAGE:242700
TAFIID	6879	IMAGE:365930
TAFIID	6881	IMAGE:723972
TAFIID	6882	IMAGE:306444
TAFIID	6883	IMAGE:509588
TAFIID	6908	IMAGE:280735
TRAPP	27095	IMAGE:780977
TRAPP	58485	IMAGE:731014
VHL	6923	IMAGE:884692
VHL	7428	IMAGE:234856

VHL	8453	IMAGE:788247
VHL	9978	IMAGE:200144
VHL	9978	IMAGE:433666

B

Protein complex	Gene ID	Clone ID
AP2	1173	IMAGE:1846645
AP2	1175	IMAGE:739109
AP2	161	IMAGE:1469115
AP2	161	IMAGE:1631984
AP2	163	IMAGE:245853
AP2	163	IMAGE:416340
APC	10393	IMAGE:2114111
APC	10393	IMAGE:270932
APC	10393	IMAGE:966335
APC	29945	IMAGE:754377
APC	51433	IMAGE:823954
APC	51434	IMAGE:131110
APC	51434	IMAGE:66919
APC	51434	IMAGE:786096
APC	64682	IMAGE:1534603
APC	64682	IMAGE:45771
APC	64682	IMAGE:511267
APC	64682	IMAGE:731433
APC	8697	IMAGE:1871423
APC	8697	IMAGE:240969
APC	8697	IMAGE:26462
APC	8881	IMAGE:1506477
APC	8881	IMAGE:197779
APC	8881	IMAGE:755385
APC	996	IMAGE:109708
APC	996	IMAGE:281585
APC	996	IMAGE:504396
APC	996	IMAGE:75639
APC	996	IMAGE:842946
APC	996	IMAGE:869458
ARC	1452	IMAGE:433490
ARC	1452	IMAGE:745402
ARC	1499	IMAGE:1900880
ARC	1499	IMAGE:774754
ARC	2932	IMAGE:197128
ARC	324	IMAGE:1742727
ARC	324	IMAGE:206838
ARC	324	IMAGE:269332
ARC	324	IMAGE:429926
ARC	324	IMAGE:812083
ARC	8312	IMAGE:1461733
ARC	8312	IMAGE:1506605
ARC	8312	IMAGE:1754823
Arp2-3	10092	IMAGE:340558
Arp2-3	10094	IMAGE:232826

Protein complex	Gene ID	Clone ID
Arp2-3	10096	1293112
Arp2-3	10096	IMAGE:194587
Arp2-3	10096	IMAGE:271568
Arp2-3	10096	IMAGE:593251
Arp2-3	10096	IMAGE:813543
ATP_F0	10476	IMAGE:825386
ATP_F0	267020	IMAGE:461264
ATP_F0	27109	IMAGE:198190
ATP_F0	27109	IMAGE:254533
ATP_F0	27109	IMAGE:379343
ATP_F0	515	IMAGE:813712
ATP_F0	516	IMAGE:487373
ATP_F0	517	IMAGE:809876
ATP_F0	518	IMAGE:193106
ATP_F0	518	IMAGE:611150
ATP_F0	521	IMAGE:782439
ATP_F0	522	1048899
ATP_F0	522	IMAGE:139199
ATP_F0	522	IMAGE:564558
ATP_F0	522	IMAGE:825312
ATP_F0	9551	IMAGE:1574131
ATP_F1	509	IMAGE:845519
ATP_F1	513	IMAGE:856650
ATP_F1	514	IMAGE:434968
Centrosome	10121	IMAGE:453109
Centrosome	10142	IMAGE:197672
Centrosome	10142	IMAGE:2306682
Centrosome	10142	IMAGE:40263
Centrosome	10426	IMAGE:1029186
Centrosome	10426	IMAGE:244955
Centrosome	10426	IMAGE:950746
Centrosome	10426	IMAGE:964784
Centrosome	10540	IMAGE:1849203
Centrosome	10540	IMAGE:725335
Centrosome	1070	IMAGE:487425
Centrosome	10733	IMAGE:2106955
Centrosome	10806	IMAGE:1691128
Centrosome	10806	IMAGE:248654
Centrosome	10806	IMAGE:417043
Centrosome	10806	IMAGE:785880
Centrosome	10844	IMAGE:453348
Centrosome	11064	IMAGE:210610
Centrosome	11064	IMAGE:430192
Centrosome	11190	IMAGE:165921
Centrosome	11190	IMAGE:1674822
Centrosome	1454	IMAGE:854138
Centrosome	1454	IMAGE:878111
Centrosome	1639	IMAGE:877613
Centrosome	1778	IMAGE:251618

Protein complex	Gene ID	Clone ID
Centrosome	1778	IMAGE:415406
Centrosome	1781	IMAGE:1646552
Centrosome	1781	IMAGE:206457
Centrosome	1781	IMAGE:243360
Centrosome	1781	IMAGE:294136
Centrosome	1781	IMAGE:809714
Centrosome	1781	IMAGE:843429
Centrosome	22897	IMAGE:122443
Centrosome	22919	IMAGE:428223
Centrosome	22995	IMAGE:1941598
Centrosome	22995	IMAGE:296334
Centrosome	23177	IMAGE:1679826
Centrosome	23177	IMAGE:1754869
Centrosome	23177	IMAGE:1878424
Centrosome	23177	IMAGE:1878917
Centrosome	23177	IMAGE:1893101
Centrosome	23177	IMAGE:1893445
Centrosome	23177	IMAGE:488160
Centrosome	23177	IMAGE:796747
Centrosome	23332	IMAGE:127102
Centrosome	23332	IMAGE:1901509
Centrosome	23332	IMAGE:41029
Centrosome	4751	IMAGE:198104
Centrosome	4751	IMAGE:415089
Centrosome	4957	IMAGE:504469
Centrosome	4957	IMAGE:743278
Centrosome	5048	IMAGE:212320
Centrosome	5048	IMAGE:245883
Centrosome	5048	IMAGE:878178
Centrosome	51199	IMAGE:129502
Centrosome	51199	IMAGE:1619448
Centrosome	51199	IMAGE:1935464
Centrosome	51199	IMAGE:197399
Centrosome	51199	IMAGE:271357
Centrosome	51199	IMAGE:430751
Centrosome	51199	IMAGE:683361
Centrosome	51199	IMAGE:815251
Centrosome	5347	IMAGE:744047
Centrosome	54820	IMAGE:810947
Centrosome	55125	IMAGE:434782
Centrosome	55142	IMAGE:1849940
Centrosome	55142	IMAGE:703569
Centrosome	5566	IMAGE:246705
Centrosome	5566	IMAGE:342543
Centrosome	55722	IMAGE:503824
Centrosome	55755	IMAGE:110741
Centrosome	55755	IMAGE:1656825
Centrosome	55755	IMAGE:241343
Centrosome	55755	IMAGE:431571

Protein complex	Gene ID	Clone ID
Centrosome	55755	IMAGE:447602
Centrosome	55755	IMAGE:450722
Centrosome	55755	IMAGE:487381
Centrosome	5576	IMAGE:586762
Centrosome	5576	IMAGE:611319
Centrosome	5576	IMAGE:743739
Centrosome	5577	IMAGE:1632376
Centrosome	5577	IMAGE:301068
Centrosome	5577	IMAGE:450924
Centrosome	5577	IMAGE:753286
Centrosome	55835	IMAGE:1843330
Centrosome	55835	IMAGE:431477
Centrosome	55835	IMAGE:786546
Centrosome	7283	IMAGE:108377
Centrosome	7532	IMAGE:127931
Centrosome	7532	IMAGE:784129
Centrosome	7840	1276377
Centrosome	7840	IMAGE:198011
Centrosome	7846	IMAGE:1459964
Centrosome	7846	IMAGE:1470060
Centrosome	79959	IMAGE:1031017
Centrosome	79959	IMAGE:1561593
Centrosome	79959	IMAGE:824617
Centrosome	80254	IMAGE:1693976
Centrosome	80254	IMAGE:262940
Centrosome	80254	IMAGE:273648
Centrosome	80254	IMAGE:427897
Centrosome	80321	IMAGE:256680
Centrosome	84131	IMAGE:1507062
Centrosome	84131	IMAGE:37820
Centrosome	84131	IMAGE:435619
Centrosome	8481	IMAGE:246703
Centrosome	8481	IMAGE:51052
Centrosome	85378	IMAGE:1422490
Centrosome	8655	IMAGE:853938
Centrosome	95681	IMAGE:1035759
Centrosome	95681	IMAGE:1636205
Centrosome	95681	IMAGE:1638525
Centrosome	95681	IMAGE:1855539
Centrosome	95681	IMAGE:772408
Centrosome	9662	IMAGE:151984
Centrosome	9696	IMAGE:39857
Centrosome	9702	IMAGE:154254
Centrosome	9702	IMAGE:154586
Centrosome	9702	IMAGE:1879938
Centrosome	9702	IMAGE:37355
Centrosome	9702	IMAGE:489662
Centrosome	9702	IMAGE:665082
Centrosome	9738	IMAGE:745339

Protein complex	Gene ID	Clone ID
Centrosome	9793	IMAGE:898032
COX	1329	IMAGE:143145
COX	1329	IMAGE:2326019
COX	1337	IMAGE:840894
COX	1339	IMAGE:1468213
COX	1340	IMAGE:1472754
COX	1340	IMAGE:298965
COX	1340	IMAGE:512003
COX	1349	IMAGE:884511
COX	1350	IMAGE:111924
COX	1350	IMAGE:884480
COX	1350	IMAGE:981458
COX	1350	IMAGE:982301
COX	1350	IMAGE:982361
Dynactin	10121	IMAGE:453109
Dynactin	10540	IMAGE:1849203
Dynactin	10540	IMAGE:725335
Dynactin	10671	IMAGE:788745
Dynactin	1639	IMAGE:877613
Dynactin	51164	IMAGE:877636
Dynactin	829	IMAGE:1031582
Dynactin	829	IMAGE:1558543
Dynactin	829	IMAGE:1982729
Dynactin	829	IMAGE:1983980
Dynactin	829	IMAGE:357052
Dynactin	829	IMAGE:785793
Dynactin	830	IMAGE:549073
Dynactin	832	IMAGE:1861608
Dynactin	832	IMAGE:1861763
Dynactin	832	IMAGE:1862179
Dynactin	832	IMAGE:322961
Dynactin	832	IMAGE:769911
Dynactin	93661	IMAGE:1586056
Dynactin	93661	IMAGE:1641927
Exocyst	10640	IMAGE:144905
Exocyst	10640	IMAGE:773591
Exocyst	10640	IMAGE:824406
Exocyst	10640	IMAGE:826230
Exocyst	23233	IMAGE:1020519
Exocyst	23233	IMAGE:147176
Exocyst	23233	IMAGE:258063
Exocyst	23233	IMAGE:453111
Exocyst	23265	IMAGE:159166
Exocyst	23265	IMAGE:32107
Exocyst	23265	IMAGE:796489
Exocyst	54536	IMAGE:1561191
Exocyst	54536	IMAGE:1926936
Exocyst	54536	IMAGE:625764
Exocyst	54536	IMAGE:745472

Protein complex	Gene ID	Clone ID
Exocyst	55763	IMAGE:1565038
Exocyst	55763	IMAGE:52063
Exocyst	55763	IMAGE:594428
Exocyst	55770	IMAGE:1155183
Exocyst	55770	IMAGE:1690442
Exocyst	55770	IMAGE:1934881
Exocyst	55770	IMAGE:1950056
Exocyst	55770	IMAGE:230316
Exocyst	55770	IMAGE:249784
Exocyst	55770	IMAGE:813195
Exocyst	60412	IMAGE:1848835
Exocyst	60412	IMAGE:1854822
Exocyst	60412	IMAGE:199327
Exocyst	60412	IMAGE:251212
Exocyst	60412	IMAGE:841079
Exocyst	60412	IMAGE:916505
Exocyst	60412	IMAGE:997280
Exosome	23016	IMAGE:1570436
Exosome	23016	IMAGE:982653
Exosome	51013	IMAGE:1740185
Exosome	5394	IMAGE:814069
Exosome	5394	IMAGE:841179
Exosome	54512	IMAGE:1420873
Exosome	54512	IMAGE:1630936
Exosome	56915	IMAGE:1559703
FA	10174	IMAGE:1755455
FA	1397	IMAGE:1607152
FA	1397	IMAGE:811046
FA	140885	IMAGE:1561161
FA	140885	IMAGE:1561183
FA	140885	IMAGE:2017171
FA	140885	IMAGE:2019426
FA	140885	IMAGE:49245
FA	1445	IMAGE:526282
FA	2119	IMAGE:1519951
FA	2119	IMAGE:796542
FA	2274	IMAGE:1031876
FA	2274	IMAGE:1606557
FA	2274	IMAGE:1711525
FA	23022	IMAGE:1640653
FA	23022	IMAGE:379397
FA	23022	IMAGE:66979
FA	23022	IMAGE:826174
FA	2316	IMAGE:898281
FA	2317	IMAGE:1708088
FA	2317	IMAGE:243652
FA	2317	IMAGE:840818
FA	2318	IMAGE:2321104
FA	2318	IMAGE:346897

Protein complex	Gene ID	Clone ID
FA	23683	IMAGE:162262
FA	23683	IMAGE:1755198
FA	23683	IMAGE:261541
FA	23683	IMAGE:752701
FA	23683	IMAGE:796932
FA	25	IMAGE:123858
FA	25	IMAGE:1671384
FA	25	IMAGE:1675071
FA	25	IMAGE:219976
FA	25	IMAGE:489968
FA	25	IMAGE:897642
FA	27111	IMAGE:1555478
FA	29780	IMAGE:2326042
FA	29780	IMAGE:75676
FA	3636	IMAGE:703964
FA	3987	IMAGE:1461477
FA	3987	IMAGE:365004
FA	3987	IMAGE:825416
FA	4478	IMAGE:131362
FA	4478	IMAGE:1896155
FA	4478	IMAGE:460569
FA	4478	IMAGE:81332
FA	4478	IMAGE:814265
FA	5058	IMAGE:53110
FA	5058	IMAGE:595200
FA	5062	IMAGE:134439
FA	5062	IMAGE:173561
FA	5063	IMAGE:132217
FA	5063	IMAGE:1690003
FA	5063	IMAGE:1691546
FA	5063	IMAGE:41316
FA	5063	IMAGE:51798
FA	5329	IMAGE:590154
FA	5329	IMAGE:810017
FA	5358	IMAGE:1568391
FA	5358	IMAGE:965085
FA	55742	IMAGE:1756621
FA	55742	IMAGE:486561
FA	5578	IMAGE:297136
FA	5578	IMAGE:469954
FA	5578	IMAGE:684338
FA	5578	IMAGE:768246
FA	5583	IMAGE:380245
FA	5583	IMAGE:502910
FA	5590	IMAGE:131239
FA	5590	IMAGE:167280
FA	5590	IMAGE:1843262
FA	5590	IMAGE:814266
FA	5747	IMAGE:1683906

Protein complex	Gene ID	Clone ID
FA	5747	IMAGE:1714018
FA	5747	IMAGE:1736335
FA	5747	IMAGE:213527
FA	5747	IMAGE:460882
FA	5747	IMAGE:724892
FA	5747	IMAGE:73222
FA	5747	IMAGE:855864
FA	5781	IMAGE:1562031
FA	5781	IMAGE:34773
FA	5792	IMAGE:1678361
FA	5792	IMAGE:450941
FA	5792	IMAGE:897788
FA	5829	IMAGE:770080
FA	6386	IMAGE:194906
FA	6386	IMAGE:813533
FA	64098	IMAGE:72498
FA	64098	IMAGE:824741
FA	6714	IMAGE:2107602
FA	7094	IMAGE:430894
FA	7094	IMAGE:435642
FA	7145	IMAGE:450523
FA	7408	IMAGE:753418
FA	7414	IMAGE:841203
FA	81	IMAGE:122138
FA	81	IMAGE:1839363
FA	81	IMAGE:213535
FA	824	IMAGE:549728
FA	83660	IMAGE:1565179
FA	83660	IMAGE:1735015
FA	83660	IMAGE:2018945
FA	83660	IMAGE:416409
FA	83660	IMAGE:490970
FA	84309	IMAGE:824879
FA	857	IMAGE:290525
FA	857	IMAGE:377461
FA	857	IMAGE:841664
FA	858	IMAGE:110467
FA	858	IMAGE:1946448
FA	858	IMAGE:840984
FA	859	IMAGE:773276
FA	87	IMAGE:854079
FA	9459	IMAGE:687990
GTC	10466	IMAGE:767193
GTC	22796	IMAGE:825296
GTC	25839	IMAGE:1010089
GTC	25839	IMAGE:1010093
GTC	57511	IMAGE:144861
GTC	57511	IMAGE:1926715
GTC	57511	IMAGE:268780

Protein complex	Gene ID	Clone ID
GTC	57511	IMAGE:343352
GTC	91949	IMAGE:204536
GTC	91949	IMAGE:40010
GTC	91949	IMAGE:526032
GTC	9382	IMAGE:125608
GTC	9382	IMAGE:126277
GTC	9382	IMAGE:1689980
GTC	9382	IMAGE:194023
GTC	9382	IMAGE:289637
GTC	9382	IMAGE:356711
GTC	9382	IMAGE:397604
LRS	11224	IMAGE:1737852
LRS	11224	IMAGE:745226
LRS	11224	IMAGE:877835
LRS	23521	IMAGE:1962510
LRS	4736	IMAGE:124824
LRS	6122	IMAGE:365945
LRS	6125	IMAGE:897596
LRS	6128	IMAGE:884546
LRS	6130	IMAGE:51981
LRS	6135	IMAGE:1220403
LRS	6135	IMAGE:869450
LRS	6138	IMAGE:1686496
LRS	6138	IMAGE:837904
LRS	6141	IMAGE:878545
LRS	6142	IMAGE:1582245
LRS	6158	IMAGE:841044
LRS	6164	IMAGE:138007
LRS	6164	IMAGE:178137
LRS	6164	IMAGE:462234
LRS	6165	IMAGE:1416101
LRS	6165	IMAGE:253314
LRS	6167	IMAGE:1564426
LRS	6167	IMAGE:344975
LRS	7311	IMAGE:1908834
LRS	9045	IMAGE:1861366
LRS	9045	IMAGE:1862035
LRS	9349	IMAGE:796885
MLRS	10573	IMAGE:1592276
MLRS	11222	IMAGE:44255
MLRS	114294	IMAGE:1875258
MLRS	114294	IMAGE:665650
MLRS	116541	IMAGE:1901924
MLRS	128308	IMAGE:292232
MLRS	128308	IMAGE:772898
MLRS	219927	IMAGE:809517
MLRS	26589	IMAGE:784214
MLRS	28977	IMAGE:1456721
MLRS	28977	IMAGE:203114

Protein complex	Gene ID	Clone ID
MLRS	28977	IMAGE:277163
MLRS	28998	IMAGE:491524
MLRS	29074	IMAGE:796281
MLRS	29093	IMAGE:590398
MLRS	51069	IMAGE:309494
MLRS	51073	IMAGE:1845176
MLRS	51073	IMAGE:824568
MLRS	51253	IMAGE:74208
MLRS	51253	IMAGE:843263
MLRS	51263	IMAGE:1584573
MLRS	51263	IMAGE:85318
MLRS	51264	IMAGE:1736730
MLRS	51264	IMAGE:417801
MLRS	51318	IMAGE:417208
MLRS	51318	IMAGE:490147
MLRS	51642	IMAGE:208656
MLRS	51642	IMAGE:232670
MLRS	51642	IMAGE:767402
MLRS	54148	IMAGE:127881
MLRS	54148	IMAGE:1536455
MLRS	54534	IMAGE:435415
MLRS	54948	IMAGE:126239
MLRS	54948	IMAGE:824911
MLRS	6150	IMAGE:788334
MLRS	6182	IMAGE:1636069
MLRS	63875	IMAGE:1745396
MLRS	64928	IMAGE:1570318
MLRS	64975	IMAGE:1031727
MLRS	64976	IMAGE:878525
MLRS	64979	IMAGE:811867
MLRS	64983	IMAGE:291633
MLRS	64983	IMAGE:950422
MLRS	65003	IMAGE:825229
MLRS	65008	IMAGE:321359
MLRS	65008	IMAGE:824122
MLRS	79590	IMAGE:416436
MLRS	84311	IMAGE:1917924
MLRS	84311	IMAGE:282094
MLRS	84311	IMAGE:590298
MLRS	84545	IMAGE:298648
MLRS	84545	IMAGE:376214
MLRS	9553	IMAGE:325520
MLRS	9553	IMAGE:897448
MLRS	9801	IMAGE:563701
MLRS	9801	IMAGE:827144
MSRS	10240	IMAGE:1492963
MSRS	10240	IMAGE:39313
MSRS	10884	IMAGE:1533748
MSRS	10884	IMAGE:1573946

Protein complex	Gene ID	Clone ID
MSRS	10884	IMAGE:1667337
MSRS	10884	IMAGE:270558
MSRS	10884	IMAGE:292469
MSRS	23107	IMAGE:843133
MSRS	28957	IMAGE:213585
MSRS	28957	IMAGE:768961
MSRS	28957	IMAGE:773483
MSRS	28973	IMAGE:592491
MSRS	28973	IMAGE:812988
MSRS	51021	IMAGE:1500480
MSRS	51021	IMAGE:685922
MSRS	51023	IMAGE:294304
MSRS	51081	IMAGE:505339
MSRS	51116	IMAGE:810979
MSRS	51373	IMAGE:1913855
MSRS	51649	IMAGE:824723
MSRS	51650	IMAGE:127396
MSRS	51650	IMAGE:194050
MSRS	51650	IMAGE:896914
MSRS	54460	IMAGE:26307
MSRS	54460	IMAGE:451733
MSRS	54460	IMAGE:771173
MSRS	55173	IMAGE:810753
MSRS	56945	IMAGE:2028599
MSRS	60488	IMAGE:246041
MSRS	6183	IMAGE:131653
MSRS	63931	IMAGE:796255
MSRS	64432	IMAGE:1007835
MSRS	64432	IMAGE:1509461
MSRS	64432	IMAGE:272632
MSRS	64432	IMAGE:279974
MSRS	64432	IMAGE:299468
MSRS	64949	IMAGE:810027
MSRS	64951	IMAGE:811770
MSRS	64960	IMAGE:590227
MSRS	64963	IMAGE:1646738
MSRS	64963	IMAGE:1909574
MSRS	64965	IMAGE:279691
MSRS	64965	IMAGE:431823
MSRS	64968	IMAGE:1849771
MSRS	64968	IMAGE:2013496
MSRS	64968	IMAGE:345743
MSRS	64968	IMAGE:489560
MSRS	64968	IMAGE:785819
MSRS	64968	IMAGE:839882
MSRS	64969	IMAGE:1882703
MSRS	64969	IMAGE:526615
MSRS	64969	IMAGE:812169
MSRS	65993	IMAGE:1938763

Protein complex	Gene ID	Clone ID
MSRS	65993	IMAGE:878333
MSRS	7818	IMAGE:1753346
MSRS	7818	IMAGE:877627
MSRS	78988	IMAGE:1604174
MSRS	78988	IMAGE:1640535
MSRS	78988	IMAGE:897042
MSRS	92259	IMAGE:1034724
MSRS	92259	IMAGE:1128327
MSRS	92259	IMAGE:1492910
MSRS	92259	IMAGE:1700088
MSRS	92259	IMAGE:1740175
MSRS	92259	IMAGE:1894441
MSRS	92259	IMAGE:845713
Nucleopore	10762	IMAGE:1948154
Nucleopore	10762	IMAGE:730361
Nucleopore	22981	IMAGE:1652663
Nucleopore	23165	IMAGE:950369
Nucleopore	23225	IMAGE:122782
Nucleopore	23225	IMAGE:825647
Nucleopore	23279	IMAGE:33299
Nucleopore	23279	IMAGE:565758
Nucleopore	23511	IMAGE:1587852
Nucleopore	23511	IMAGE:1880117
Nucleopore	3187	IMAGE:1055775
Nucleopore	3187	IMAGE:129185
Nucleopore	3187	IMAGE:195127
Nucleopore	3187	IMAGE:358457
Nucleopore	3187	IMAGE:447583
Nucleopore	3187	IMAGE:950372
Nucleopore	3188	IMAGE:866874
Nucleopore	3837	IMAGE:564537
Nucleopore	3837	IMAGE:768237
Nucleopore	3838	IMAGE:824962
Nucleopore	3838	IMAGE:882510
Nucleopore	4000	IMAGE:897544
Nucleopore	4670	IMAGE:123761
Nucleopore	4670	IMAGE:1842391
Nucleopore	4670	IMAGE:1951939
Nucleopore	4670	IMAGE:825411
Nucleopore	4927	IMAGE:1560927
Nucleopore	4927	IMAGE:843070
Nucleopore	4928	IMAGE:825787
Nucleopore	53371	IMAGE:287122
Nucleopore	53371	IMAGE:399562
Nucleopore	55746	IMAGE:1877435
Nucleopore	55746	IMAGE:233249
Nucleopore	55746	IMAGE:34345
Nucleopore	55746	IMAGE:627086
Nucleopore	57122	IMAGE:257955

Protein complex	Gene ID	Clone ID
Nucleopore	57122	IMAGE:827159
Nucleopore	5901	IMAGE:811956
Nucleopore	5905	IMAGE:122019
Nucleopore	5905	IMAGE:1619759
Nucleopore	5905	IMAGE:260931
Nucleopore	5905	IMAGE:811150
Nucleopore	5906	IMAGE:704905
Nucleopore	59343	IMAGE:462099
Nucleopore	59343	IMAGE:627112
Nucleopore	59343	IMAGE:629885
Nucleopore	6396	IMAGE:1701414
Nucleopore	6396	IMAGE:1846749
Nucleopore	6396	IMAGE:461749
Nucleopore	6396	IMAGE:897636
Nucleopore	7329	IMAGE:841292
Nucleopore	7431	IMAGE:590323
Nucleopore	7431	IMAGE:840511
Nucleopore	8021	IMAGE:194353
Nucleopore	8021	IMAGE:2104994
Nucleopore	8021	IMAGE:743188
Nucleopore	8021	IMAGE:823992
Nucleopore	84823	IMAGE:815501
Nucleopore	9631	IMAGE:130371
Nucleopore	9688	IMAGE:51918
Nucleopore	9972	IMAGE:1639278
Nucleopore	9972	IMAGE:2118291
Nucleopore	9972	IMAGE:27548
Nucleopore	9972	IMAGE:825861
Nucleosome	3005	IMAGE:205445
Nucleosome	3005	IMAGE:343744
Nucleosome	3005	IMAGE:80100
Nucleosome	3006	IMAGE:66317
Nucleosome	3012	IMAGE:757500
Nucleosome	3014	IMAGE:256664
Nucleosome	3014	IMAGE:824130
Nucleosome	3015	IMAGE:2315147
Nucleosome	3017	IMAGE:1500000
Nucleosome	3017	IMAGE:1500162
Nucleosome	3017	IMAGE:1604793
Nucleosome	3017	IMAGE:1658225
Nucleosome	3017	IMAGE:1688748
Nucleosome	3017	IMAGE:243784
Nucleosome	3017	IMAGE:31008
Nucleosome	3020	IMAGE:884272
Nucleosome	3021	IMAGE:950574
Nucleosome	3024	IMAGE:1872543
Nucleosome	55766	IMAGE:289734
Nucleosome	8334	IMAGE:124128
Nucleosome	8334	IMAGE:241274

Protein complex	Gene ID	Clone ID
Nucleosome	8334	IMAGE:283919
Nucleosome	8334	IMAGE:789091
Nucleosome	8336	IMAGE:1687138
Nucleosome	8337	IMAGE:130004
Nucleosome	8337	IMAGE:488964
Nucleosome	8337	IMAGE:754628
Nucleosome	8340	IMAGE:1579090
Nucleosome	8340	IMAGE:1751775
Nucleosome	8340	IMAGE:2056049
Nucleosome	8349	IMAGE:430235
Nucleosome	8349	IMAGE:461592
Nucleosome	8349	IMAGE:813149
Nucleosome	8351	IMAGE:2114004
Nucleosome	8363	IMAGE:2308333
Nucleosome	8365	IMAGE:1687361
Nucleosome	8365	IMAGE:447715
Nucleosome	8366	IMAGE:1842170
Nucleosome	8970	IMAGE:1561712
Nucleosome	8970	IMAGE:1675553
Nucleosome	8971	IMAGE:347560
Nucleosome	92815	IMAGE:1911334
Nucleosome	92815	IMAGE:838774
Nucleosome	94239	IMAGE:112161
Nucleosome	94239	IMAGE:1917941
Nucleosome	94239	IMAGE:248734
Nucleosome	94239	IMAGE:249949
Nucleosome	94239	IMAGE:322460
Nucleosome	94239	IMAGE:451649
Nucleosome	9555	IMAGE:1657118
Nucleosome	9555	IMAGE:240990
Nucleosome	9555	IMAGE:843075
ORC	23595	IMAGE:260336
ORC	4998	IMAGE:121154
ORC	4998	IMAGE:121341
ORC	4998	IMAGE:194236
ORC	4999	IMAGE:295630
ORC	5000	IMAGE:1535410
ORC	5000	IMAGE:878707
ORC	5001	IMAGE:1007141
ORC	5001	IMAGE:1585549
PD	1737	IMAGE:124252
PD	1737	IMAGE:271006
PD	1738	IMAGE:1542716
PD	1738	IMAGE:1850936
PD	1738	IMAGE:417957
PD	1738	IMAGE:813648
PD	5162	IMAGE:1639456
PD	5162	IMAGE:826077
PD	5163	IMAGE:1645668

Protein complex	Gene ID	Clone ID
PD	5163	IMAGE:1942432
PD	5163	IMAGE:361190
PD	5163	IMAGE:436761
PD	5164	IMAGE:49860
PD	5164	IMAGE:509589
PD	5165	IMAGE:278242
PD	8050	IMAGE:23576
PD	8050	IMAGE:25156
PD	8050	IMAGE:279665
Proteasome	5682	1292733
Proteasome	5682	IMAGE:134544
Proteasome	5682	IMAGE:298775
Proteasome	5682	IMAGE:451121
Proteasome	5683	IMAGE:155434
Proteasome	5683	IMAGE:366104
Proteasome	5684	IMAGE:1032540
Proteasome	5684	IMAGE:814246
Proteasome	5685	IMAGE:531862
Proteasome	5685	IMAGE:645184
Proteasome	5686	IMAGE:897952
Proteasome	5687	IMAGE:1323775
Proteasome	5687	IMAGE:1505686
Proteasome	5687	IMAGE:509495
Proteasome	5688	IMAGE:2054635
Proteasome	5688	IMAGE:435160
Proteasome	5689	IMAGE:82131
Proteasome	5690	IMAGE:122241
Proteasome	5690	IMAGE:593838
Proteasome	5690	IMAGE:731223
Proteasome	5691	IMAGE:951233
Proteasome	5693	IMAGE:1460110
Proteasome	5693	IMAGE:293675
Proteasome	5693	IMAGE:294485
Proteasome	5694	IMAGE:529861
Proteasome	5695	IMAGE:843352
Proteasome	5701	IMAGE:396272
Proteasome	5701	IMAGE:684655
Proteasome	5702	IMAGE:1602493
Proteasome	5702	IMAGE:712916
Proteasome	5704	IMAGE:756157
Proteasome	5704	IMAGE:810558
Proteasome	5706	IMAGE:1932026
Proteasome	5706	IMAGE:198614
Proteasome	5706	IMAGE:771900
Proteasome	5709	IMAGE:66377
Proteasome	5709	IMAGE:815861
Proteasome	5717	IMAGE:1553306
Proteasome	5717	IMAGE:179443
Proteasome	5717	IMAGE:450943

Protein complex	Gene ID	Clone ID
Proteasome	5717	IMAGE:566474
Proteasome	5718	IMAGE:823598
Proteasome	5719	IMAGE:1690879
Proteasome	5719	IMAGE:1696289
Proteasome	5719	IMAGE:2306804
RFC	5981	IMAGE:214537
RFC	5981	IMAGE:289787
RFC	5981	IMAGE:50648
RFC	5982	IMAGE:860000
RFC	5983	IMAGE:256260
RFC	5983	IMAGE:277112
RFC	5983	IMAGE:433683
RFC	5983	IMAGE:757158
RFC	5984	IMAGE:203275
RFC	5984	IMAGE:309288
RNA Pol II	5430	IMAGE:1033509
RNA Pol II	5430	IMAGE:1895032
RNA Pol II	5430	IMAGE:430236
RNA Pol II	5430	IMAGE:740130
RNA Pol II	5431	IMAGE:121580
RNA Pol II	5431	IMAGE:1876492
RNA Pol II	5431	IMAGE:293063
RNA Pol II	5431	IMAGE:295551
RNA Pol II	5431	IMAGE:462603
RNA Pol II	5432	IMAGE:770391
RNA Pol II	5433	IMAGE:49548
RNA Pol II	5434	IMAGE:469369
RNA Pol II	5435	IMAGE:767817
RNA Pol II	5436	IMAGE:109269
RNA Pol II	5437	IMAGE:2168667
RNA Pol II	5438	IMAGE:280801
RNA Pol II	5438	IMAGE:283615
RNA Pol II	5440	IMAGE:813410
RNA Pol III	10621	IMAGE:263059
RNA Pol III	10623	IMAGE:711959
RNA Pol III	10623	IMAGE:839092
RNA Pol III	11128	IMAGE:1734528
RNA Pol III	51728	IMAGE:511632
RNA Pol III	55703	IMAGE:199644
RNA Pol III	55703	IMAGE:488683
RNA Pol III	661	IMAGE:1543938
RNA Pol III	661	IMAGE:1557660
SCF	6500	IMAGE:262044
SCF	6500	IMAGE:291464
SCF	8454	IMAGE:1751841
SCF	8454	IMAGE:1842546
SCF	8454	IMAGE:450883
SCF	8454	IMAGE:841093
SCF	9978	IMAGE:200144

Protein complex	Gene ID	Clone ID
SCF	9978	IMAGE:433666
SNARE	10282	IMAGE:1010015
SNARE	10282	IMAGE:1010420
SNARE	10282	IMAGE:203351
SNARE	10490	IMAGE:1915012
SNARE	10652	IMAGE:810762
SNARE	26984	IMAGE:324745
SNARE	6811	IMAGE:2047655
SNARE	6811	IMAGE:297099
SNARE	6811	IMAGE:787857
SNARE	9527	IMAGE:324694
SNARE	9570	IMAGE:130773
SNARE	9570	IMAGE:145388
SNARE	9570	IMAGE:1750504
SNARE	9570	IMAGE:1943138
SNARE	9570	IMAGE:239708
SNARE	9570	IMAGE:66391
SNARE	9570	IMAGE:841697
SRP	6727	IMAGE:1602619
SRP	6728	IMAGE:1894034
SRP	6728	IMAGE:754998
SRP	6729	IMAGE:1846354
SRP	6729	IMAGE:196638
SRP	6729	IMAGE:950430
SRP	6730	IMAGE:1952876
SRP	6730	IMAGE:626358
SRP	6730	IMAGE:814791
SRP	6731	IMAGE:1755103
SRP	6731	IMAGE:321510
SRP	6731	IMAGE:358713
SRP	6731	IMAGE:811842
SRP	6731	IMAGE:814702
SRS	2197	IMAGE:1985178
SRS	2197	IMAGE:2249522
SRS	51065	IMAGE:590338
SRS	6188	IMAGE:487445
SRS	6193	IMAGE:50918
SRS	6193	IMAGE:809578
SRS	6194	IMAGE:303048
SRS	6201	IMAGE:2321118
SRS	6202	IMAGE:62257
SRS	6202	IMAGE:971367
SRS	6203	IMAGE:1861627
SRS	6204	1293191
SRS	6204	IMAGE:111136
SRS	6204	IMAGE:139641
SRS	6204	IMAGE:1422366
SRS	6204	IMAGE:1468533
SRS	6204	IMAGE:1578195

Protein complex	Gene ID	Clone ID
SRS	6204	IMAGE:1580126
SRS	6204	IMAGE:1629289
SRS	6204	IMAGE:1636549
SRS	6204	IMAGE:1639217
SRS	6204	IMAGE:1683873
SRS	6204	IMAGE:1733293
SRS	6204	IMAGE:1841990
SRS	6204	IMAGE:1848093
SRS	6204	IMAGE:1901173
SRS	6204	IMAGE:1915149
SRS	6204	IMAGE:1915897
SRS	6204	IMAGE:1917897
SRS	6204	IMAGE:2252417
SRS	6204	IMAGE:251195
SRS	6204	IMAGE:280967
SRS	6204	IMAGE:46977
SRS	6204	IMAGE:487035
SRS	6204	IMAGE:509949
SRS	6204	IMAGE:565905
SRS	6204	IMAGE:780997
SRS	6206	IMAGE:2271240
SRS	6207	IMAGE:857243
SRS	6208	IMAGE:1697368
SRS	6208	IMAGE:741522
SRS	6208	IMAGE:756820
SRS	6217	IMAGE:853151
SRS	6223	IMAGE:192242
SRS	6223	IMAGE:1926032
SRS	6223	IMAGE:1983719
SRS	6224	IMAGE:2007305
SRS	6224	IMAGE:80265
SRS	6227	IMAGE:2272597
SRS	6228	IMAGE:291974
SRS	6228	IMAGE:49392
SRS	6228	IMAGE:511882
SRS	6228	IMAGE:511991
SRS	6228	IMAGE:868308
SRS	6229	IMAGE:1055201
SRS	6229	IMAGE:1637296
SRS	6229	IMAGE:461071
SRS	6230	IMAGE:454905
SRS	6230	IMAGE:896930
SRS	6235	IMAGE:1950606
SWI-SNF	55193	IMAGE:1556449
SWI-SNF	55193	IMAGE:214744
SWI-SNF	55193	IMAGE:239615
SWI-SNF	55193	IMAGE:296883
SWI-SNF	55193	IMAGE:427811
SWI-SNF	55193	IMAGE:811981

Protein complex	Gene ID	Clone ID
SWI-SNF	55193	IMAGE:813644
SWI-SNF	6595	IMAGE:142540
SWI-SNF	6595	IMAGE:1640707
SWI-SNF	6595	IMAGE:1848857
SWI-SNF	6595	IMAGE:814636
SWI-SNF	6597	IMAGE:1011163
SWI-SNF	6597	IMAGE:1011375
SWI-SNF	6597	IMAGE:897890
SWI-SNF	6598	IMAGE:121605
SWI-SNF	6598	IMAGE:1467420
SWI-SNF	6598	IMAGE:781018
SWI-SNF	6598	IMAGE:80271
SWI-SNF	6599	IMAGE:490813
SWI-SNF	6601	IMAGE:160838
SWI-SNF	6603	IMAGE:741067
SWI-SNF	6604	IMAGE:360047
SWI-SNF	8289	IMAGE:1881595
SWI-SNF	8289	IMAGE:461442
SWI-SNF	8289	IMAGE:810843
SWI-SNF	86	IMAGE:753400
TAFIID	10629	IMAGE:1670689
TAFIID	10629	IMAGE:268803
TAFIID	27097	IMAGE:377521
TAFIID	54457	IMAGE:743116
TAFIID	6872	IMAGE:222460
TAFIID	6872	IMAGE:299093
TAFIID	6872	IMAGE:454440
TAFIID	6873	IMAGE:136730
TAFIID	6873	IMAGE:462691
TAFIID	6877	IMAGE:1587429
TAFIID	6878	IMAGE:33438
TAFIID	6879	IMAGE:242700
TAFIID	6879	IMAGE:365930
TAFIID	6879	IMAGE:795850
TAFIID	6880	IMAGE:564197
TAFIID	6881	IMAGE:723972
TAFIID	6882	IMAGE:306444
TAFIID	6883	IMAGE:509588
TAFIID	6908	IMAGE:280735
TRAPP	122553	IMAGE:745426
TRAPP	126003	IMAGE:668007
TRAPP	126003	IMAGE:878403
TRAPP	27095	IMAGE:460580
TRAPP	27095	IMAGE:780977
TRAPP	51399	IMAGE:897153
TRAPP	58485	IMAGE:135710
VHL	6923	IMAGE:884692
VHL	7428	IMAGE:234856
VHL	8453	IMAGE:788247

VHL	9978	IMAGE:200144
VHL	9978	IMAGE:433666

C

Protein complex	Gene ID	Clone ID
AP2	1173	IMAGE:1846645
AP2	1175	IMAGE:739109
AP2	161	IMAGE:1469115
AP2	161	IMAGE:1631984
AP2	163	IMAGE:245853
AP2	163	IMAGE:416340
APC	10393	IMAGE:2114111
APC	10393	IMAGE:270932
APC	10393	IMAGE:966335
APC	29945	IMAGE:754377
APC	51433	IMAGE:823954
APC	51434	IMAGE:131110
APC	51434	IMAGE:66919
APC	51434	IMAGE:786096
APC	64682	IMAGE:1534603
APC	64682	IMAGE:45771
APC	64682	IMAGE:511267
APC	64682	IMAGE:731433
APC	8697	IMAGE:1871423
APC	8697	IMAGE:240969
APC	8697	IMAGE:26462
APC	8881	IMAGE:1506477
APC	8881	IMAGE:197779
APC	8881	IMAGE:755385
APC	996	IMAGE:109708
APC	996	IMAGE:281585
APC	996	IMAGE:504396
APC	996	IMAGE:75639
APC	996	IMAGE:842946
APC	996	IMAGE:869458
ARC	1452	IMAGE:433490
ARC	1452	IMAGE:745402
ARC	1499	IMAGE:1900880
ARC	1499	IMAGE:774754
ARC	2932	IMAGE:197128
ARC	324	IMAGE:1742727
ARC	324	IMAGE:206838
ARC	324	IMAGE:269332
ARC	8312	IMAGE:1461733
ARC	8312	IMAGE:1506605
ARC	8312	IMAGE:1754823
Arp2-3	10092	IMAGE:340558
Arp2-3	10094	IMAGE:232826
Arp2-3	10096	1293112
Arp2-3	10096	IMAGE:194587
Arp2-3	10096	IMAGE:271568

Protein complex	Gene ID	Clone ID
Arp2-3	10096	IMAGE:593251
Arp2-3	10096	IMAGE:813543
Arp2-3	10097	IMAGE:1584391
Arp2-3	10109	IMAGE:162208
ATP_F0	10476	IMAGE:825386
ATP_F0	267020	IMAGE:461264
ATP_F0	27109	IMAGE:198190
ATP_F0	27109	IMAGE:254533
ATP_F0	27109	IMAGE:321163
ATP_F0	27109	IMAGE:379343
ATP_F0	515	IMAGE:813712
ATP_F0	516	IMAGE:487373
ATP_F0	517	IMAGE:809876
ATP_F0	518	IMAGE:193106
ATP_F0	518	IMAGE:611150
ATP_F0	521	IMAGE:782439
ATP_F0	522	1048899
ATP_F0	522	IMAGE:139199
ATP_F0	522	IMAGE:564558
ATP_F0	522	IMAGE:825312
ATP_F0	9551	IMAGE:1574131
ATP_F1	509	IMAGE:845519
ATP_F1	513	IMAGE:856650
ATP_F1	514	IMAGE:434968
Centrosome	10121	IMAGE:342342
Centrosome	10121	IMAGE:453109
Centrosome	10142	IMAGE:197672
Centrosome	10142	IMAGE:2306682
Centrosome	10142	IMAGE:40263
Centrosome	10426	IMAGE:1029186
Centrosome	10426	IMAGE:244955
Centrosome	10426	IMAGE:950746
Centrosome	10426	IMAGE:964784
Centrosome	10540	IMAGE:1849203
Centrosome	10540	IMAGE:725335
Centrosome	1070	IMAGE:487425
Centrosome	10733	IMAGE:2106955
Centrosome	10806	IMAGE:1691128
Centrosome	10806	IMAGE:248654
Centrosome	10806	IMAGE:417043
Centrosome	10806	IMAGE:785880
Centrosome	10844	IMAGE:453348
Centrosome	11064	IMAGE:210610
Centrosome	11064	IMAGE:430192
Centrosome	11190	IMAGE:165921
Centrosome	11190	IMAGE:1674822
Centrosome	1454	IMAGE:854138
Centrosome	1454	IMAGE:878111
Centrosome	1639	IMAGE:877613

Protein complex	Gene ID	Clone ID
Centrosome	1778	IMAGE:251618
Centrosome	1778	IMAGE:415406
Centrosome	1781	IMAGE:1646552
Centrosome	1781	IMAGE:206457
Centrosome	1781	IMAGE:243360
Centrosome	1781	IMAGE:843429
Centrosome	22897	IMAGE:122443
Centrosome	22919	IMAGE:428223
Centrosome	22995	IMAGE:1941598
Centrosome	22995	IMAGE:296334
Centrosome	23177	IMAGE:1679826
Centrosome	23177	IMAGE:1754869
Centrosome	23177	IMAGE:1878424
Centrosome	23177	IMAGE:1878917
Centrosome	23177	IMAGE:1893101
Centrosome	23177	IMAGE:1893445
Centrosome	23177	IMAGE:488160
Centrosome	23177	IMAGE:796747
Centrosome	23332	IMAGE:127102
Centrosome	23332	IMAGE:1901509
Centrosome	23332	IMAGE:41029
Centrosome	4751	IMAGE:198104
Centrosome	4751	IMAGE:415089
Centrosome	4957	IMAGE:504469
Centrosome	4957	IMAGE:743278
Centrosome	5048	IMAGE:212320
Centrosome	5048	IMAGE:245883
Centrosome	5048	IMAGE:878178
Centrosome	51199	IMAGE:129502
Centrosome	51199	IMAGE:1619448
Centrosome	51199	IMAGE:1935464
Centrosome	51199	IMAGE:197399
Centrosome	51199	IMAGE:271357
Centrosome	51199	IMAGE:430751
Centrosome	51199	IMAGE:683361
Centrosome	51199	IMAGE:815251
Centrosome	5347	IMAGE:744047
Centrosome	54820	IMAGE:810947
Centrosome	55125	IMAGE:434782
Centrosome	55142	IMAGE:1849940
Centrosome	55142	IMAGE:703569
Centrosome	5566	IMAGE:246705
Centrosome	5566	IMAGE:342543
Centrosome	55722	IMAGE:503824
Centrosome	55755	IMAGE:110741
Centrosome	55755	IMAGE:1656825
Centrosome	55755	IMAGE:241343
Centrosome	55755	IMAGE:431571
Centrosome	55755	IMAGE:447602

Protein complex	Gene ID	Clone ID
Centrosome	55755	IMAGE:450722
Centrosome	55755	IMAGE:487381
Centrosome	5576	IMAGE:586762
Centrosome	5576	IMAGE:611319
Centrosome	5576	IMAGE:743739
Centrosome	5577	IMAGE:1632376
Centrosome	5577	IMAGE:301068
Centrosome	5577	IMAGE:450924
Centrosome	5577	IMAGE:609663
Centrosome	5577	IMAGE:753286
Centrosome	55835	IMAGE:1843330
Centrosome	55835	IMAGE:431477
Centrosome	55835	IMAGE:786546
Centrosome	7283	IMAGE:108377
Centrosome	7532	IMAGE:127931
Centrosome	7532	IMAGE:784129
Centrosome	7840	1276377
Centrosome	7840	IMAGE:198011
Centrosome	7846	IMAGE:1459964
Centrosome	7846	IMAGE:1470060
Centrosome	79959	IMAGE:1031017
Centrosome	79959	IMAGE:1561593
Centrosome	79959	IMAGE:824617
Centrosome	80254	IMAGE:1693976
Centrosome	80254	IMAGE:262940
Centrosome	80254	IMAGE:273648
Centrosome	80254	IMAGE:427897
Centrosome	80321	IMAGE:256680
Centrosome	84131	IMAGE:1507062
Centrosome	84131	IMAGE:37820
Centrosome	84131	IMAGE:435619
Centrosome	8481	IMAGE:246703
Centrosome	8481	IMAGE:51052
Centrosome	85378	IMAGE:1422490
Centrosome	8636	IMAGE:1592048
Centrosome	8655	IMAGE:853938
Centrosome	95681	IMAGE:1035759
Centrosome	95681	IMAGE:1636205
Centrosome	95681	IMAGE:1638525
Centrosome	95681	IMAGE:1855539
Centrosome	9662	IMAGE:151984
Centrosome	9696	IMAGE:39857
Centrosome	9702	IMAGE:154254
Centrosome	9702	IMAGE:154586
Centrosome	9702	IMAGE:1879938
Centrosome	9702	IMAGE:37355
Centrosome	9702	IMAGE:489662
Centrosome	9702	IMAGE:665082
Centrosome	9738	IMAGE:745339

Protein complex	Gene ID	Clone ID
Centrosome	9793	IMAGE:898032
COX	1329	IMAGE:143145
COX	1329	IMAGE:2326019
COX	1337	IMAGE:840894
COX	1339	IMAGE:1468213
COX	1340	IMAGE:1472754
COX	1340	IMAGE:298965
COX	1340	IMAGE:512003
COX	1349	IMAGE:884511
COX	1350	IMAGE:111924
COX	1350	IMAGE:884480
COX	1350	IMAGE:981458
COX	170712	IMAGE:1736445
COX	9377	IMAGE:824068
Dynactin	10121	IMAGE:342342
Dynactin	10121	IMAGE:453109
Dynactin	10540	IMAGE:1849203
Dynactin	10540	IMAGE:725335
Dynactin	10671	IMAGE:788745
Dynactin	1639	IMAGE:877613
Dynactin	829	IMAGE:1031582
Dynactin	829	IMAGE:1558543
Dynactin	829	IMAGE:1982729
Dynactin	829	IMAGE:1983980
Dynactin	829	IMAGE:357052
Dynactin	829	IMAGE:785793
Dynactin	832	IMAGE:1861608
Dynactin	832	IMAGE:1861763
Dynactin	832	IMAGE:1862179
Dynactin	832	IMAGE:322961
Dynactin	832	IMAGE:769911
Dynactin	93661	IMAGE:1586056
Dynactin	93661	IMAGE:1641927
Exocyst	10640	IMAGE:144905
Exocyst	10640	IMAGE:773591
Exocyst	10640	IMAGE:824406
Exocyst	10640	IMAGE:826230
Exocyst	23233	IMAGE:1020519
Exocyst	23233	IMAGE:147176
Exocyst	23233	IMAGE:258063
Exocyst	23233	IMAGE:453111
Exocyst	23265	IMAGE:159166
Exocyst	23265	IMAGE:32107
Exocyst	23265	IMAGE:796489
Exocyst	54536	IMAGE:1561191
Exocyst	54536	IMAGE:1926936
Exocyst	54536	IMAGE:625764
Exocyst	54536	IMAGE:745472
Exocyst	55763	IMAGE:1565038

Protein complex	Gene ID	Clone ID
Exocyst	55763	IMAGE:52063
Exocyst	55763	IMAGE:594428
Exocyst	55770	IMAGE:1155183
Exocyst	55770	IMAGE:1690442
Exocyst	55770	IMAGE:1934881
Exocyst	55770	IMAGE:1950056
Exocyst	55770	IMAGE:230316
Exocyst	55770	IMAGE:249784
Exocyst	55770	IMAGE:813195
Exocyst	60412	IMAGE:1581477
Exocyst	60412	IMAGE:1848835
Exocyst	60412	IMAGE:1854822
Exocyst	60412	IMAGE:199327
Exocyst	60412	IMAGE:251212
Exocyst	60412	IMAGE:841079
Exocyst	60412	IMAGE:916505
Exocyst	60412	IMAGE:997280
Exosome	23016	IMAGE:1570436
Exosome	51013	IMAGE:1740185
Exosome	5394	IMAGE:814069
Exosome	5394	IMAGE:841179
Exosome	54512	IMAGE:1420873
Exosome	54512	IMAGE:1630936
Exosome	56915	IMAGE:1559703
FA	10174	IMAGE:1755455
FA	1397	IMAGE:1607152
FA	1397	IMAGE:811046
FA	140885	IMAGE:1561159
FA	140885	IMAGE:1561161
FA	140885	IMAGE:1561183
FA	140885	IMAGE:2019426
FA	140885	IMAGE:49245
FA	140885	IMAGE:731118
FA	1445	IMAGE:526282
FA	2119	IMAGE:1519951
FA	2119	IMAGE:796542
FA	2274	IMAGE:1031876
FA	2274	IMAGE:1606557
FA	2274	IMAGE:1711525
FA	23022	IMAGE:1640653
FA	23022	IMAGE:379397
FA	23022	IMAGE:66979
FA	23022	IMAGE:826174
FA	2316	IMAGE:898281
FA	2317	IMAGE:1708088
FA	2317	IMAGE:243652
FA	2317	IMAGE:840818
FA	2318	IMAGE:2321104
FA	2318	IMAGE:346897

FA	23683	IMAGE:162262

Protein complex	Gene ID	Clone ID
FA	23683	IMAGE:1755198
FA	23683	IMAGE:261541
FA	23683	IMAGE:752701
FA	23683	IMAGE:796932
FA	25	IMAGE:123858
FA	25	IMAGE:1671384
FA	25	IMAGE:1675071
FA	25	IMAGE:219976
FA	25	IMAGE:489968
FA	25	IMAGE:897642
FA	27111	IMAGE:1555478
FA	29780	IMAGE:2326042
FA	29780	IMAGE:75676
FA	3636	IMAGE:703964
FA	3987	IMAGE:1461477
FA	3987	IMAGE:365004
FA	3987	IMAGE:825416
FA	4478	IMAGE:131362
FA	4478	IMAGE:1896155
FA	4478	IMAGE:460569
FA	4478	IMAGE:81332
FA	4478	IMAGE:814265
FA	5058	IMAGE:53110
FA	5058	IMAGE:595200
FA	5062	IMAGE:134439
FA	5062	IMAGE:173561
FA	5063	IMAGE:132217
FA	5063	IMAGE:1690003
FA	5063	IMAGE:1691546
FA	5063	IMAGE:41316
FA	5063	IMAGE:51798
FA	5329	IMAGE:590154
FA	5329	IMAGE:810017
FA	5358	IMAGE:1568391
FA	5358	IMAGE:965085
FA	55742	IMAGE:1756621
FA	55742	IMAGE:486561
FA	5578	IMAGE:297136
FA	5578	IMAGE:469954
FA	5578	IMAGE:684338
FA	5578	IMAGE:768246
FA	5582	IMAGE:167032
FA	5583	IMAGE:380245
FA	5583	IMAGE:502910
FA	5590	IMAGE:120863
FA	5590	IMAGE:131239
FA	5590	IMAGE:167280
FA	5590	IMAGE:1843262

Protein complex	Gene ID	Clone ID
FA	5590	IMAGE:814266
FA	5747	IMAGE:1714018
FA	5747	IMAGE:1736335
FA	5747	IMAGE:213527
FA	5747	IMAGE:460882
FA	5747	IMAGE:724892
FA	5747	IMAGE:73222
FA	5747	IMAGE:855864
FA	5781	IMAGE:1562031
FA	5781	IMAGE:34773
FA	5781	IMAGE:814776
FA	5792	IMAGE:1678361
FA	5792	IMAGE:450941
FA	5792	IMAGE:897788
FA	5829	IMAGE:770080
FA	6385	IMAGE:504763
FA	6386	IMAGE:194906
FA	6386	IMAGE:813533
FA	64098	IMAGE:72498
FA	64098	IMAGE:824741
FA	6714	IMAGE:2107602
FA	7094	IMAGE:430894
FA	7094	IMAGE:435642
FA	7145	IMAGE:450523
FA	7408	IMAGE:753418
FA	7414	IMAGE:841203
FA	81	IMAGE:122138
FA	81	IMAGE:1839363
FA	81	IMAGE:213535
FA	824	IMAGE:549728
FA	83660	IMAGE:1565179
FA	83660	IMAGE:1735015
FA	83660	IMAGE:2018945
FA	83660	IMAGE:416409
FA	83660	IMAGE:490970
FA	84309	IMAGE:824879
FA	857	IMAGE:290525
FA	857	IMAGE:377461
FA	857	IMAGE:841664
FA	858	IMAGE:110467
FA	858	IMAGE:1946448
FA	858	IMAGE:840984
FA	87	IMAGE:854079
FA	9459	IMAGE:687990
GTC	10466	IMAGE:767193
GTC	22796	IMAGE:825296
GTC	25839	IMAGE:1010089
GTC	25839	IMAGE:1010093
GTC	57511	IMAGE:144861

Protein complex	Gene ID	Clone ID
GTC	57511	IMAGE:1926715
GTC	57511	IMAGE:268780
GTC	57511	IMAGE:343352
GTC	91949	IMAGE:204536
GTC	91949	IMAGE:40010
GTC	91949	IMAGE:526032
GTC	9382	IMAGE:125608
GTC	9382	IMAGE:126277
GTC	9382	IMAGE:1689980
GTC	9382	IMAGE:194023
GTC	9382	IMAGE:289637
GTC	9382	IMAGE:356711
GTC	9382	IMAGE:397604
LRS	11224	IMAGE:1737852
LRS	11224	IMAGE:745226
LRS	11224	IMAGE:877835
LRS	23521	IMAGE:1962510
LRS	4736	IMAGE:124824
LRS	6122	IMAGE:365945
LRS	6125	IMAGE:897596
LRS	6128	IMAGE:884546
LRS	6130	IMAGE:51981
LRS	6135	IMAGE:1220403
LRS	6135	IMAGE:869450
LRS	6138	IMAGE:1686496
LRS	6138	IMAGE:837904
LRS	6141	IMAGE:878545
LRS	6142	IMAGE:1582245
LRS	6158	IMAGE:841044
LRS	6164	IMAGE:138007
LRS	6164	IMAGE:178137
LRS	6164	IMAGE:462234
LRS	6165	IMAGE:1416101
LRS	6165	IMAGE:253314
LRS	6167	IMAGE:1564426
LRS	6167	IMAGE:344975
LRS	7311	IMAGE:1908834
LRS	9045	IMAGE:1861366
LRS	9045	IMAGE:1862035
LRS	9349	IMAGE:796885
MLRS	10573	IMAGE:1592276
MLRS	11222	IMAGE:44255
MLRS	114294	IMAGE:1875258
MLRS	114294	IMAGE:665650
MLRS	116541	IMAGE:1901924
MLRS	128308	IMAGE:292232
MLRS	128308	IMAGE:772898
MLRS	219927	IMAGE:809517
MLRS	26589	IMAGE:784214

Protein complex	Gene ID	Clone ID
MLRS	28977	IMAGE:1456721
MLRS	28977	IMAGE:203114
MLRS	28977	IMAGE:277163
MLRS	28998	IMAGE:491524
MLRS	29074	IMAGE:796281
MLRS	29093	IMAGE:590398
MLRS	51069	IMAGE:309494
MLRS	51073	IMAGE:1845176
MLRS	51073	IMAGE:824568
MLRS	51253	IMAGE:74208
MLRS	51253	IMAGE:843263
MLRS	51263	IMAGE:1584573
MLRS	51263	IMAGE:85318
MLRS	51264	IMAGE:1736730
MLRS	51264	IMAGE:417801
MLRS	51318	IMAGE:417208
MLRS	51318	IMAGE:490147
MLRS	51642	IMAGE:232670
MLRS	51642	IMAGE:767402
MLRS	54148	IMAGE:127881
MLRS	54148	IMAGE:1536455
MLRS	54148	IMAGE:324719
MLRS	54534	IMAGE:435415
MLRS	54948	IMAGE:126239
MLRS	54948	IMAGE:824911
MLRS	6150	IMAGE:788334
MLRS	6182	IMAGE:1636069
MLRS	63875	IMAGE:1745396
MLRS	64928	IMAGE:1570318
MLRS	64975	IMAGE:1031727
MLRS	64976	IMAGE:878525
MLRS	64978	IMAGE:809738
MLRS	64979	IMAGE:811867
MLRS	64983	IMAGE:291633
MLRS	64983	IMAGE:950422
MLRS	65003	IMAGE:825229
MLRS	65008	IMAGE:321359
MLRS	65008	IMAGE:824122
MLRS	79590	IMAGE:416436
MLRS	84311	IMAGE:1917924
MLRS	84311	IMAGE:282094
MLRS	84311	IMAGE:590298
MLRS	84545	IMAGE:298648
MLRS	84545	IMAGE:376214
MLRS	9553	IMAGE:325520
MLRS	9553	IMAGE:897448
MLRS	9801	IMAGE:563701
MLRS	9801	IMAGE:827144
MSRS	10240	IMAGE:1492963

Protein complex	Gene ID	Clone ID
MSRS	10240	IMAGE:39313
MSRS	10884	IMAGE:1533748
MSRS	10884	IMAGE:1573946
MSRS	10884	IMAGE:1667337
MSRS	10884	IMAGE:270558
MSRS	10884	IMAGE:292469
MSRS	23107	IMAGE:843133
MSRS	28957	IMAGE:213585
MSRS	28957	IMAGE:768961
MSRS	28957	IMAGE:773483
MSRS	28973	IMAGE:592491
MSRS	28973	IMAGE:812988
MSRS	51021	IMAGE:1500480
MSRS	51021	IMAGE:685922
MSRS	51021	IMAGE:755304
MSRS	51023	IMAGE:294304
MSRS	51081	IMAGE:505339
MSRS	51081	IMAGE:564625
MSRS	51116	IMAGE:810979
MSRS	51373	IMAGE:1913855
MSRS	51649	IMAGE:824723
MSRS	51650	IMAGE:127396
MSRS	51650	IMAGE:194050
MSRS	51650	IMAGE:896914
MSRS	54460	IMAGE:26307
MSRS	54460	IMAGE:451733
MSRS	54460	IMAGE:771173
MSRS	55173	IMAGE:810753
MSRS	56945	IMAGE:2028599
MSRS	60488	IMAGE:246041
MSRS	6183	IMAGE:131653
MSRS	63931	IMAGE:796255
MSRS	64432	IMAGE:1007835
MSRS	64432	IMAGE:1509461
MSRS	64432	IMAGE:272632
MSRS	64432	IMAGE:279974
MSRS	64432	IMAGE:299468
MSRS	64949	IMAGE:810027
MSRS	64951	IMAGE:811770
MSRS	64960	IMAGE:590227
MSRS	64963	IMAGE:1646738
MSRS	64963	IMAGE:1909574
MSRS	64963	IMAGE:271672
MSRS	64965	IMAGE:431823
MSRS	64968	IMAGE:1849771
MSRS	64968	IMAGE:2013496
MSRS	64968	IMAGE:345743
MSRS	64968	IMAGE:489560
MSRS	64968	IMAGE:744360

Protein complex	Gene ID	Clone ID
MSRS	64968	IMAGE:785819
MSRS	64968	IMAGE:839882
MSRS	64969	IMAGE:1882703
MSRS	64969	IMAGE:812169
MSRS	65993	IMAGE:1938763
MSRS	65993	IMAGE:878333
MSRS	7818	IMAGE:1753346
MSRS	7818	IMAGE:877627
MSRS	78988	IMAGE:1604174
MSRS	78988	IMAGE:1640535
MSRS	78988	IMAGE:897042
MSRS	92259	IMAGE:1034724
MSRS	92259	IMAGE:1128327
MSRS	92259	IMAGE:1492910
MSRS	92259	IMAGE:1700088
MSRS	92259	IMAGE:1740175
MSRS	92259	IMAGE:1894441
MSRS	92259	IMAGE:845713
Nucleopore	10762	IMAGE:1948154
Nucleopore	10762	IMAGE:730361
Nucleopore	22981	IMAGE:1652663
Nucleopore	23165	IMAGE:950369
Nucleopore	23225	IMAGE:122782
Nucleopore	23225	IMAGE:825647
Nucleopore	23279	IMAGE:33299
Nucleopore	23279	IMAGE:565758
Nucleopore	23511	IMAGE:1587852
Nucleopore	23511	IMAGE:1880117
Nucleopore	3187	IMAGE:1055775
Nucleopore	3187	IMAGE:129185
Nucleopore	3187	IMAGE:195127
Nucleopore	3187	IMAGE:358457
Nucleopore	3187	IMAGE:447583
Nucleopore	3187	IMAGE:950372
Nucleopore	3188	IMAGE:866874
Nucleopore	3837	IMAGE:564537
Nucleopore	3837	IMAGE:684634
Nucleopore	3837	IMAGE:768237
Nucleopore	3838	IMAGE:824962
Nucleopore	3838	IMAGE:882510
Nucleopore	4000	IMAGE:897544
Nucleopore	4670	IMAGE:123761
Nucleopore	4670	IMAGE:1842391
Nucleopore	4670	IMAGE:1951939
Nucleopore	4670	IMAGE:825411
Nucleopore	4927	IMAGE:1560927
Nucleopore	4927	IMAGE:843070
Nucleopore	4928	IMAGE:825787
Nucleopore	53371	IMAGE:287122

Protein complex	Gene ID	Clone ID
Nucleopore	53371	IMAGE:399562
Nucleopore	55746	IMAGE:1877435
Nucleopore	55746	IMAGE:233249
Nucleopore	55746	IMAGE:34345
Nucleopore	55746	IMAGE:627086
Nucleopore	57122	IMAGE:257955
Nucleopore	57122	IMAGE:827159
Nucleopore	5901	IMAGE:811956
Nucleopore	5905	IMAGE:122019
Nucleopore	5905	IMAGE:1619759
Nucleopore	5905	IMAGE:260931
Nucleopore	5905	IMAGE:811150
Nucleopore	5906	IMAGE:704905
Nucleopore	59343	IMAGE:462099
Nucleopore	59343	IMAGE:627112
Nucleopore	59343	IMAGE:629885
Nucleopore	6396	IMAGE:1701414
Nucleopore	6396	IMAGE:1846749
Nucleopore	6396	IMAGE:461749
Nucleopore	6396	IMAGE:897636
Nucleopore	7329	IMAGE:841292
Nucleopore	7431	IMAGE:590323
Nucleopore	7431	IMAGE:840511
Nucleopore	8021	IMAGE:194353
Nucleopore	8021	IMAGE:2104994
Nucleopore	8021	IMAGE:743188
Nucleopore	8021	IMAGE:823992
Nucleopore	8480	IMAGE:609530
Nucleopore	8480	IMAGE:825224
Nucleopore	84823	IMAGE:815501
Nucleopore	9631	IMAGE:130371
Nucleopore	9688	IMAGE:51918
Nucleopore	9688	IMAGE:769751
Nucleopore	9972	IMAGE:1639278
Nucleopore	9972	IMAGE:2118291
Nucleopore	9972	IMAGE:27548
Nucleopore	9972	IMAGE:825861
Nucleosome	3005	IMAGE:205445
Nucleosome	3005	IMAGE:343744
Nucleosome	3005	IMAGE:80100
Nucleosome	3006	IMAGE:66317
Nucleosome	3012	IMAGE:757500
Nucleosome	3014	IMAGE:256664
Nucleosome	3014	IMAGE:824130
Nucleosome	3015	IMAGE:2315147
Nucleosome	3017	IMAGE:1500000
Nucleosome	3017	IMAGE:1500162
Nucleosome	3017	IMAGE:1604793
Nucleosome	3017	IMAGE:1658225

Protein complex	Gene ID	Clone ID
Nucleosome	3017	IMAGE:1688748
Nucleosome	3017	IMAGE:243784
Nucleosome	3017	IMAGE:31008
Nucleosome	3020	IMAGE:884272
Nucleosome	3021	IMAGE:950574
Nucleosome	3024	IMAGE:1872543
Nucleosome	55766	IMAGE:289734
Nucleosome	8334	IMAGE:124128
Nucleosome	8334	IMAGE:241274
Nucleosome	8334	IMAGE:283919
Nucleosome	8334	IMAGE:789091
Nucleosome	8336	IMAGE:1687138
Nucleosome	8337	IMAGE:130004
Nucleosome	8337	IMAGE:488964
Nucleosome	8337	IMAGE:754628
Nucleosome	8340	IMAGE:1579090
Nucleosome	8340	IMAGE:1751775
Nucleosome	8340	IMAGE:2056049
Nucleosome	8349	IMAGE:430235
Nucleosome	8349	IMAGE:461592
Nucleosome	8349	IMAGE:813149
Nucleosome	8351	IMAGE:2114004
Nucleosome	8363	IMAGE:2308333
Nucleosome	8365	IMAGE:1687361
Nucleosome	8365	IMAGE:447715
Nucleosome	8366	IMAGE:1842170
Nucleosome	8970	IMAGE:1561712
Nucleosome	8970	IMAGE:1675553
Nucleosome	8971	IMAGE:347560
Nucleosome	92815	IMAGE:1911334
Nucleosome	92815	IMAGE:838774
Nucleosome	94239	IMAGE:112161
Nucleosome	94239	IMAGE:1917941
Nucleosome	94239	IMAGE:248734
Nucleosome	94239	IMAGE:322460
Nucleosome	94239	IMAGE:451649
Nucleosome	9555	IMAGE:1657118
Nucleosome	9555	IMAGE:240990
Nucleosome	9555	IMAGE:843075
ORC	23594	IMAGE:199024
ORC	23595	IMAGE:260336
ORC	4998	IMAGE:121154
ORC	4998	IMAGE:121341
ORC	4998	IMAGE:194236
ORC	4999	IMAGE:295630
ORC	5000	IMAGE:1535410
ORC	5000	IMAGE:878707
ORC	5001	IMAGE:1007141
ORC	5001	IMAGE:1007223

Protein complex	Gene ID	Clone ID
ORC	5001	IMAGE:1585549
PD	1737	IMAGE:124252
PD	1737	IMAGE:271006
PD	1738	IMAGE:1542716
PD	1738	IMAGE:1850936
PD	1738	IMAGE:417957
PD	1738	IMAGE:813648
PD	5162	IMAGE:1639456
PD	5162	IMAGE:826077
PD	5163	IMAGE:1645668
PD	5163	IMAGE:1942432
PD	5163	IMAGE:361190
PD	5163	IMAGE:436761
PD	5164	IMAGE:49860
PD	5164	IMAGE:509589
PD	5165	IMAGE:278242
PD	8050	IMAGE:23576
PD	8050	IMAGE:25156
PD	8050	IMAGE:279665
Proteasome	5682	1292733
Proteasome	5682	IMAGE:134544
Proteasome	5682	IMAGE:298775
Proteasome	5682	IMAGE:451121
Proteasome	5683	IMAGE:155434
Proteasome	5683	IMAGE:366104
Proteasome	5684	IMAGE:1032540
Proteasome	5684	IMAGE:814246
Proteasome	5685	IMAGE:531862
Proteasome	5685	IMAGE:645184
Proteasome	5686	IMAGE:897952
Proteasome	5687	IMAGE:1461316
Proteasome	5687	IMAGE:1505686
Proteasome	5687	IMAGE:509495
Proteasome	5688	IMAGE:2054635
Proteasome	5688	IMAGE:435160
Proteasome	5689	IMAGE:82131
Proteasome	5690	IMAGE:122241
Proteasome	5690	IMAGE:593838
Proteasome	5690	IMAGE:731223
Proteasome	5691	IMAGE:951233
Proteasome	5693	IMAGE:1460110
Proteasome	5693	IMAGE:293675
Proteasome	5693	IMAGE:294485
Proteasome	5694	IMAGE:529861
Proteasome	5695	IMAGE:428043
Proteasome	5695	IMAGE:843352
Proteasome	5701	IMAGE:396272
Proteasome	5701	IMAGE:684655
Proteasome	5702	IMAGE:1602493

Protein complex	Gene ID	Clone ID
Proteasome	5702	IMAGE:712916
Proteasome	5704	IMAGE:756157
Proteasome	5704	IMAGE:810558
Proteasome	5705	IMAGE:2050827
Proteasome	5706	IMAGE:1932026
Proteasome	5706	IMAGE:198614
Proteasome	5706	IMAGE:767049
Proteasome	5706	IMAGE:771900
Proteasome	5709	IMAGE:66377
Proteasome	5709	IMAGE:815861
Proteasome	5714	IMAGE:810550
Proteasome	5717	IMAGE:1553306
Proteasome	5717	IMAGE:179443
Proteasome	5717	IMAGE:450943
Proteasome	5717	IMAGE:566474
Proteasome	5718	IMAGE:823598
Proteasome	5719	IMAGE:1690879
Proteasome	5719	IMAGE:1696289
Proteasome	5719	IMAGE:2306804
RFC	5981	IMAGE:214537
RFC	5981	IMAGE:289787
RFC	5981	IMAGE:50648
RFC	5982	IMAGE:860000
RFC	5983	IMAGE:256260
RFC	5983	IMAGE:277112
RFC	5983	IMAGE:433683
RFC	5983	IMAGE:757158
RFC	5984	IMAGE:203275
RFC	5984	IMAGE:309288
RNA Pol II	5430	IMAGE:1033509
RNA Pol II	5430	IMAGE:1895032
RNA Pol II	5430	IMAGE:430236
RNA Pol II	5430	IMAGE:740130
RNA Pol II	5431	IMAGE:121580
RNA Pol II	5431	IMAGE:1876492
RNA Pol II	5431	IMAGE:293063
RNA Pol II	5431	IMAGE:295551
RNA Pol II	5431	IMAGE:462603
RNA Pol II	5432	IMAGE:770391
RNA Pol II	5433	IMAGE:49548
RNA Pol II	5434	IMAGE:469369
RNA Pol II	5435	IMAGE:767817
RNA Pol II	5436	IMAGE:109269
RNA Pol II	5436	IMAGE:740672
RNA Pol II	5437	IMAGE:2168667
RNA Pol II	5438	IMAGE:280801
RNA Pol II	5438	IMAGE:283615
RNA Pol II	5440	IMAGE:813410
RNA Pol III	10621	IMAGE:263059

Protein complex	Gene ID	Clone ID
RNA Pol III	10623	IMAGE:711959
RNA Pol III	51728	IMAGE:511632
RNA Pol III	55703	IMAGE:199644
RNA Pol III	55703	IMAGE:488683
RNA Pol III	661	IMAGE:1557660
SCF	6500	IMAGE:1585040
SCF	6500	IMAGE:262044
SCF	6500	IMAGE:291464
SCF	8454	IMAGE:1751841
SCF	8454	IMAGE:1842546
SCF	8454	IMAGE:450883
SCF	8454	IMAGE:841093
SCF	9978	IMAGE:200144
SCF	9978	IMAGE:433666
SNARE	10282	IMAGE:1010015
SNARE	10282	IMAGE:1010420
SNARE	10282	IMAGE:203351
SNARE	10490	IMAGE:1915012
SNARE	10652	IMAGE:810762
SNARE	26984	IMAGE:324745
SNARE	6811	IMAGE:2047655
SNARE	6811	IMAGE:297099
SNARE	6811	IMAGE:787857
SNARE	9527	IMAGE:324694
SNARE	9527	IMAGE:625863
SNARE	9570	IMAGE:130773
SNARE	9570	IMAGE:145388
SNARE	9570	IMAGE:1750504
SNARE	9570	IMAGE:1943138
SNARE	9570	IMAGE:239708
SNARE	9570	IMAGE:66391
SNARE	9570	IMAGE:841697
SRP	6728	IMAGE:1894034
SRP	6728	IMAGE:754998
SRP	6729	IMAGE:1846354
SRP	6729	IMAGE:196638
SRP	6729	IMAGE:950430
SRP	6730	IMAGE:1952876
SRP	6730	IMAGE:626358
SRP	6730	IMAGE:814791
SRP	6731	IMAGE:1755103
SRP	6731	IMAGE:321510
SRP	6731	IMAGE:358713
SRP	6731	IMAGE:811842
SRP	6731	IMAGE:814702
SRS	2197	IMAGE:1985178
SRS	2197	IMAGE:2249522
SRS	51065	IMAGE:590338
SRS	6187	IMAGE:429128

Protein complex	Gene ID	Clone ID
SRS	6188	IMAGE:487445
SRS	6193	IMAGE:50918
SRS	6193	IMAGE:809578
SRS	6201	IMAGE:2321118
SRS	6202	IMAGE:62257
SRS	6202	IMAGE:971367
SRS	6203	IMAGE:1861627
SRS	6204	1293191
SRS	6204	IMAGE:111136
SRS	6204	IMAGE:139641
SRS	6204	IMAGE:1422366
SRS	6204	IMAGE:1468533
SRS	6204	IMAGE:1580126
SRS	6204	IMAGE:1629289
SRS	6204	IMAGE:1636549
SRS	6204	IMAGE:1639217
SRS	6204	IMAGE:1683873
SRS	6204	IMAGE:1733293
SRS	6204	IMAGE:1841990
SRS	6204	IMAGE:1848093
SRS	6204	IMAGE:1875670
SRS	6204	IMAGE:1901173
SRS	6204	IMAGE:1915149
SRS	6204	IMAGE:1915897
SRS	6204	IMAGE:1917897
SRS	6204	IMAGE:2252417
SRS	6204	IMAGE:251195
SRS	6204	IMAGE:280967
SRS	6204	IMAGE:46977
SRS	6204	IMAGE:487035
SRS	6204	IMAGE:509949
SRS	6204	IMAGE:565905
SRS	6204	IMAGE:780997
SRS	6206	IMAGE:2271240
SRS	6207	IMAGE:857243
SRS	6208	IMAGE:1697368
SRS	6208	IMAGE:741522
SRS	6208	IMAGE:756820
SRS	6217	IMAGE:853151
SRS	6223	IMAGE:192242
SRS	6223	IMAGE:1926032
SRS	6223	IMAGE:1983719
SRS	6224	IMAGE:1699381
SRS	6224	IMAGE:2007305
SRS	6224	IMAGE:80265
SRS	6227	IMAGE:2272597
SRS	6228	IMAGE:291974
SRS	6228	IMAGE:49392
SRS	6228	IMAGE:511882

Protein complex	Gene ID	Clone ID
SRS	6228	IMAGE:511991
SRS	6228	IMAGE:868308
SRS	6229	IMAGE:1055201
SRS	6229	IMAGE:1637296
SRS	6229	IMAGE:461071
SRS	6230	IMAGE:454905
SRS	6230	IMAGE:896930
SRS	6235	IMAGE:1950606
SWI-SNF	51412	IMAGE:113394
SWI-SNF	55193	IMAGE:1556449
SWI-SNF	55193	IMAGE:214744
SWI-SNF	55193	IMAGE:239615
SWI-SNF	55193	IMAGE:296883
SWI-SNF	55193	IMAGE:427811
SWI-SNF	55193	IMAGE:811981
SWI-SNF	55193	IMAGE:813644
SWI-SNF	6595	IMAGE:142540
SWI-SNF	6595	IMAGE:1640707
SWI-SNF	6595	IMAGE:1848857
SWI-SNF	6597	IMAGE:1011163
SWI-SNF	6597	IMAGE:1011375
SWI-SNF	6597	IMAGE:897890
SWI-SNF	6598	IMAGE:121605
SWI-SNF	6598	IMAGE:1467420
SWI-SNF	6598	IMAGE:586715
SWI-SNF	6598	IMAGE:781018
SWI-SNF	6599	IMAGE:490813
SWI-SNF	6601	IMAGE:160838
SWI-SNF	6602	IMAGE:241736
SWI-SNF	6603	IMAGE:741067
SWI-SNF	6604	IMAGE:360047
SWI-SNF	8289	IMAGE:1881595
SWI-SNF	8289	IMAGE:461442
SWI-SNF	8289	IMAGE:810843
SWI-SNF	86	IMAGE:753400
TAFIID	10629	IMAGE:1670689
TAFIID	10629	IMAGE:268803
TAFIID	27097	IMAGE:377521
TAFIID	54457	IMAGE:743116
TAFIID	6872	IMAGE:222460
TAFIID	6872	IMAGE:299093
TAFIID	6872	IMAGE:454440
TAFIID	6873	IMAGE:136730
TAFIID	6873	IMAGE:462691
TAFIID	6877	IMAGE:1587429
TAFIID	6878	IMAGE:33438
TAFIID	6879	IMAGE:242700
TAFIID	6879	IMAGE:365930
TAFIID	6879	IMAGE:795850

Protein complex	Gene ID	Clone ID
TAFIID	6880	IMAGE:564197
TAFIID	6881	IMAGE:723972
TAFIID	6882	IMAGE:306444
TAFIID	6883	IMAGE:509588
TAFIID	6908	IMAGE:280735
TRAPP	122553	IMAGE:745426
TRAPP	126003	IMAGE:668007
TRAPP	126003	IMAGE:878403
TRAPP	27095	IMAGE:460580
TRAPP	27095	IMAGE:780977
TRAPP	51399	IMAGE:897153
TRAPP	58485	IMAGE:135710
TRAPP	58485	IMAGE:731014
TRAPP	79090	IMAGE:2322297
VHL	6923	IMAGE:884692
VHL	7428	IMAGE:234856
VHL	8453	IMAGE:788247
VHL	9978	IMAGE:200144
VHL	9978	IMAGE:433666
