# Supplementary material for: A new computational approach to analyze human protein complexes and predict novel protein interactions
Source: Genome Biol. 2007 Dec 4;8(12):R256. doi: 10.1186/gb-2007-8-12-r256 (PMC2246258; doi:10.1186/gb-2007-8-12-r256)
Supplement: Additional data file 12 — Supplemental Materials and methods. [file gb-2007-8-12-r256-S12.doc]

## Sub- Gene Ontology analysis of multiprotein complexes

We explain here how we evaluated the p-values listed in the additional file 7.

First we associated to each gene in the dataset its GO terms, according to Ensembl database (ensemble_mart_35 available at http://www.ensembl.org/index.html), and to these keywords we add their ancestors up to the 2^nd^ level (root = 0 level). Then, for each complex, we extracted GO terms at which at least one of its genes is annotated and we counted the number of genes annotated at each recovered Biological Process term. To assess the significance of the observed GO terms enrichment, we computed the probability to obtain by chance at least the same number of genes annotated to each term through the hypergeometric distribution according to formula (2)

where

*N* = Number of genes in the analyzed dataset annotated at the selected GO (i.e. Biological Process)

*M_w_* = Number of genes in the analyzed dataset annotated at GO term *w*

*n_c_* = Number of genes of a selected protein complex *c* annotated at the selected GO

*f_wc_* = Number of genes of the selected protein complex *c* annotated at GO term *w*.

These are the four entries listed in the additional file 13 in the columns from 5 to 9.

## Sub- PAK1-mRFP construct

MRFP-N1 expression vector has been generated by replacing enhanced green fluorescent protein of pEGFP-N1 expression vector (Clontech) with mRFP. mRFP was excised from the pRSET B cloning vector (a kind gift from Roger Tsien University of California, San Diego) using *Bam*HI and *Eco*RI and cloned into pEGFP-N1 from which EGFP had been excised with *Bam*HI and *Not*I.

To generate tagged PAK1, p21-activated kinase was excised from the mammalian expression vector pCDNA3-EGFP (a gift from Dr. G. Bokoch, The Scripps Research Institute, La Jolla, California) using the *Kpn*I restriction site of pCDNA3-EGFP, which contains the hPAK1 coding sequence and EGFP tag. The product was cloned into mRFP-N1 excised with *Kpn*I.

## Sub- Peptides analysis

Similarities between peptides selected with phage-display and known protein sequences have been defined as following:

First homologies were evaluated by searching non-redundant human protein databases with the Blast program (www.ncbi.nlm.nih,.gov/Blast) and alignments with best E score selected.

All 8 aa sequences aligned with the query were selected, aligned by using CLUSTALW (<http://www.ebi.ac.uk/clustalw/>) and used as input for HMMER programs to build up a model for searching similarities between protein sequences. RefSeq for H. sapiens (<ftp://ftp.ncbi.nih.gov/refseq/>) has been used as database.
